# Inspection of stability of a general roll-damping of a ship via non-perturbative approach

**DOI:** 10.1038/s41598-026-38505-8

**Published:** 2026-02-23

**Authors:** Galal M. Moatimid, Mona A. A. Mohamed, M. K. Abohamer

**Affiliations:** 1 Department of Mathematics, Faculty of Education, Ain Shams University, Cairo, 11566 Egypt; 2 Department of Engineering Physics and Mathematics, Faculty of Engineering, Tanta University, Tanta, 31734 Egypt

**Keywords:** Rolling vessel, Nonlinear analysis, Non-perturbative approach, He’s frequency formula, Multiple time-scales method, Bifurcation analysis, Engineering, Mathematics and computing, Physics

## Abstract

The current study explores nonlinear stability in vessels with roll-damping to ensure safety in realistic maritime conditions, thereby expanding stability. It develops a generalized model that considers operating situations, nonlinearities, and hydrodynamic factors. One degree of freedom (1DOF) of nonlinear ship dynamics is included in the exiting model. The prototypical incorporates inertia, damping, restoring forces, and external forces. The objective is to examine the responsible non-perturbative method (NPA) in determining periodic response of a damped and conservative coupled system. In contrast to all other traditional perturbation techniques, NPA’s goal is to convert a weakly oscillator of nonlinear of ordinary differential equation (ODE) into a linear one without consuming Taylor expansion. A strong agreement with original numerical solution (NS) is validated. Using Galerkin technique, the inquiry produces an advanced, comparable linear ODE. Quantitative comparisons verify that final solution agrees with advanced solution. The resonance area, situated within stability zone and exhibiting complex relationships between forces in the system, is influenced by all physical properties. An approximate solution up to the second order is found by applying the multiple-time scales method (MTSM), which evaluates the system’s stability configuration and highlights both the stable and unstable features. Changes in bifurcation parameters have an impact on curvature of the bifurcation curve. Additionally, phase portraits, Poincaré maps, and bifurcation diagrams are used to perform a bifurcation analysis of the designated models and identify the different motions of the system.

**Supplementary Information:**

The online version contains supplementary material available at 10.1038/s41598-026-38505-8.

## Introduction

Several researchers have successfully represented the rolling ship movement using a semi-empirical nonlinear ODE^1^. Excitations are frequently shown as either a time-varying coefficient in an ODE or an inhomogeneous term in the governing equation of a 1DOF oscillator. These kinds of excitations are frequently referred to as external and parametric excitations. Nonlinearity introduces several phenomena in a linear analysis. Multiple jumps, natural frequency shift, frequency entrapment, period-multiplying bifurcation, sub-harmonic and ultra-sub-harmonic resonance, and dynamic chaos are a few of these^2^. The nonlinear established harmonic roll of a ship was solved nearly to second order in normal beam seas^3^. One contrasts the perturbation solutions with those derived from NSs of nonlinear governing ODEs. Compared to the first-order expansion, the second-order expansion predicted a peak roll angle and associated frequency that was more in line with NS. Researchers looked into a nonlinear oscillator that was activated by external and parametric functions at the same time^4^. Lyapunov stability is used to assess the stability of the suggested solution. The nonlinear motion equation of ship rolling in random sea conditions is created by analyzing nonlinear restoring moment and nonlinear damping torque. The problem is solved in the time domain using Runge–Kutta method, which is also used to model free decay process and temporal history of ship rolling caused by random waves. Nonlinear damping and rectifying lever qualities were predicted using experimental data, and comparison with empirical performance was reported^5^. Qualitative prediction techniques are used to investigate stability of periodic motion, namely the possibility of capsizing. A new approach in measuring ship stability in wave conditions was proposed by certain results of nonlinear dynamics of driven oscillators^6^. Compared to a strategy based on the stability analysis of steady-state rolling movements, which may be dangerously non-conservative, the approach has combined advantages of being more applicable and theoretically simpler^7^. A ship rolling in longitudinal waves was used to illustrate the qualitative response^8^. The qualitative change that occurs in stable solutions of a ship model as one parameter is progressively changed and estimated using analytical and numerical techniques. The relationship between detuning parameter and the steady-state response amplitude is illustrated by NSs.

According to dynamical system theory, parametric rolling, a risk of oceangoing ships, is caused by changes in restoring moment brought on by waves. Parametric rolling has been known to cause serious accidents on both pure automotive carriers and container ships. Researchers looked on the risky phenomenon of parametric rolling in rough waters^9^. The instability mechanism was explained theoretically in a novel way. Second-generation intact stability requirements of vessel roll motion were being developed by international maritime organization^10^. The probability density function of roll motion during parametric rolling in irregular waves was obtained using stochastic averaging approach^11^. Under parametric excitation, a rolling equation with 1DOF was developed^12^. The righting arm was calculated numerically using ship theory, considering different roll angles, wave heights, and wave phase angles. In righting arm, an analytical expression is derived using the least squares method. One risky dynamic phenomenon is parametric rolling. Assessing probability of particular dynamic ship behavior with respect to a determined threshold level is crucial in determining a vessel’s safety during a hazardous occurrence. By solving the moment equations, moment values were obtained^13^. There are several documented instances of parametric rolling on container ships and pure automobile carriers. Researchers looked on dangerous phenomenon of parametric rolling in irregular waters^14^. The stability of genesis of the system, which corresponds to upright posture of vessel, was examined. Within the framework of second-generation intact stability requirements, ship operators needed simplified operating instructions on how to choose forward speed and heading angle in relation to waves, particularly with regard to parametric rolling^15^. The stochastic modification of coefficients in the governing equations of motion was credited in numerical calculations^16^. Furthermore, particular contributions to the analysis of stabilization and destabilization brought on by noise disturbances were reported in the field of control research. While damping coefficients of the compartments show a complex connection with changes in roll amplitude and frequency, the damping coefficients of entire damaged vessel and outer hull increase as roll amplitude and frequency rise^17^. An outline of how the operating profile may affect safety considerations during marine vessel navigation was given^18^. To describe the transient response characteristics of external disturbance torque and control stability torque of maritime crane, a nonlinear system model that included rope change rate was created^19^. Ships may experience cargo shifts, damage, and challenging working conditions of their crews due to parametric rolling in high seas. Its occurrence was predicted, and the factors influencing its start and magnitude were examined^20^.

A ship’s rolling motion is an example of a dynamic system, which is defined by interplay of forces and torques applied to ship as a result of outside disturbances. Nonlinear ODEs that outline ship’s angular displacement, velocity, and acceleration on time can quantitatively depict this motion. The stability and natural frequency of roll of vessel are influenced by its buoyancy, geometry, and mass distribution, which may cause oscillatory behavior. Engineers estimate performance in a variety of maritime situations and advance stabilization performances, such as gyroscopic stabilizers or anti-roll fins, by understanding rolling motion in a dynamic system. It ensures effectiveness and safety of marine operations. The specific asymptotic formulas for low-strength sounds are established using the Homotopy perturbation technique (HPM). Unfortunately, using this insignificant parameter in both approaches led to incorrect outcomes. The HPM was used by Moatimid et al.^21–24^ to study a variety of problems in fluid dynamics as well as dynamical systems. Finding small parameter that enables a more accurate formulation of basic ODE in real-world applications is a crucial firstly step in any asymptotic or perturbation approach. HFF is a straightforward and efficient method in keeping nonlinear oscillators. The recent study gave engineers a new method of effectively and consistently using HFF to investigate nonlinear vibration systems. In fractal structures, a frequency computation based on the HFF was created. Consequently, the original system’s performance will be evaluated more precisely^25–43^. The goal of this simplification is to reduce the average divergence between the two systems by transforming the problem from a nonlinear ODE to a linear one. Additionally, NPA differs greatly from conventional perturbation techniques such as MTSM or HPM. NPA is more than merely an alternative technique, and the following points highlight some of its unique faces:

1. The idea comes from HFF and is objective. The ancient China mathematicians were unquestionably the first to make this discovery.
2. This idea’s goal is to create a linear ODE that is comparable to nonlinear one that controls the ship-rolling phenomenon.
3. The consistency of the two ordinary ODEs is ensured by their numerical compatibility and Tabular representation.
4. Every parameter in the nonlinear ODE is included in the linear one.
5. The nonlinear ODE has not been analytically addressed, according to NPA.
6. Unlike the conventional perturbation approach, NPA employs a special methodology to address restoring forces; it is not classified as a perturbation technique.
7. All perturbation practises, including the well-known one called MTSM; use Taylor expansion to help calculate the restoring forces. This shortcoming was disregarded via NPA.
8. Additional combinations of dynamical systems deemed significant, effective, and persuasive may be incorporated into the NPA.

The accuracy and extensiveness of roll-damping studies have also been greatly enhanced by developments in experimental methods and data collection systems, enabling in-depth examination of both linear and nonlinear damping effects over a broad range of frequencies and amplitudes. Researchers are now able to quantify the impact of intricate hydrodynamic interactions, including viscous flow separation, bilge vortex shedding, and wave radiation, on damping characteristics thanks to high-fidelity measurements derived from model experiments and full-scale trials. These discoveries aid in bridging the gap between theoretical predictions and actual ship behavior by enhancing semi-empirical and computational models. Practically speaking, a better understanding of roll-damping aids in the development of adaptive control systems, sophisticated anti-roll devices, and optimized hull geometry for ship stabilization in dynamic sea conditions. In order to improve ship stability assessment and encourage innocuous, more effective maritime operations, it is crucial to incorporate experimental results into design and simulation frameworks. NPA is not just a different approach; the following points outline unique features of this approach. To enhance the clarity of the article’s performance, the next parts are arranged as follows: **§ 2** describes the problem formulation. The NPA’s sophisticated methodology is also covered in this section. The validation approach of the particular situation is shown in **§ 3**. A procedure in identifying an advanced linear ODE is found in **§ 4**. The stability analysis, time history and phase plane of the particular situation without the exciting force are illustrated in **§ 5**. The solution of the advanced linear ODE is investigated by employing the MTSM through **§ 6**. **§ 7** presents the bifurcation of the initial nonlinear ODE. Finally, a summary of the main conclusions is given in **§ 8**.

## Construction of concern

The rolling of a ship in longitudinal waves can be defined by a nonlinear ODE because of nonlinear restoring forces and external stimulation of waves. To evaluate ship’s rolling motion in reaction to outside stimuli, such as waves, a nonlinear dynamical system analysis is utilized. Parametric roll is a unique nonlinear phenomenon in setting of a rolling vessel, in contrast to other nonlinear phenomena like broaching, pure resonance, or nonlinear damping. When wave-induced changes in body geometry or buoyancy distribution cause recurring variations in stability parameters of vessel, like metacentric height, it occurs during maritime navigation. If there is no external driving force at the natural frequency, these changes may coincide with roll frequency of vessel itself, causing in a significant increase in roll amplitude. While other nonlinear processes, such as broaching, require abrupt, frequently chaotic directed instabilities related to hydrodynamic forces, pure resonance shows direct external stimulation at natural roll frequency without changes to stability characteristics. Unlike parametric roll, these phenomena do not depend on changeable stability factors of their evolution. The dominant assumption is that vessel is being affected by an external wave-induced moment.

**Fig. 1:**
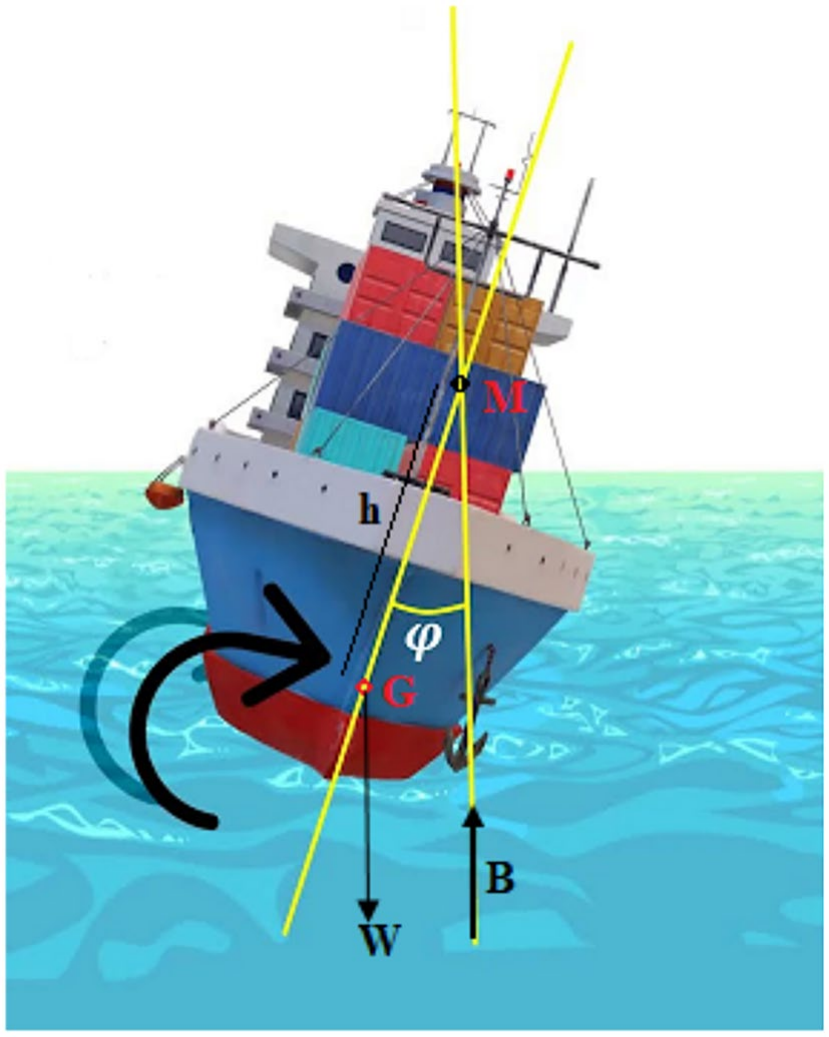
Represents a prototype of the rolling ship model.

As stated in the problem statement, the rolling motion of a ship with a known moment of inertia around its longitudinal axis, represented by $I$, is caused by wave forces; the damping force becomes $D(\varphi ,\dot{\varphi })$, and up to the seventh degree, there are both linear and nonlinear restoring forces $K(\varphi )$. The goal is to show how the rolling angle $\varphi (t)$ is affected by both external and intrinsic factors of the dynamical system, while remaining constant over time. Examine a ship traversing waves at a definite advancing speed; Fig. 1 illustrates the conception. A ship in rolling motion is seen in cross-section in this illustration. Archimedes’ principle states that the buoyancy force $B$ exerted on an object is equal to the weight of the liquid it displaces. The met center $M$ is the intersection of line of action of buoyancy force with ship’s centerline. The distance $h$ between the mass center $G$ and $M$ indicates the metacentric height, the point at which the buoyant force acts when the vessel is tilted by a small angle, and which determines the initial static stability based on the metacentric height. Our proposed model extends beyond linear assumptions by incorporating general roll damping and nonlinear restoring characteristics, providing a more realistic representation of roll motion, especially in extreme sea states. The NPA offers insights into the global behavior of the system, such as basin erosion, bifurcation, and instability thresholds, which are not captured in conventional linear or weakly nonlinear models. The model can serve as a diagnostic and predictive tool in assessing a vessel’s susceptibility to parametric rolling, broaching, or large amplitude instability, supporting both design evaluation and operational decision-making.

The 1DOF equation of the damping ship rolling oscillator (DSRO) is generally represented as an ODE of the form^38,39,44^:

1 $$I\ddot{\varphi } + D(\varphi ,\dot{\varphi }) + K(\varphi ) = f(t)$$

where $\varphi$ is roll angle, $I$ is moment of inertia, $D(\varphi ,\dot{\varphi })$ is damping force, $K(\varphi )$ is linear and nonlinear restoring forces, and $f(t)$ is external force.

For this purpose, assuming that:

2 $$D(\varphi ,\dot{\varphi })/I = 2\mu \dot{\varphi } + \gamma_{1} \varphi^{2} \dot{\varphi } + \gamma_{2} \dot{\varphi }^{3}$$

3 $$K(\varphi )/I = (\omega_{0}^{2} + \delta_{1} )\varphi + \delta_{3} \varphi^{3} + \delta_{5} \varphi^{5} + \delta_{7} \varphi^{7}$$

and

4 $$f(t)/I = F\cos \sigma t$$

Equation (1) is then be rewritten as:

5 $$\ddot{\varphi } + 2\mu \dot{\varphi } + \lambda_{1} \varphi^{2} \dot{\varphi } + \lambda_{2} \dot{\varphi }^{3} + (\omega_{0}^{2} + \delta_{1} )\varphi + \delta_{2} \varphi^{3} + \delta_{3} \varphi^{5} + \delta_{4} \varphi^{7} = F\cos \sigma t$$

The coefficients included in Eqs. (2)–(4) are defined as: $\delta_{1}$,$\delta_{2}$, $\delta_{3}$ and $\delta_{4}$ are constants of nonlinear restoring force, $\omega_{0}^{2}$ is square of natural frequency,$2\mu$,$\gamma_{1}$, and $\gamma_{2}$ are linear and nonlinear damping factors. Moreover, $\sigma$ and $F$ are the frequency of external periodic forcing and its amplitude. Correspondingly, Eq. (5) is considered in conjunction with the initial conditions (ICs):

6 $$\varphi (0) = A\,\,\& \,\,\dot{\varphi }(0) = 0$$

where $A$ is the initial amplitude.

**Fig. 2:**
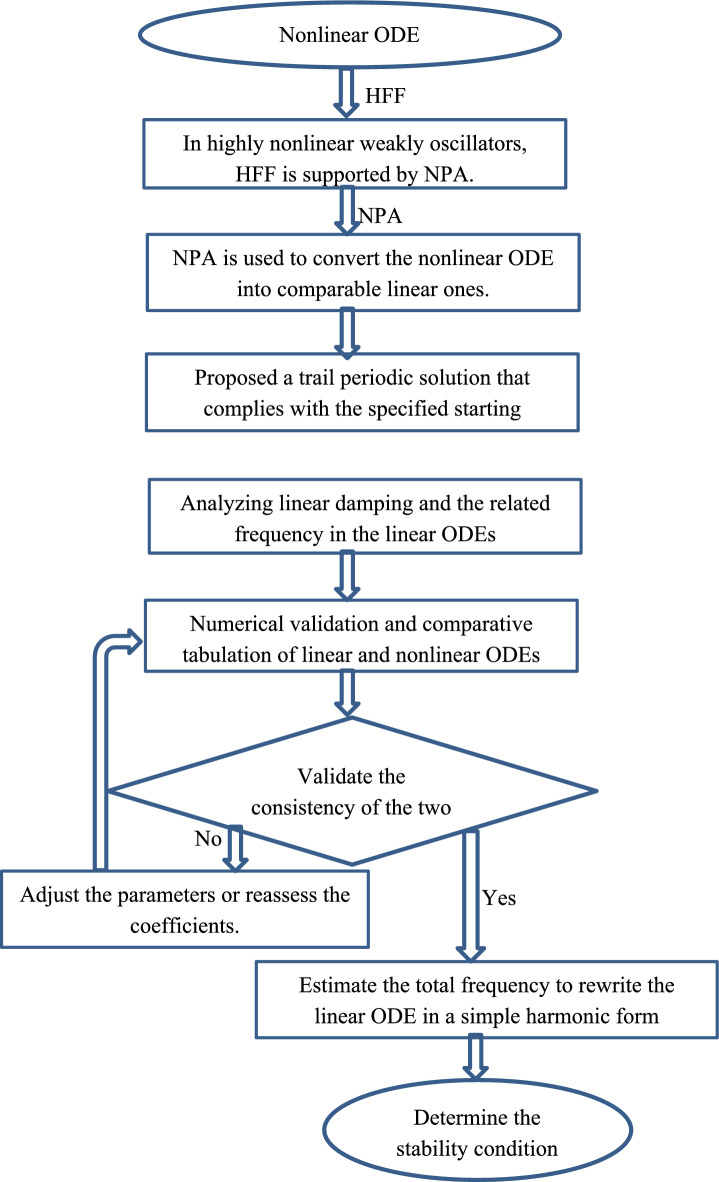
Demonstrating the frameworks of NPA’s procedure.

In analytical and numerical techniques, the trial solution is an initial hypothesis or an assumed structure of solution derived from the known properties of the issue, particularly in solving problems or ODE. By reducing the problem to a more convenient form or providing a basis of iterative processes that improve response via successive approximations, this assumption is meant to expedite the problem-solving process. The current study’s originality relates to consequences of NPA. The lack of compact solutions of all weakly nonlinear ODEs is widely known; hence, perturbation techniques are crucial. The complexity of the processes of all traditional perturbation methods, including the MTSM, is well known. Accordingly, NPA represents a novel approach in addressing these challenging issues. NPA doesn’t use Taylor expansion of trigonometric functions, unlike all other traditional methods. To improve reader comprehension, a flowchart that depicts the methodological approach is added, as mentioned before. Figure 2 represents a flowchart of the steps of NPA. When applied to nonlinear systems, particularly ship rolling motion, which is inherently nonlinear due to restoring forces, damping, and wave excitations, NPA technique has significant physical relevance. In contrast to traditional perturbation techniques, NPA offers a more rapid and logical method in comprehending complicated dynamics. NPA is a semi-analytical approach that applies conventional perturbation methods to systems that are weakly and moderately nonlinear. Engineers can use it to:

- Recognize how rolling system behaves close to equilibrium.
- Estimate stability of complicated nonlinear ODEs without doing a thorough solution.
- Examine how stability, amplitude, and frequency of roll motion are impacted by nonlinearities.

The trial (guessing) solution may be expressed, as demonstrated before^27–43^, as follows:

7 $$\tilde{u} = A\cos \Omega t$$

where $\Omega$ is the frequency that is named as total frequency, which will be assessed subsequently.

It should be noted that the trial solution is proposed like a periodic solution in order to obtain an oscillator linear equivalent ODE. The target of NPA is to convert the nonlinear ODE as given in Eq. (5) into another linear equivalent one, as follows:

8 $$\ddot{u} + 2\mu_{eqv} \dot{u} + \omega_{eqv}^{2} u = F\cos \sigma t$$

where $\mu_{eqv}$ and $\omega_{eqv}^{2}$ are the corresponding damping and frequency, respectively.

Understanding their physical relevance within the framework of the specified forced oscillator is crucial. The equations presented in (5) and (6) describe effective parameters that characterize the system’s response by averaging nonlinear effects across an entire oscillation cycle. The equivalent damping coefficient signifies the total energy dissipation of the system, incorporating both linear and nonlinear damping effects. The equivalent natural frequency denotes the system’s effective stiffness, integrating contributions from both linear restoring forces and nonlinear elements. These similar parameters provide a streamlined yet precise characterization of oscillator’s behavior, permitting stability and resonance analysis while maintaining essential nonlinear dynamics. Therefore, following^27–43^, the corresponding damping and the corresponding frequency may be achieved as follows:

9 $$\mu_{eqv} = \frac{{\int\limits_{0}^{2\pi /\Omega } {\dot{\tilde{u}}F_{1} (\dot{\tilde{u}})dt} }}{{\int\limits_{0}^{2\pi /\Omega } {\dot{\tilde{u}}^{2} dt} }} = \mu + \frac{1}{8}A^{2} (\gamma_{1} + 3\gamma_{2} )$$

and

10 $$\omega_{eqv}^{2} = \frac{{\int\limits_{0}^{2\pi /\Omega } {\tilde{u}F_{2} (\tilde{u})dt} }}{{\int\limits_{0}^{2\pi /\Omega } {\tilde{u}^{2} dt} }} = \omega_{0}^{2} + \delta_{1} + \frac{3}{4}\delta_{2} A^{2} + \frac{5}{8}\delta_{3} A^{4} + \frac{35}{{64}}\delta_{4} A^{6}$$

where

11 $$\left\{ \begin{gathered} F_{1} (\dot{\varphi }) = 2\mu \dot{\varphi } + \gamma_{1} \varphi^{2} \dot{\varphi } + \gamma_{2} \dot{\varphi }^{3} , \hfill \\ F_{2} (\varphi ) = (\omega_{0}^{2} + \delta_{1} )\varphi + \delta_{2} \varphi^{3} + \delta_{3} \varphi^{5} + \delta_{4} \varphi^{7} , \hfill \\ \end{gathered} \right.$$

are odd damping, and odd secular terms, respectively.

The integrals of Eq. (5) possess substantial scientific meanings of energy dynamics of specified forced oscillator. The integral in Eq. (5) signifies the time-averaged impact of damping across a single oscillation cycle, incorporating both linear and nonlinear damping effects. It accurately describes the manner in which the system dumps energy through internal resistive forces, affecting amplitude decay and stability of oscillations. Simultaneously, integral in Eqs. (9) and (10) summarize time-averaged effect of restoring forces, encompassing both linear stiffness and nonlinear factors, on system’s natural frequency. This integral assesses alterations in the oscillator’s effective frequency resulting from nonlinearities, clarifying the relationship between external forces and internal system variables that influence the overall dynamic response. By integrating these numbers across a complete cycle, these formulations guarantee that equivalent parameters precisely represent the oscillator’s long-term behavior.

For the investigation of a forced DSRO response, the nonlinear system is approximated with a similar linear representation using the analogous linearization approach. Evaluating system’s stability and resonance characteristics in reaction to outside influences is the main goal. The Galerkin approach is used to generate an approximate analytical solution, which allows a detailed approval of resonance conditions. The technique first approximates analogous parameters, such as natural frequency and damping coefficient, using a trial solution. The nonlinear oscillator may be transformed into matching forced linear oscillator recognitions to these characteristics. By using the conventional formula approach, Eq. (8) may be transformed into its standard form. Accordingly, the following modification might be proposed.

12 $$u = e^{{ - \mu_{eqv.} t}} v(t)$$

Now $v(t)$ is the required periodic function of time to be estimated.

One may obtain the differential formulation that governs the unknown function $v(t)$ by substituting Eq. (12) into Eq. (8):

13 $$\ddot{v} + \Omega^{2} v = e^{{\mu_{eqv.} t}} F\cos \sigma t$$

which is the normal simple harmonic equation with the frequency $\Omega$ that is specified as:

14 $$\begin{gathered} \Omega^{2} = \omega_{eqv}^{2} - \mu_{eqv}^{2} \hfill \\ \,\,\,\,\,\,\, = ( - 256\mu^{2} + 64\omega_{0}^{2} - A^{2} (4A^{2} \gamma_{1}^{2} + 64\mu \gamma_{1} + 192\mu \gamma_{2} + 24A^{2} \gamma_{1} \gamma_{2} + \hfill \\ \,\,\,\,\,\,\,\,\,\,\,\,\,\,\,\,\,\,\,\,\,\,\,\,\,\,\,\,36A^{2} \gamma_{2}^{2} - 64\delta_{1} - 48\delta_{2} - 40A^{2} \delta_{3} - 35A^{4} \delta_{4} ))/64 \hfill \\ \end{gathered}$$

The fundamental precise solution of Eq. (13) is expressed, as concluded by MS, as follows:

15 $$\begin{gathered} u(t) = \left[ {A - \frac{{F\left( {\omega_{eqv}^{2} - \sigma^{2} } \right)}}{{\left( {\left( {\omega_{eqv}^{2} - \sigma^{2} } \right)^{2} + 4\sigma^{2} \mu_{eqv}^{2} } \right)}}} \right]e^{{ - \mu_{eqv} t}} \cos \Omega t + \frac{{F\left( {\left( {\omega_{eqv}^{2} - \sigma^{2} } \right)\cos \sigma t + 2\mu_{eqv} \sigma \sin \sigma t} \right)}}{{\left( {\left( {\omega_{eqv}^{2} - \sigma^{2} } \right)^{2} + 4\sigma^{2} \mu_{eqv}^{2} } \right)}} \hfill \\ \,\,\,\,\,\,\,\,\,\,\,\,\,\left[ {A - \frac{{F\left( {\omega_{eqv}^{2} - \sigma^{2} } \right)}}{{\left( {\left( {\omega_{eqv}^{2} - \sigma^{2} } \right)^{2} + 4\sigma^{2} \mu_{eqv}^{2} } \right)}}} \right]e^{{ - \mu_{eqv} t}} \sin \Omega t\, \hfill \\ \end{gathered}$$

Equation (15) provides an exact analytical solution, which incorporates essential oscillatory behavior of driven oscillator, integrating both damping and nonlinear restorative influences. This expression emphasizes the interaction between system’s inherent dynamics and external periodic stimulation, illustrating temporal evolution of amplitude due to damping and impact of nonlinearities on frequency content. The solution structure indicates the absence of resonance conditions, as division by zero is not present, thereby guaranteeing confined oscillations. This formulation mainly represents the behavior of a linearized approximation of the system, necessitating a refinement of the method of a more accurate characterization of resonance phenomena. To achieve this, a sophisticated trial solution must be employed, considered the system’s nonlinear characteristics in a manner that surpasses traditional linearization methods. This stage is essential of precisely determining resonance conditions and examining stability limits, especially when the system experiences continuous external pushing that may provoke intricate vibrational responses.

As well-known, the basic frequency level indicated by Eq. (13) fails to incorporate the effects of periodic force. To investigate a more sophisticated frequency that encompasses the influence of periodic force, it is essential to improve trial solution described in Eq. (7). This modification is essential in obtaining a sophisticated solution of the linearized equation, namely Eq. (8), rather than depending on the fundamental solution shown in Eq. (15). This technique is essential of an inclusive knowledge of the dynamics involved, especially in analyzing the influence of external periodic belongings on the behavior of the system and stability.

For more convenience, it should be noted that the main restrictions of NPA have been provided to help reader. These limitations may be summarized as follows:

(i) NPA is applicable only for weakly nonlinear second-order oscillators.
(ii) The original ICs remain unaltered.
(iii) For improved precision, the initial amplitude should be less than one.

## Validation approach

For an enhance ease, the correspondence between the nonlinear and linear ODEs may be numerically validated using the subsequent graphic. For this objective, consider the subsequent selection of data:

$$\omega_{0} = 3.0,\,\,\mu = 0.1,\,\,A = 0.2,\,\,F = 0.05,\,\,\sigma = 6,\,\gamma_{1} = 0.1,\,\gamma_{2} = 0.2,\,\delta_{1} = 0.4,\delta_{2} = 0.3,\delta_{3} = {0}{\mathrm{.2,}}\,{\mathrm{and}}\,\delta_{4} = {0}{\mathrm{.1}}$$.

**Fig. 3:**
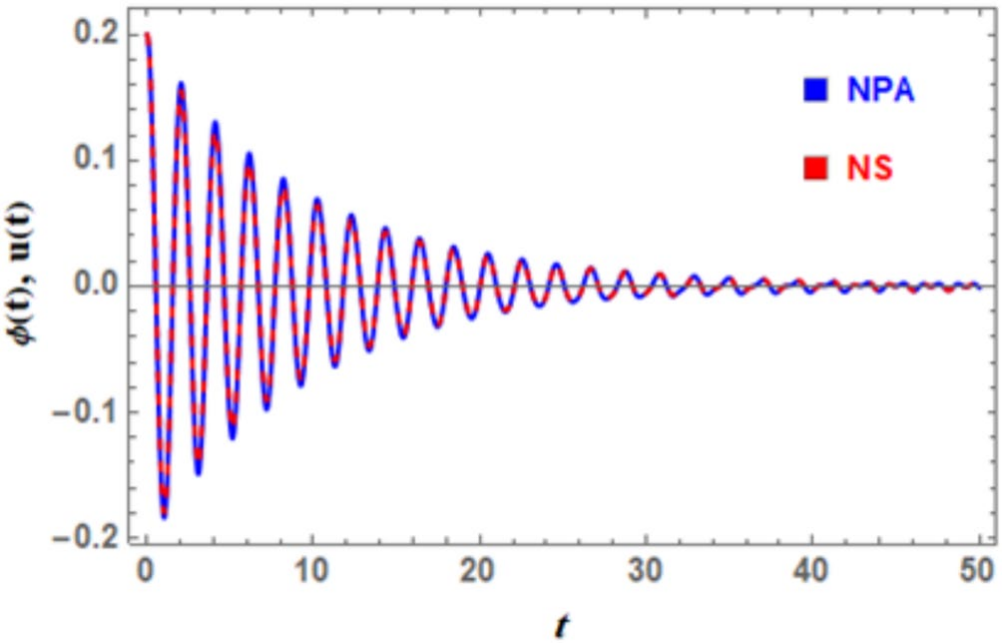
Displays a good identical among the linear equivalent one Eq. (8) and the nonlinear ODE as given in Eq. (5).

In the following, a thorough error analysis comparing the system’s NS, as determined by Eq. (5), with NPA, as determined by Eq. (8), is presented in Fig. 3 and Table 1. The minuscule variations among these solutions demonstrate remarkable exactness and consistency. The effectiveness of the suggested technique in effectively modeling complex dynamical systems is validated by the remarkable correlation between estimated and real values. A thorough comparison of true and estimated values throughout a range of time periods, together with the corresponding absolute and relative uncertainty, is provided in Table 1. By means of a systematic analysis of these numbers, the information offers a conclusive evaluation of accuracy and reliability of employed approximation method. It also highlights how differences between estimated and real results change over time, identifying potential causes of numerical errors such as algorithmic limitations, truncation effects, and computational approximations. This thorough assessment is especially important in cases like nonlinear dynamical systems, where little differences can have a significant impact on system behavior, particularly in the design, operation, and safety assessment of maritime boats.

**Table 1:** Displays a numerical comparison between Eqs. (5) and (8).

t	Nonlinear	Linear	Absolute error	Relative absolute error	Relative absolute error (%)
0	0.2	0.2	0	0	0
5	− 0.103118	− 0.110669	0.00755101	0.0732269	7.32269
10	0.0477275	0.0521663	0.0044388	0.0930029	9.30029
15	− 0.0138422	− 0.0157122	0.00187001	0.135095	13.5095
20	− 0.00164742	− 0.000968002	0.00067942	0.412414	41.2414
25	0.00422044	0.00396994	0.000250494	0.0593526	5.93526
30	− 0.00516406	− 0.00504564	0.000118419	0.0229313	2.29313
35	0.00662781	0.00655293	0.0000748789	0.0112977	1.12977
40	− 0.00378295	− 0.00373604	0.0000469122	0.012401	1.2401
45	− 0.0000212117	− 0.0000349296	0.0000137179	0.646712	64.6712
50	− 0.000914687	− 0.000930045	0.000015358	0.0167904	1.67904

In Fig. 3, the nonlinear ODE stated in Eq. (1) is indicated by the red curve. According to Eq. (8), the traditional solution is concurrently represented by a blue one. According to MS, the Absolute Error is estimated as 0.00777. Imagine a plane with two complicated trajectories: one representing the DSRO, a nonlinear system characterized by bio-stability and self-sustained oscillations, and the other a smooth path controlled by a linear oscillator. The nonlinear curve exhibits complex distortions and amplitude-dependent frequency fluctuations, whereas the linear curve follows a steady, harmonic motion. At first glance, their behavior appears to be quite different. But, the utilized approximation NPA brings these curves into alignment and captures essential features of nonlinear system in a useful linear model. In some regimes, this mapping allows linear system to serve as an accurate stand-in of the nonlinear system by preserving crucial dynamical features including frequency modulation and energy transfer. By connecting complexity with simplicity, the result enables a deep comprehension of nonlinear dynamics using well-known linear techniques.

For enhanced convenience, additional Tabular validation between Eqs. (5) and (8) are provided through Table 1.

### Comprehending error charts

#### Absolute error

The numerical difference between the estimated and actual values is shown by the absolute error. A lower absolute error indicates a more accurate estimate, indicating that the computation closely matches the real data.

#### Relative absolute error $(\boldsymbol{\%})$

The variance concerning the actual value and the approximated value divided by the actual value indicates the relative absolute error. The relative absolute error multiplied by 100 is quantified by the percentage relative error (%). This idea is fundamental to the experimental sciences, engineering, and physics, where specific quantities and approximations are necessary of analysis, strategy, and accuracy. While a small relative error indicates a very comparable approximation with little volatility, a big relative error indicates a significant departure from the real value, thus jeopardizing the accuracy and dependability of computations. It may be stated numerically as:

$${\mathrm{Relative}}\,{\mathrm{Error}}\,{\text{(\% )}} = \frac{{\left| {\text{Actual - Approximate}} \right|}}{{{\mathrm{Actual}}}} \times 100$$.

### Physical interpretation

An explanation for the small percentage error between the nonlinear system and its linear approximation regarding the ship’s rolling motion is as follows:

In moderate roll conditions, weak nonlinearities, such as cubic or higher-order restoring forces, do not significantly influence system behavior, permitting a linear model to effectively approximate motion with minimal errors. Likewise, when nonlinear damping mechanisms are weak or when primary energy-dissipation sources, such as bilge keels and wave radiation, operate nearly linearly within practical roll ranges, linear damping assumptions remain adequately precise. The nonlinear components of the restoring force typically remain minimal compared to the linear term, resulting in the system’s actual response closely aligning with linear predictions. Linearization is further validated when frequency-dependent nonlinear systems are aligned using energy-equivalent methods, which reduce discrepancies in the response spectra, rendering linear and nonlinear models nearly indistinguishable in practice.

These findings highlight how important numerical precision is, particularly in simulations where even little mistakes can add up over time to produce notable changes in system behavior. This emphasizes how important it is to use reliable numerical methods in domains like fluid dynamics, biomedical modeling, and engineering to guarantee long-term dependability and prediction accuracy. Furthermore, the dimensionless values shown in these Tables are essential in establishing the basic physical properties of the system. They guarantee that the outcomes are still relevant and scalable in a variety of real-world scenarios.

## Procedure in identifying an advanced linear ODE

To examine stability in non-resonance and resonance scenarios, a more thorough method is needed than the well-known primary solution in Eq. (15). The advantages of the proposed approach allow the trial solution to analyze vibrational activity in resonance response and offer new modeling tools. The importance of resonance properties in affecting a system’s behavior under external excitations is illustrated by this technique. This approach entails creating a complex, precise solution of Eq. (8). In order to examine if resonance exists in the system, this improved solution must be created. The starting point of this procedure is the linearized form of Eq. (8). This change in perspective is essential in a comprehensive study since it allows for a more understanding of dynamics of the system, particularly with relation to resonance occurrences. This work intends to offer profound insights into the oscillatory behavior of the system under many situations by examining advanced solutions, with a particular focus on important resonance and stability sites. Division by zero is rejected, indicating that the fraction’s denominator is still non-zero, according to an examination of response given in Eq. (15). Therefore, the absence of a resonance situation is indicated by Eq. (15). The development of this improved solution is necessary to examine whether resonance exists in the system. The first point for this procedure is the linearized form of Eq. (8). An analytical shift is necessary of a systematic investigation in order to have a thorough grasp of the system’s dynamics, especially in connection to resonance events. To get a thorough understanding of the oscillatory behavior of the system under different circumstances, this work investigates advanced solutions with an emphasis on important resonance and stability areas. The denominator of the fraction is still non-zero, as shown by the analysis of the solution given in Eq. (15), which shows that division by zero is overruled. The answer in Eq. (15) so indicates that there is no resonance situation. By transforming the nonlinear equation into a forced-damped linear oscillator, we apply an identical linearization procedure to examine the performance of the forced oscillator. The resonance events brought about by the action of external periodic force are not adequately considered by conventional methods’ standard accurate answer. To tackle this problem, a new method is proposed that allows of a more detailed analysis of resonance behavior and stability criteria. We give a more accurate analytical formulation using an enhanced trial solution on the relevant linearized problem. By using the Galerkin technique^45–49^, to determine unknown parameters, resonance phenomena and their impact on system stability may be thoroughly examined. The following sophisticated trial (guessing) response is the outcome of this:

16 $$\hat{u}(t) = (A - \gamma )\cos \psi t + \gamma \cos \sigma t$$

where $\psi ,\,\,\gamma ,\,\,{\mathrm{and}}\,\,A$ represent the forcing frequency, an unknown amplitude related to the periodic force, and the initial amplitude, respectively.

In Eq. (16), $\psi$ signifies global frequency, including the influences of the system’s intrinsic nonlinear properties and impact of external periodic force. Contrary to natural frequency $\omega_{0}$ or the equivalent frequency $\omega_{eqv}$, which principally distinguishes unforced or linearized response of oscillator, $\psi$ contains modifications convinced by periodic motivation. This parameter is crucial of accurately describing the oscillatory performance in both resonant and non-resonant conditions. The value is subsequently determined using the Galerkin method^45–49^, ensuring a precise depiction of the interaction between the system’s inherent dynamics and external driving force.

To augment our research and attain a supplementary understanding of resonance conduct in forced oscillator, we employ an advanced trial solution incorporating an unknown frequency $\psi$ and amplitude $\gamma$. This enhancement is crucial, as basic trial solution, meanwhile effective in non-resonant conditions, inadequately addresses the influence of the periodic forcing element in resonant scenarios. By modifying the proposed solution to include these additional features, we aim to achieve a further perfect representation of the system’s reaction to external excitation. To determine the values of $\psi$ and $\gamma$, an appropriate mathematical framework must be established. Owing to the complexity of nonlinear system, obtaining a direct analytical solution is challenging. We employ Galerkin method^45–49^, an efficient approximation technique that projects the governing equations onto a suitable function space, therefore ensuring optimal alignment of trial solution with system’s dynamics. This methodology allows of the systematic formulation of $\psi$ and $\gamma$, the necessary equations for resonance phenomena and stability characteristics in nonlinear oscillatory systems, hence permitting a more in-depth examination. Utilizing the Galerkin integral on the residual function produces the subsequent fundamental equations.

Substituting $\hat{u}(t)$ into the linear ODE as given in Eq. (8) and simplifying indicates the succeeding residual function:

17 $$R(\psi ,\,\gamma ;\,t) = (A - \gamma )\left( {\left( {\psi^{2} - \omega_{eqv}^{2} } \right)\cos \psi t + 2\mu_{eqv} \psi \sin \psi t} \right) + \left( {F + \left( {\sigma^{2} - \omega_{eqv}^{2} } \right)} \right)\cos \sigma t + 2\gamma \sigma \mu_{eqv} \sin \sigma t$$

To evaluate the unknown $\psi$ and $\gamma$, we use the Galerikn method^45–49^, which requires minimizing the residual over the domain solution, as follows:

First integral: Enforcing orthogonally for $\cos \psi t$, we have

18 $$\int\limits_{0}^{2\pi /\psi } {(A - \gamma )\left[ {\left( {\psi^{2} - \omega_{eqv}^{2} } \right)\cos^{2} \psi t + 2\mu_{eqv} \psi \sin^{2} \psi t} \right]} \,dt = 0$$

such that $A - \gamma \ne 0$, it follows that

19 $$\psi^{2} + 2\mu_{eqv} \psi - \omega_{eqv}^{2} = 0$$

Equation (19) results in a quadratic equation about the global frequency $\psi$, yielding two distinct roots $\psi_{1} \,\,\& \,\,\psi_{2}$. But as known the frequency must be positive, so just the positive root will be considered. This root is articulated as:

20 $$\psi = - \mu_{eqv} + \sqrt {\mu_{eqv}^{2} + \omega_{eqv}^{2} }$$

The second integral, pertaining to terms concerning $\cos \sigma t$ and $\sin \sigma t$, incorporates the influence of external periodic force, resulting in a vital equation for the unknown amplitude $\gamma$. The resultant Galerkin integral equation is presented as follows:

21 $$\int\limits_{0}^{2\pi /\psi } {\left[ {\left( {F + \gamma \left( {\sigma^{2} - \omega_{eqv}^{2} } \right)\cos^{2} \sigma t + 2\mu_{eqv} \sigma \sin^{2} \sigma t} \right)} \right]} dt = 0$$

To simplify the preceding integral, the excited frequency $\sigma$ is needed to be normalized as $\sigma = \alpha /T$, where $\alpha$ is some numerical value, and $T = 2\pi /\psi$.

The integral of Eq. (14) yields

22 $$\frac{\pi F}{\psi } + \gamma \alpha \mu_{eqv} + \frac{{\gamma \psi \alpha^{2} }}{4\pi } - \frac{{\pi \gamma \omega_{eqv}^{2} }}{\psi } + \frac{1}{2}\left( { - \gamma \mu_{eqv} + \frac{F\pi }{{\psi \alpha }} + \frac{\gamma \psi \alpha }{{4\pi }} - \frac{{\pi \gamma \omega_{eqv}^{2} }}{\psi \alpha }} \right)\sin 2\alpha = 0$$

By employing MS, when the global frequency $\psi$ between Eqs. (21) and (22) is eliminated, one finds

23 $$\eta_{2} \gamma^{2} + \eta_{1} \gamma + \eta_{0} = 0$$

where

24a $$\begin{gathered} \eta_{2} = \frac{1}{4}\omega_{eqv}^{2} \left[ \begin{gathered} 4\alpha^{2} \left( {2\pi \alpha \mu_{eqv}^{2} (\alpha - 2\pi )^{2} + \omega_{eqv}^{2} \left( {\alpha^{2} - 4\pi^{2} } \right)^{2} } \right) + \,4\alpha \omega_{eqv}^{2} \left( {\alpha^{2} - 4\pi^{2} } \right)^{2} \sin 2\alpha + \hfill \\ \,\,\,\,\,\,\,\,\,\,\,\,\,\,\,\,\,(\alpha + 2\pi )^{2} \left( { - 2\pi \alpha \mu_{eqv}^{2} + (\alpha - 2\pi )^{2} \omega_{eqv}^{2} } \right)\sin^{2} 2\alpha \hfill \\ \end{gathered} \right] \hfill \\ \,\,\,\,\,\,\,\, \hfill \\ \end{gathered}$$

24b $$\begin{gathered} \eta_{1} = - \pi^{2} (2\alpha + \sin 2\alpha )\left( { - 2\alpha (\alpha - 2\pi )\left( {\alpha \mu_{eqv}^{2} + 2(\alpha + 2\pi )\omega_{eqv}^{2} } \right)} \right)F + \hfill \\ \,\,\,\,\,\,\,\,\,\,\,\,\,\,\,\,\,\,\,\,\,\,\,\,(\alpha + 2\pi )\,\left( {4\pi \omega_{eqv}^{2} - \alpha \left( {2\omega_{eqv}^{2} + \mu_{eqv}^{2} } \right)\sin 2\alpha } \right) \hfill \\ \end{gathered}$$

and

24c $$\eta_{0} = 4\pi^{4} \left( {2\alpha + \sin 2\alpha } \right)^{2} F^{2}$$

As observed Eq. (23) yields a quadratic equation in the unknown amplitude $\gamma$, it produces two unique solutions $r_{1} \,\,\& \,\,r_{2}$, say. These solutions are presented as

25 $$r_{1,\,2} = \left( { - \eta_{1} \pm \sqrt {\eta_{1}^{2} - 4\eta_{0} \eta_{1} } } \right)/2\gamma_{2}$$

The advanced trial solution is now fully established, with its unknowns $\gamma_{j} (j = 1,\,2)$ and $\psi$ having a distinct genuine solution that is entirely assessed through the original solution as specified in the specifications $\mu_{eqv} \,\,\& \,\,\omega_{eqv}$ as given in Eqs. (9) and (10). Consequently, the advanced linear ODE can be expressed as follows:

26 $$\ddot{U} + 2\tilde{\mu }_{eqv} \dot{U} + \tilde{\omega }_{eqv}^{2} U = F\cos \sigma t$$

The evaluation of the characteristic parameters that involved in Eq. (26) is similar to that previously shown in Eq. (8), after applying the modified trial solution (16). Therefore, the required analysis for this occurrence is as shown in Eqs. (9) and (10). To follow the paper easily, the advanced damped parameter $\tilde{\mu }_{eqv}$ and the equivalent frequency $\tilde{\omega }_{eqv}$, for their too lengthy and complexity, are moved to some hyperlink (Parameters of Eq. (26).pdf). Consequently, the advanced linear solution shown in Eq. (26) yields an identical precise solution as depicted in Eq. (15), by substituting $\mu_{eqv} \to \tilde{\mu }_{eqv}$ and $\omega_{eqv} \to \tilde{\omega }_{eqv}$.

Therefore, subsequent study should concentrate on a comprehensive examination of the constraints of the suggested method. This includes the identification of parameter regimes, modeling assumptions, or operational conditions under which the approach may experience a decline in accuracy or fail to yield credible predictions. An in-depth examination of these boundaries would elucidate the applicability of the strategy and facilitate the advancement of enhanced or alternative solutions for scenarios that exceed its existing constraints.

The relationship between nonlinear and linear ODEs, as specified by Eqs. (1) and (26), can be numerically verified using the following figure and Table for improved clarity. For this purpose, and consider the following data selection:

$$\omega_{0} = 3.0,\,\,\mu = 0.1,\,\,F = 0.05,\,\,\alpha = 0.05,\,\,\gamma_{1} = 0.1,\,\gamma_{2} = 0.2\,\,\,,\,\,\delta_{1} = 0.4,\,\delta_{2} = 0.3,\,\delta_{3} = 0.2,\,\delta_{4} = 0.1,\,\,{\mathrm{and}}\,A = 0.2$$

**Fig. 4:**
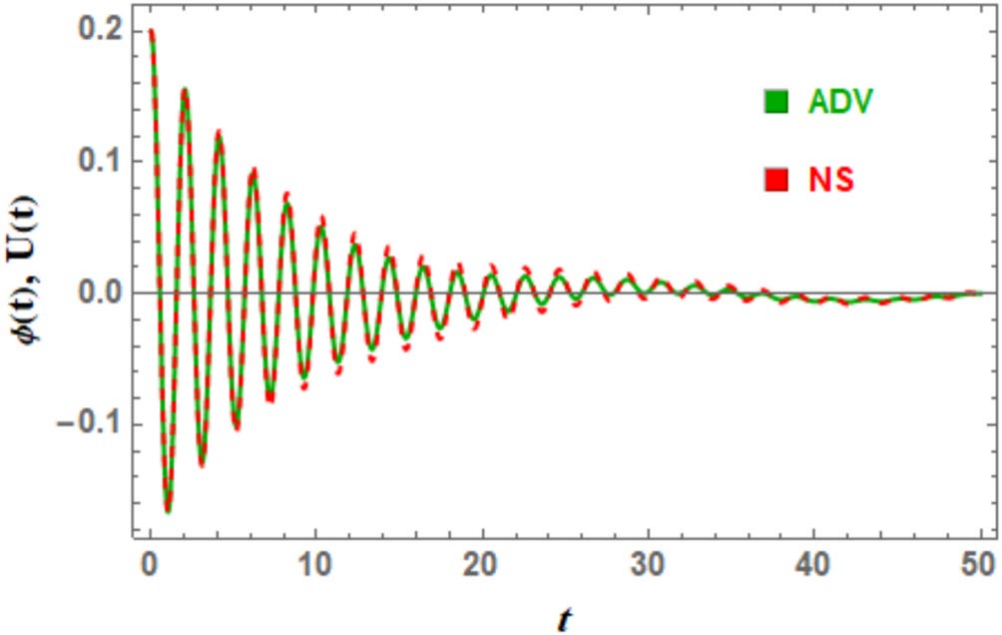
Illustrates a strong convergence among the solutions of nonlinear and linear ODEs as obtained by Eqs. (1) and (19).

The NS of DSRO, as provided by Eq. (1), and the advanced NPA solution, as provided by Eq. (26), are compared in Fig. 4 and Table 2. As can be seen, the figure shows a high degree of matching between the two solutions, with an estimated maximum error of 0.0059. One can notice that the error in this case, using the advanced trail solution (16), is less than the first one when trail solution is considered in Eq. (16). This outcome may be considered as an enhancement in NPA more than the previous literature^27–43^. As seen in Fig. 4, the two curves have the best correlation when their forms are almost the same. This implies that one curve may be effectively superimposed over the other by scaling, translation, and rotation. This connection can be interpreted in a number of ways:

**Table 2:** Presents a numerical comparison of Eqs. (1) and (26).

t	NS of the nonlinear Eq. (1)	NS of the advanced NPA Eq. 26	Absolute error	Relative absolute error	Relative absolute error (%)
0	0.2	0.2	0	0	0
5	− 0.0971513	− 0.0929275	0.00422382	0.0434768	4.34768
10	0.0414185	0.0356473	0.00577126	0.13934	13.934
15	− 0.0195691	− 0.0164479	0.00312118	0.159496	15.9496
20	− 0.00169614	− 0.00156325	0.000132892	0.0783498	7.83498
25	0.00936973	0.00718634	0.00218339	0.233026	23.3026
30	− 0.00104883	0.00175433	0.00280316	2.67265	267.265
35	0.00535094	0.00284964	0.00250131	0.467452	46.7452
40	− 0.00767451	− 0.00584803	0.00182648	0.237993	23.7993
45	− 0.00279337	− 0.00390287	0.00110949	0.397187	39.7187
50	− 0.000428967	0.000123265	0.000552232	1.28735	128.735

Two curves demonstrate a robust correlation when their geometric attributes, such as curvature, angle, and length distribution, are analogous along their paths, reflecting common local properties including tangent directions and curvature radii; this similarity can be quantified by assessing distances between corresponding points on the curves, with minimal cumulative separation, frequently analyzed through metrics such as the Hausdorff distance or point-to-point correspondence errors, indicating effective alignment. A high-quality match indicates that one curve can be transformed into another through translation, rotation, and occasionally scaling, demonstrating significant similarity. Additionally, structural characteristics such as symmetry, periodicity, or uniform patterns of inflection points must also correspond for the curves to align accurately.

### Differences between Tables 1 & 2

Tables 1 and 2 provide useful information about approximation procedure’s accuracy and error evolution over time. There are no absolute or relative mistakes at $t = 0$ since the real and estimated numbers are the same. This validates the initial accuracy of the approach. On the other hand, absolute and relative mistakes progressively increase with time. This shows buildup of numerical conflicts caused by approximation constraints, truncation mistakes, or numerical instability. This pattern demonstrates how little variations brought about by approximation approach might grow over time and have an impact on accuracy over the long run. The consistent increase in absolute and relative mistakes is a significant finding in both Tables. This illustrates how estimated and real answers gradually diverge. This implies that small differences are always present in numerical approximations and that these differences build up over time as a result of truncation effects or other computational limitations. The error measures at corresponding periods differ slightly numerically, even though both Tables show the same basic error increase pattern. This suggests that several parameter settings may exist. Interestingly, Table 2 shows somewhat fewer mistakes than Table 1. This might point to a system with slightly changed physical characteristics or a more sophisticated approximation technique, which would increase numerical accuracy.

## Stability configuration in absence of external force

As an extension of NPA, the objective now is to scrutinize the stability investigation of the provided advanced scenario without external force ($F = 0$) because it is challenging to do so when this force is present. Another approach is required to examine the stability illustration in the presence of external force. Using the formula provided in Eq. (26) at $F = 0$, stability graphs of comparative construction are displayed for convenience. The impact of this strategy is confirmed by Table 2 and Fig. 4. In the nonexistence of exterior force Eq. (26) could be transformed to another form by using the normalized form:

27 $$U = e^{{ - \tilde{\mu }_{eqv.} t}} V(t)$$

herein $V(t)$ is required periodic function to be evaluated.

One may obtain the differential formulation that governs the unknown function $V(t)$ by substituting Eq. (27) into Eq. (26):

28 $$\ddot{V} + \chi^{2} V = 0$$

which is the normal simple harmonic equation with the frequency $\chi$ that is specified as.

29 $$\chi^{2} = \tilde{\omega }_{eqv}^{2} - \tilde{\mu }_{eqv}^{2}$$

where, as previously said, the advanced damped parameter $\tilde{\mu }_{eqv}$ and the comparable frequency $\tilde{\omega }_{eqv}$ have been known from the context. Relative to prior techniques, the new methodology stands out as an intriguing, uncomplicated, and powerful approach in investigating nonlinear stability, the primary aim of this model. With this novel approach, a wide range of nonlinear formulas may be explored. The stability circumstance can now be expected to take the following forms:

30 $$\chi^{2} > 0,\,and\,\tilde{\mu }_{eqv} > 0$$

**Fig. 5:**
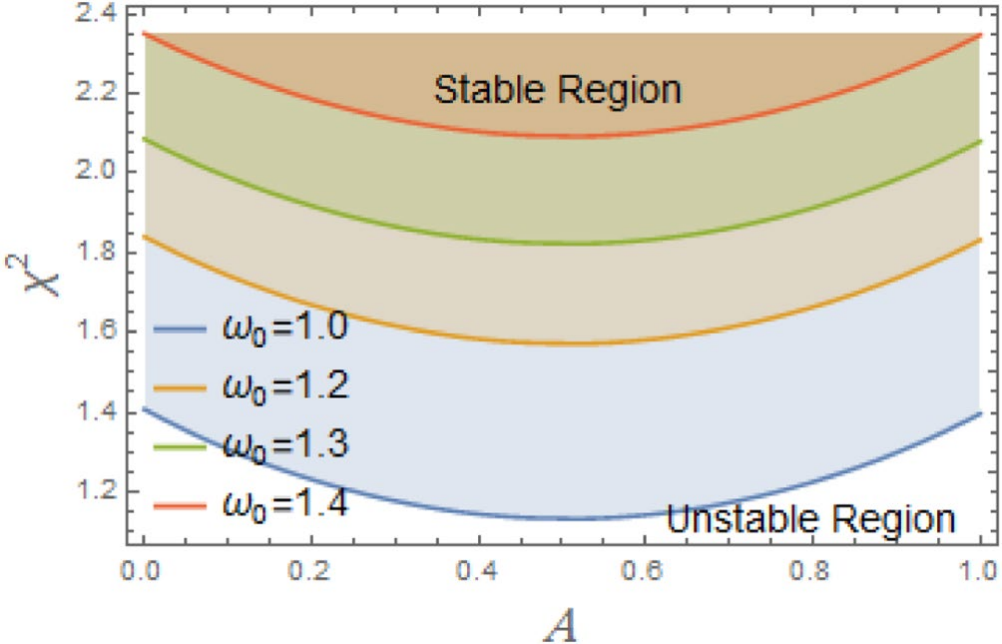
Displays the stability zones corresponding to different values of natural frequency $\omega_{0}$.

**Fig. 6:**
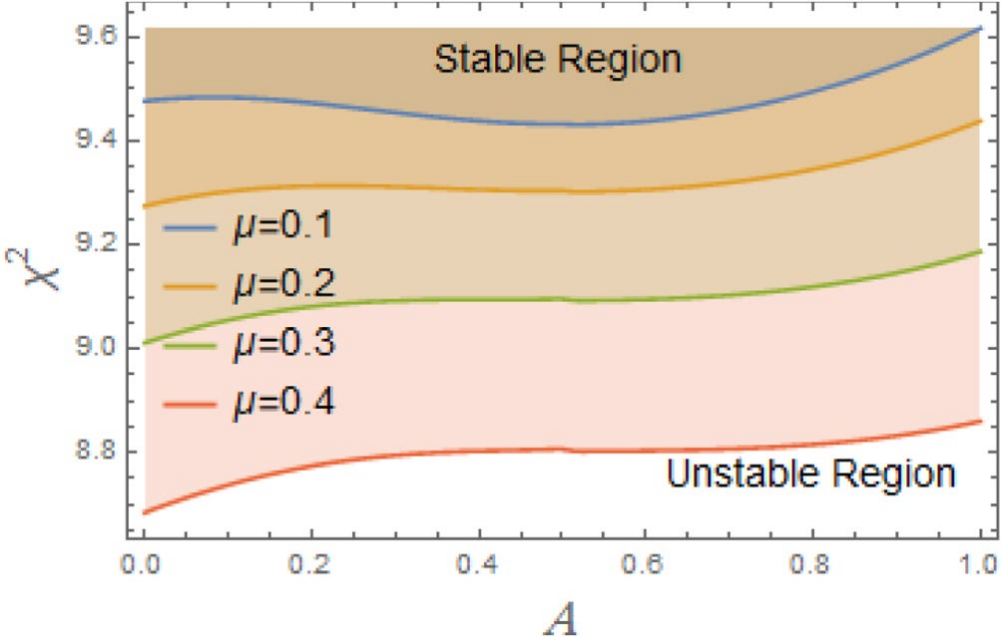
Displays the effect of the linear damping element $\mu$ on the stability regions.

**Fig. 7:**
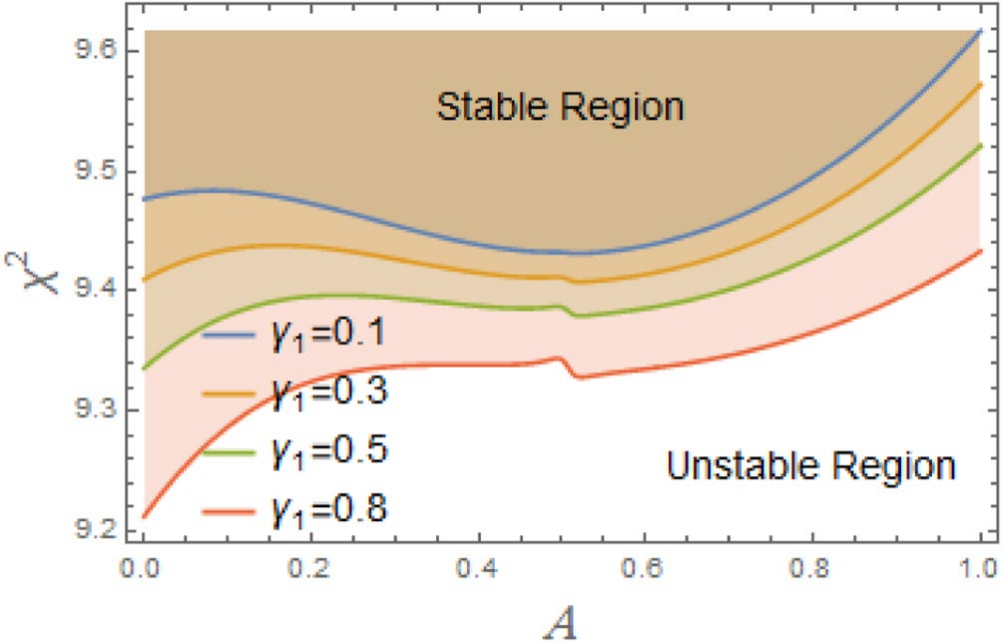
Displays the variation of stability regions with respect to $\gamma_{1}$.

**Fig. 8:**
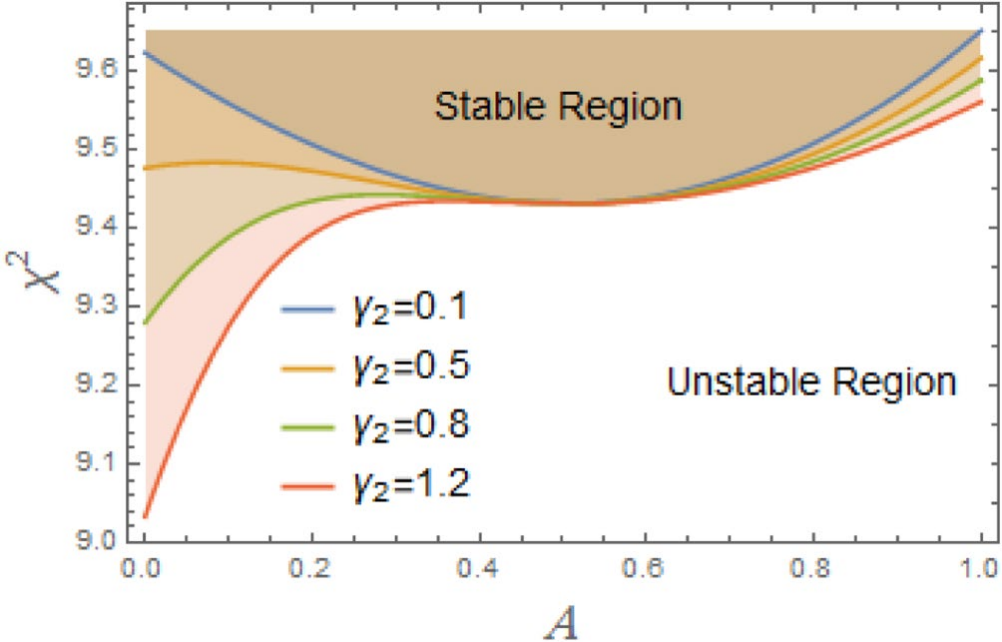
Displays the stability zones corresponding to different values of $\gamma_{2}$.

**Fig. 9:**
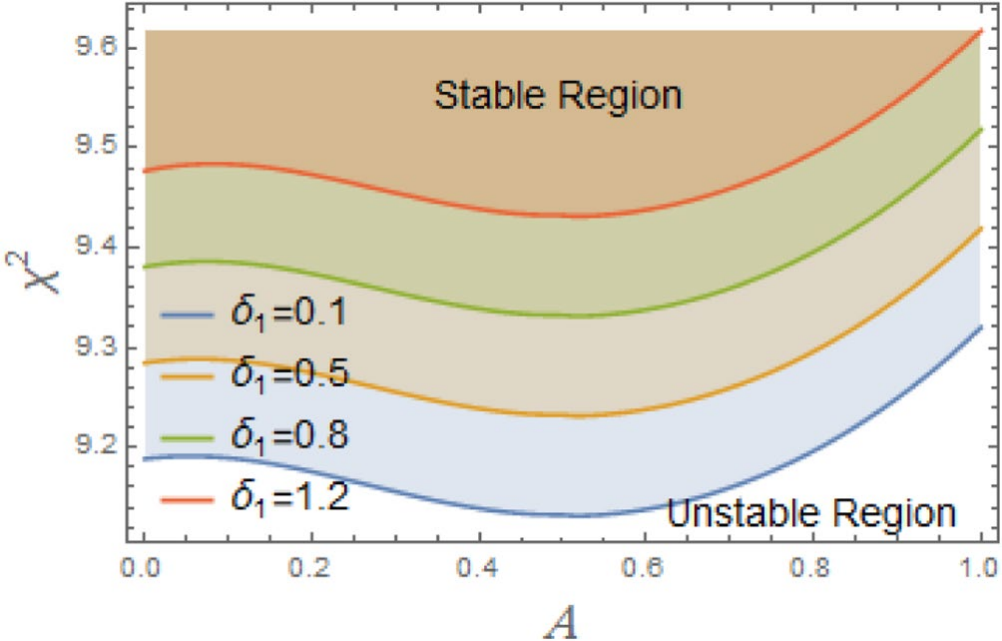
Displays the stability areas concerning to the factor $\delta_{1}$.

**Fig. 10:**
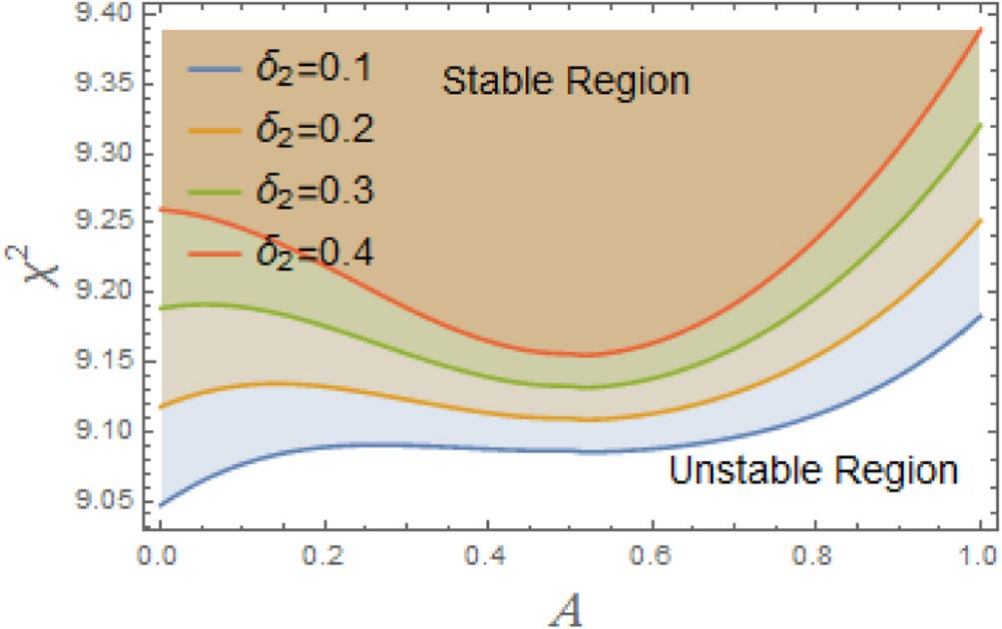
Shows the stability regions concerning to the factor $\delta_{2}$.

**Fig. 11:**
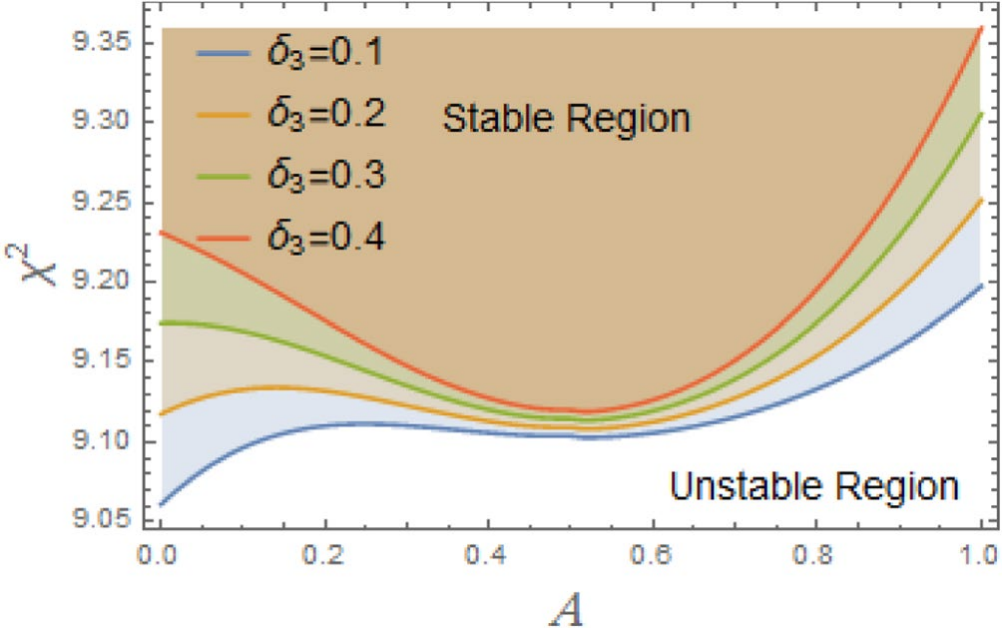
Shows the stability areas concerning to the factor $\delta_{3}$.

**Fig. 12:**
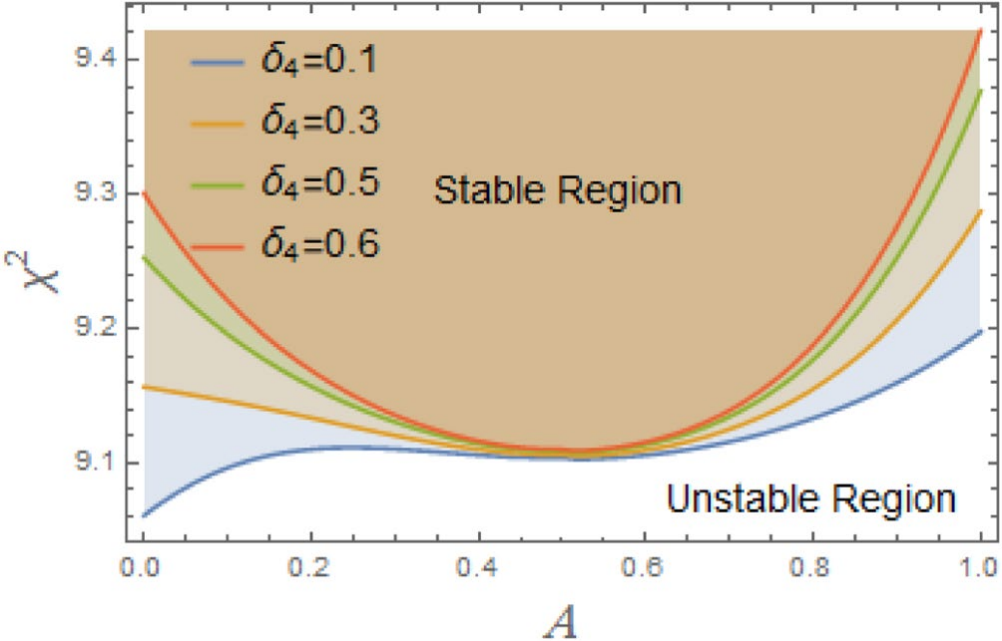
Displays the stability zones concerning to the factor $\delta_{4}$.

**Fig. 13:**
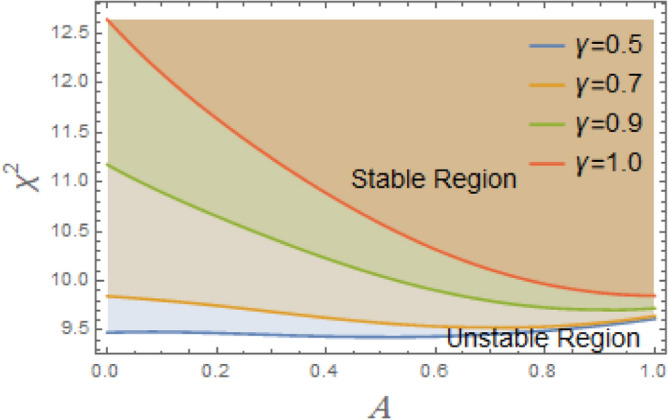
Displays the stability zones concerning to the factor $\gamma$.

**Fig. 14:**
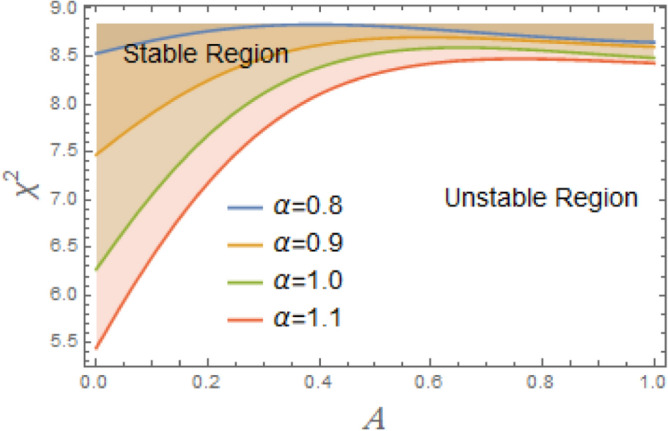
Displays the stability regions concerning to the factor $\alpha$.

The constraint $\tilde{\mu }_{eqv} > 0$ should be certified to be possible to continue the current procedure, which is verified by using MS. Subsequently, Figs. 5, 6, 7, 8, 9, 10, 11, 12, 13, 14 are plotted to examine the stability pattern utilizing constraint $\chi^{2} > 0$, where $\chi^{2}$ is configured against the primary amplitude $\,A$. The colored area above each curve indicates stability region, while uncolored area under curves specifies destabilizing zone. It is noted that the circumstance $\chi^{2} > 0$ is detected as a transcendent inequality of parameters $\mu ,\delta_{1} ,\delta_{2} ,\delta_{3} ,\delta_{4} ,\lambda_{1} ,\lambda_{2} ,\omega_{0} ,\gamma ,\alpha ,\,\,$ and $\,A$. As noted in these figures consuming the same previous data created Fig. 4. Because of the parameter under consideration, this data varies from one figure to another. These figures show the stable zones of a field with respect to the ICs. All transition curves conform to Eq. (29). Consequently, all locations above these transition curves satisfy $\chi^{2} > \tilde{\omega }_{eqv}^{2} - \tilde{\mu }_{eqv}^{2}$. This indicates that the area above the transition curves generates stable zones. Conversely, all points beneath these transition curves result in unstable zones, where $\chi^{2} < \tilde{\omega }_{eqv}^{2} - \tilde{\mu }_{eqv}^{2}$.

Figure 5 illustrates how the natural frequency $\omega_{0}$ affects stable zones. It is shown that stable zone significantly decreases with elevation. Consequently, the increase $\omega_{0}$ has a noticeable destabilizing effect, albeit at somewhat different intensities. This frequency thus creates a spooky impression. A structure’s intrinsic frequency dictates how rapidly it oscillates in response to outside stimuli. Engineers must consider a structure’s inherent frequency while constructing it in order to avoid problems like wear, failure, and resonance. Without external damping or stimulation, a system’s intrinsic frequency, or its inherent oscillation rate, can become unstable. Resonance can result in high-amplitude oscillations when an outside force or disturbance gets close to system’s fundamental frequency. The system may become unstable or collapse if these oscillations are beyond its structural bounds. By acting as a threshold, natural frequency efficiently prevents minor disruptions from causing significant and potentially dangerous alterations. It is thought to be a key component in figuring out if energy input matches system’s oscillatory nature. The intricacy of forcing, damping, and nonlinearities determines whether this alignment leads to controlled oscillations or destabilizing outcomes like resonance, bifurcations, or chaotic dynamics. Although a vessel with a greater natural frequency is more rigid and hence less prone to roll, it also reacts to wave excitations more quickly. Phase mismatches, energy pumping into unstable modes, and resonance effects can all be exacerbated by this quicker reaction in nonlinear systems, particularly those with nonlinear damping and restoring moments. Accordingly, stable areas in phase space are smaller, and chaotic or divergent motion becomes more likely.

According to Fig. 6, when the linear damping coefficient $\mu$ increases, stable zones expand considerably. It is discovered that stability areas are expanding significantly when $\mu$ grow within a narrow range, between 0.1 and 0.4. This demonstrates that, as is physically true, $\mu$ has a stabilizing effect on system under discussion. Through energy dissipation, resonance effect reduction, and nonlinear instability suppression, linear damping coefficient contributes to stabilization. It is crucial for maintaining system stability, especially in the presence of strong nonlinearities or outside inputs. The understanding of stabilizing influence of $\mu$ on nonlinear systems like DSRO could be interpreted for many reasons. The linear damping removes system’s energy to counteract energy intake from external forcing or nonlinear self-excitation. By doing this, the motion is stabilized and oscillation amplitude is prevented from increasing indefinitely. Damping reduces system’s reactivity at or near its inherent frequency. The peak amplitude at resonance falls with increasing dampening. By doing this, resonance amplification’s destabilizing effects are lessened. In systems with nonlinear terms. Damping reduces impact of nonlinear bifurcations, such as the formation of limit cycles or chaotic transitions. It shifts the critical thresholds of instability to higher quantities of parameter change or external force. Damping stabilizes stationary sites by accelerating the rate at which disturbances subside. According to linear stability analysis, more damping produces eigenvalues with more negative real parts, which ensures faster convergence to equilibrium. More damping reduces sensitivity to initial conditions and the complexity of phase space trajectories in chaotic regimes. This can lead to the restoration of periodic or quasi-periodic motion.

To comprehend the influence of damping force on the present organization, Figs. 7 and 8 are designed to demonstrate the effects of the impure cubic damping coefficient $\gamma_{1}$ and the pure cubic damping coefficient $\gamma_{2}$ on stability structure. It is found, similar to the influence of the linear damping factor $\mu$, that the growth of these parameters $\gamma_{1}$ and $\gamma_{2}$ enhances the stability area. The nonlinear impure cubic damping term, as denoted by $\gamma_{1} \varphi^{2} \dot{\varphi }$, in context of ship roll motion, describes a damping effect that contributes from a variety of sources, including hydrodynamic forces, whirlpool formation, and wave radiation, rather than being entirely linear or viscous. It is important to understand how roll damping preserves stability of ship, as seen in Fig. 7. Through a variety of processes, including wave radiation, vortex shedding, and hull-water interactions, the ship’s capacity to dissipate roll energy is represented by rolling damping coefficient. Stability is improved over time when roll oscillations’ amplitude is decreased by increased damping. Unlike pure viscous damping, which has an honest relation to velocity, impure damping has nonlinear effects, meaning that damping changes as the frequency and roll amplitude change. Because of intricate water-hull interactions, resistance rises disproportionately at large roll amplitudes. After a disturbance, a ship can restore to equilibrium faster with a greater impure damping coefficient. However, too little damping produces protracted oscillations, while too much damping might produce slow responses. The impure damping coefficient affects how well stabilizers (such as bilge keels, fins, and anti-roll tanks) work since they interact with the ship’s inherent damping properties. For the best roll reduction, damping components must be properly adjusted.

Moreover, the nonlinear pure cubic damping coefficient in a system, denoted by $\gamma_{2} \dot{\varphi }^{3}$, also has a stabilizing effect, as noted in Fig. 8. In ship roll motion, pure nonlinear damping coefficient characterizes a damping force that has a nonlinear connection rather than being proportional to velocity in a straightforward linear fashion. The ship’s overall stability and reaction to external disturbances are influenced by this nonlinear damping, which is essential of stabilizing ship roll since it dissipates energy more efficiently at larger roll amplitudes. Nonlinear damping has little effect when a ship rolls at short angles, enabling regular operational movement. Nonlinear damping forces dramatically rise at extreme roll angles, efficiently dissipating energy and avoiding excessive rolling that would cause capsizing. Resonance may develop in response to periodic wave excitations if roll damping were just linear. Extreme rolling is less likely when nonlinear damping is used because it introduces energy dissipation processes that offset resonant amplification. Unlike linear damping, which simply shifts the decay rate of oscillations, nonlinear damping alters effective natural roll period by changing ship’s dynamic response depending on roll amplitude. Nonlinear damping is necessary for bilge keels and anti-roll tanks to function at their best. Stabilizing devices work better when they’re most needed because of the increased energy dissipation at larger amplitudes.

The configuration of system stability with variation of linear and nonlinear restoring force coefficients $\delta_{1} ,\,\delta_{2} ,\delta_{3}$, and $\delta_{4}$ is intended to be demonstrated in Figs. 9, 10, 11, 12. As illustrated in Fig. 9, the system becomes destabilized due to interaction between the periodic forcing term and the linear coefficient $\delta_{1}$ in the nonlinear DSRO. This leads to parametric instability areas, which, when joint with the nonlinearities of the system, cause complicated dynamic behavior and exponential development in oscillations. The added frequency coefficient’s destabilizing effect is crucial in ship rolling dynamics, especially when external wave stimulation takes place close to the system’s frequency. Strongly associated with resonance, this phenomenon may lead to excessive roll motions, thus jeopardizing the stability of ship. The coefficient of cubic component $\delta_{2}$ additionally destabilizes system by changing the stability limits in the parameter space, adding amplitude-dependent behavior, and perhaps causing chaotic dynamics, as seen in Fig. 10. The third-order nonlinear coefficient has a significant effect on ship rolling dynamics and stability. The ship may become unstable at times due to the addition of sub-harmonic resonance, the probability of chaotic behavior, and amplitude-dependent stiffness. To lessen these disruptive impacts, effective operational and design methods are vital.

As clarified in Fig. 11, the fifth term’s coefficient $\delta_{3}$ serves as an instability factor, intensifying the system’s innate instabilities and producing intricate, sometimes chaotic, dynamic behavior. The restoring moment’s nonlinear fifth-order component makes ship’s rolling dynamics more complicated, especially at large roll degrees. Because it increases the probability of chaos, modifies resonance behavior, and intensifies nonlinearities, this statement has destabilizing repercussions. Its impact rises as roll degrees rise, necessitating careful design and operational strategies to maintain stability, even if it is less obvious at lower angles. Moreover, as obtained from Fig. 12, the restoring force coefficient of the seventh degree $\delta_{4}$ also has a destabilizing influence on the current system. The restoring force is in charge of returning a ship to its equilibrium position following a roll disturbance, according to ship stability studies. In order to account of large-angle stability issues, this restoring force is frequently represented as a nonlinear function of the roll angle $\varphi$, integrating higher-order elements. Some of these terms can have destabilizing consequences when restoring force coefficients up to the seventh degree, which can result in severe rolling behavior, including capsizing, parametric rolling, or loss of stability. Although the purpose of restoring forces is to stabilize ship roll, excessive rolling, parametric resonance, and even capsizing can result from incorrectly adjusted nonlinear and linear restoring force coefficients up to seventh degree. By ensuring that these coefficients positively contribute to stability, proper ship design helps to avoid harmful rolling tendencies in rough sea conditions.

Figure 13 illustrates how the system responds to changes in advanced amplitude $\,\gamma$. It is found that stability region shrinks with the rise of $\,\gamma$ as seen in Fig. 13. The oscillatory motion of a ship in response to external excitations, such as waves, is referred to as a periodic solution in ship roll dynamics. The scale of oscillations is determined by periodic solution’s amplitude, and in some cases, the excessive amplitude can have destabilizing consequences that expose ship stability and perhaps cause capsizes. The ship may enter an unstable area where restoring moment is insufficient if roll amplitude increases over a particular threshold. Capsizing may result from this, particularly if ship experiences more disruptions. Parametric rolling may happen if the wave frequency is double the ship’s natural roll frequency or almost so. Extreme roll angles that have the potential to destabilize the vessel result from the exponential growth in the amplitude of periodic oscillations under these conditions. The ship’s capacity to right itself may be diminished at extreme roll degrees by nonlinear restoring force factors, such as cubic and quintic components. These nonlinearities may produce many equilibrium locations rather than stabilizing the motion, which might result in unexpected and potentially risky behavior. At high amplitudes, damping efficacy may reduce, causing oscillations to continue longer and increasing the risk of instability.

Furthermore, Fig. 14 shows the influence of the advanced frequency $\,\alpha$ on the stability zone. It can be seen from this figure that stability region enlarges with the rise of $\,\alpha$. The ship does not have enough time to react completely too external force when ship’s natural roll frequency is much smaller than excitation one. Limiting roll amplitude lowers chance of capsizing and avoids excessive motion. Resonant rolling, in which the stimulation frequency coincides with natural roll frequency, is one of most hazardous situations of a ship. Resonant amplification is prevented when the frequency enters advanced range, guaranteeing safer mobility. Many roll-damping systems, such as active stabilizers and bilge keels, function better at higher frequencies when resistance forces rise with speed. This also lowers the chance of excessive rolling by improving energy dissipation. In higher-frequency wave circumstances, waves with shorter wavelengths pass through ship’s hull, imparting less rolling energy than waves with long periods that correspond to roll frequency of ship. Large rolling motions are less likely as a result.

Consequently, future study must include comprehensive numerical calculations to validate the expected stability limitations determined in analogous issues. Time-domain simulations and parametric sweeps of relevant system parameters specifically validate the accuracy and dependability of the analytical results. These numerical calculations will aid in quantifying the effects of modeling assumptions and nonlinearities, hence increasing confidence in the proposed stability criteria and guiding their application to practical systems.

### Time history analysis

**Fig. 15:**
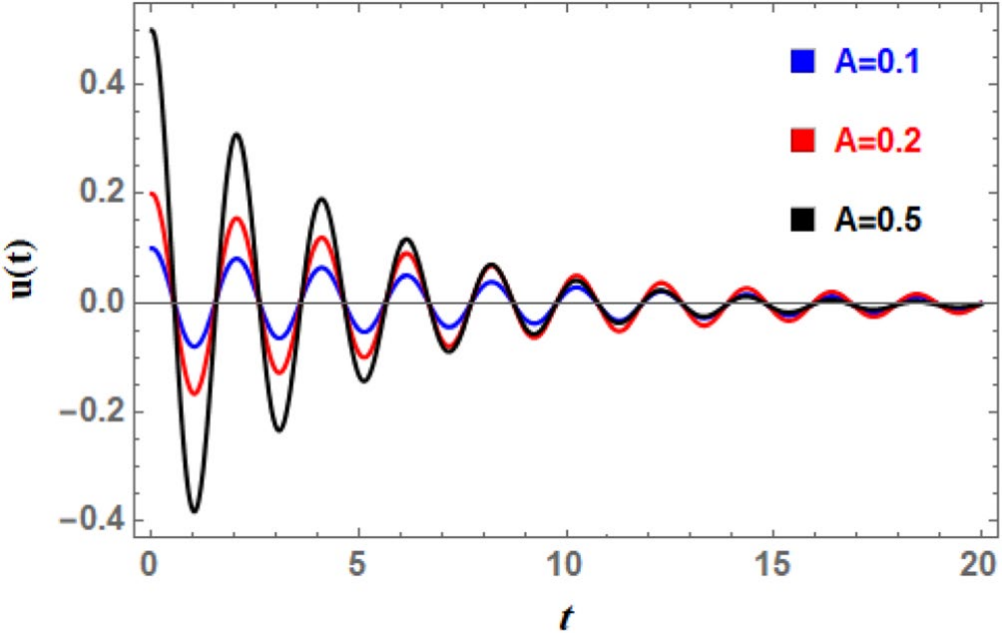
Indicates the influence of the initial amplitude $A$ on the time-history of $u(t)$.

**Fig. 16:**
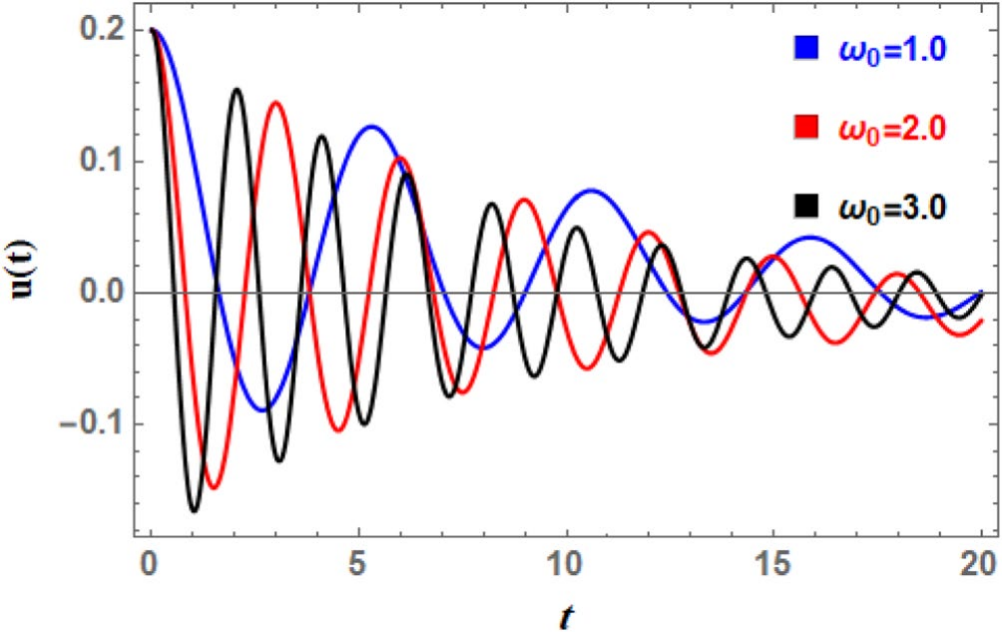
Indicates the influence of the natural frequency $\omega_{0}$ on the time-history $u(t)$.

**Fig. 17:**
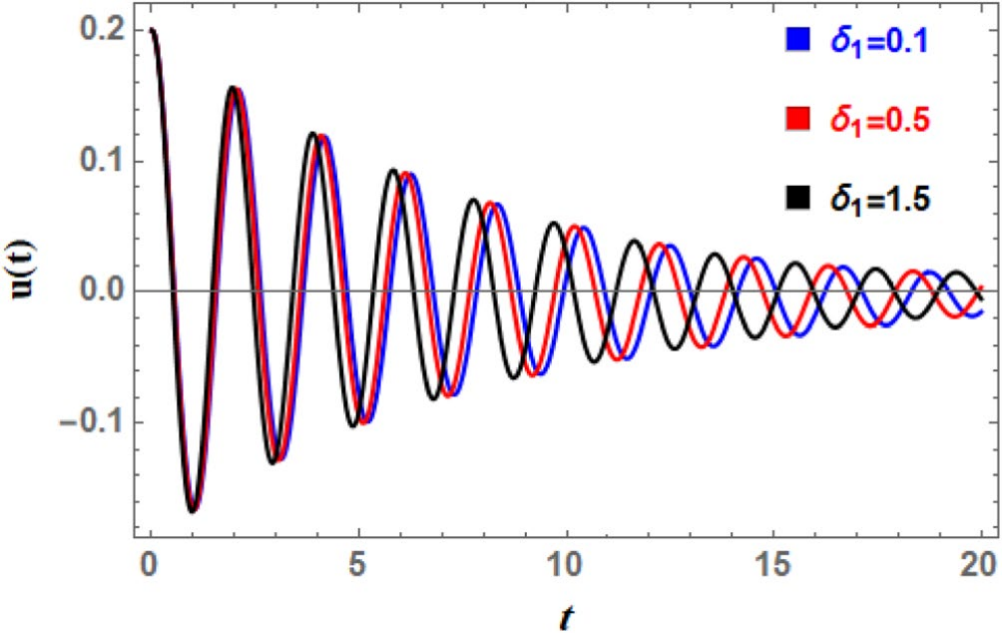
Indicates the influence of the additional frequency $\delta_{1}$ on the time-history $u(t)$.

**Fig. 18:**
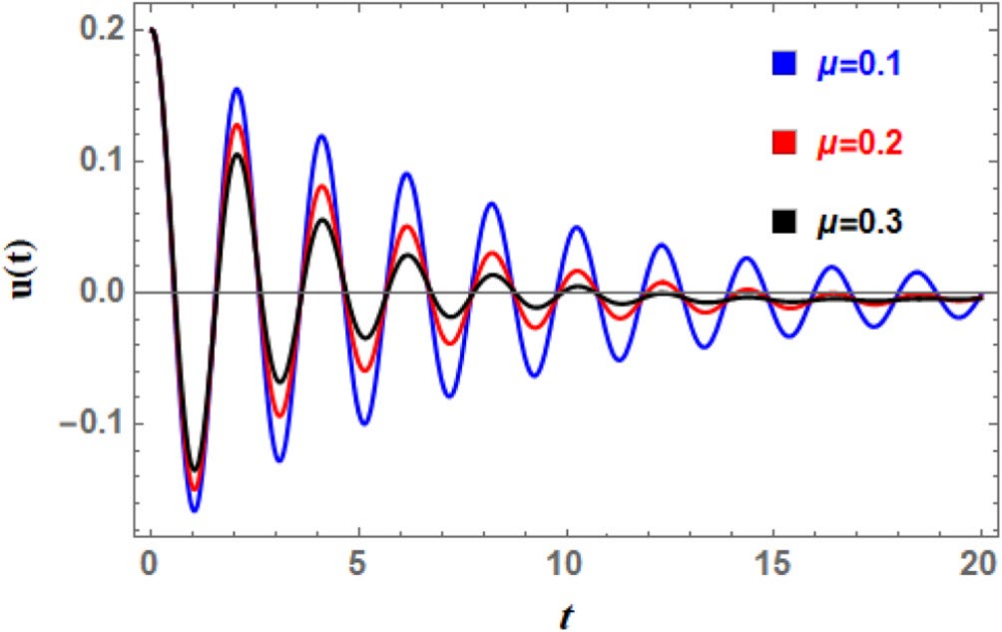
Indicates the influence of the linear damping $\mu$ on the time-history $u(t)$.

**Fig. 19:**
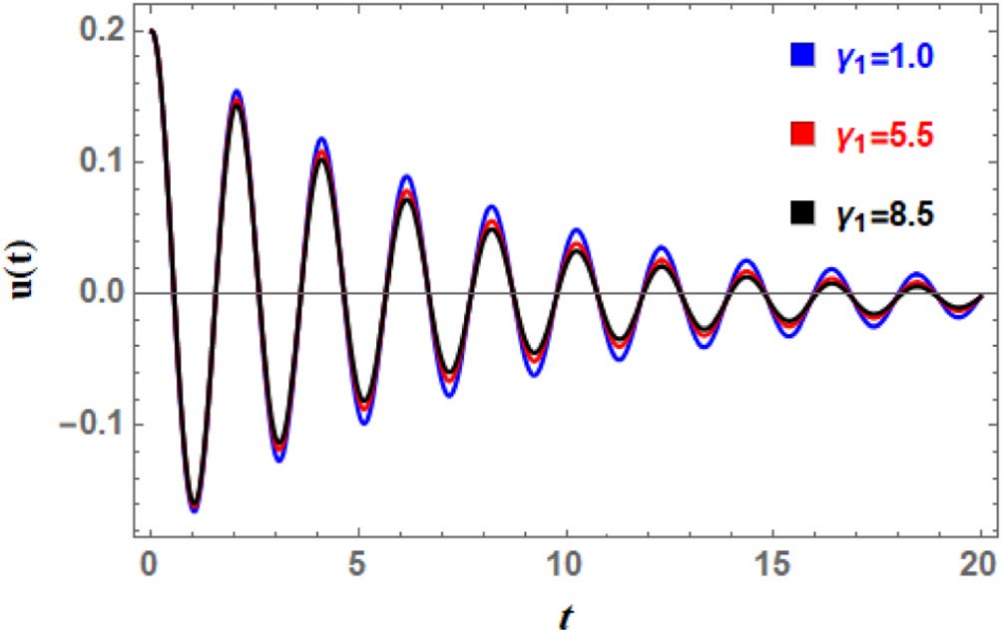
Indicates the influence of the impure cubic damping coefficient $\gamma_{1}$ on the time-history $u(t)$.

**Fig. 20:**
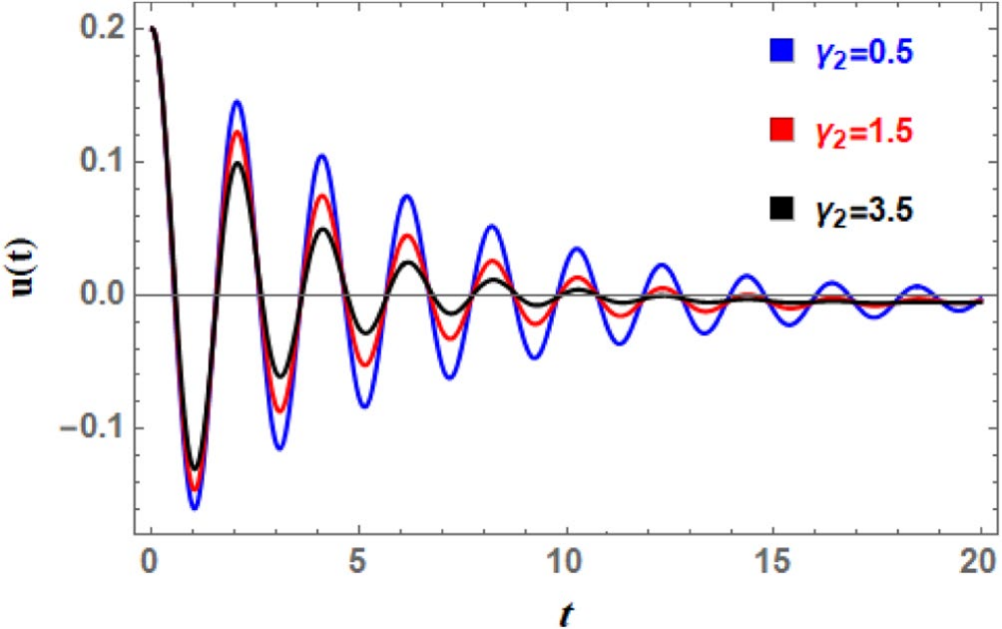
Indicates the influence of the pure cubic damping coefficient $\gamma_{2}$ on the time-history $u(t)$.

Using the advanced NPA, the damped DSRO solutions as stated in Eq. (26) are specified versus time graphically (time historical graphs) in Figs. 15, 16, 17, 18, 19, 20 using the following data:

$$\omega_{0} = 3.0,\,\,\mu = 0.1,\,\,F = 0.05,\,\,\alpha = 0.05,\,\,\gamma_{1} = 0.1,\,\gamma_{2} = 0.2\,\,\,,\,\,\delta_{1} = 0.4,\,\delta_{2} = 0.3,\,\delta_{3} = 0.2,\,\delta_{4} = 0.1,\,\,{\mathrm{and}}\,A = 0.2$$,

which vary depending on the displayed parameter in each illustration. All of these data make it evident that the oscillations are becoming less pronounced over time, and that the wave amplitudes are continuously decreasing with time when looking at time history. This makes sense in light of damping force.

Figure 15 reveals the effect of the beginning amplitude $A$. As seen from this figure, it is evident that as $A$ increases, so do the amplitudes of the damped waves. As $A$ increases, neither the number of vibrations nor the wavelengths of $U(t)$ can be altered. Because $A$ is increasing, waves are still sent regularly. In a damped system, the periodic solution’s amplitude determines the oscillation’s starting energy. The decline of higher initial amplitudes is slower because damping has to release more stored energy. Combining damping with large amplitudes can change decay rates by causing oscillation frequency to diverge from natural frequency. Oscillations in early time history are more noticeable with higher periodic solution amplitudes. When force is provided, oscillations ultimately settle into a periodic solution with a reduced amplitude; when damping is not applied, oscillations eventually vanish entirely.

Figure 16 illustrates the impact of the natural frequency $\omega_{0}$ on time history. This figure reveals that when $\omega_{0}$ increases, the number of pulsations increases dramatically and wave amplitudes rise somewhat. Furthermore, as natural frequency rises, wavelengths decrease. The waves also accelerate as $\omega_{0}$ increases; this rushing is logical since the system’s intrinsic oscillatory activity is described by the aggregate wave frequency. Given the nature of $\omega_{0}$, all of the aforementioned statements are inherently true. Nonlinear processes cause apparent frequency in time history to fluctuate in amplitude, and interactions between natural frequency and nonlinear components (such as cubic or quintic stiffness) in nonlinear systems lead to complex modulations and frequency shifts. Figure 17 shows how the linear coefficient affects the DSRO ‘s temporal history. It can be observed from Fig. 17 that when $\delta_{1}$ increases, the wave amplitudes, wavelengths, and oscillation count all remain about constant. As the wave rises, a slight decay in the overall wave is obtained. The linear coefficient $\delta_{1}$ in ship rolling Eq. (5) represents the linear restoring force that becomes significant even at small roll degrees. The destabilizing effect of additional frequency coefficient is a critical aspect of ship rolling dynamics, particularly when external wave stimulation occurs near system frequency. The ship’s stability may be in danger due to this phenomenon, which is strongly linked to resonance and can cause extreme roll motions.

Furthermore, the effect of linear damping factor $\mu$ on the temporal distribution of $U(t)$ is seen in Fig. 18. This graphic shows that, as $\mu$ rises, the wave’s fading increases as well, which makes sense. Furthermore, as $\mu$ grows, the amplitudes rise whereas the frequency of oscillations and the wavelengths of $U(t)$ remain the same. Enhancing the damping coefficient reduces resonance by reducing oscillatory energy transfer from external stimuli; it also increases stability by reducing oscillation amplitude and encourages energy dissipation, which speeds up the weakening of roll motion. Additionally, the roll amplitude decreases when $\mu$ increases with time, hastening the achievement of the steadiness state.

A nonlinear damping impact on ship’s rolling motion results from the use of energy dissipation methods that are velocity-dependent and become significant at high roll velocities. Roll dynamics calculations provide different results from linear damping coefficients, especially for large amplitude movements. Consequently, Figs. 19 and 20 are used to display the effects of the impure cubic damping coefficient $\gamma_{1}$ and the pure cubic damping coefficient $\gamma_{2}$, respectively. These two figures indicate that while the number of fluctuations and wavelengths stay unchanged, the oscillations’ amplitudes have somewhat risen. As $\gamma_{1}$ and $\gamma_{2}$ grows, there is an increase in wave decay, although the ratio is smaller than when $\mu$ rises. This suggests that primary factor causing roll movement’s dampening is the linear damping parameter, in addition to nonlinear damping coefficients. To stabilize the system versus the stimulating fluctuations caused by outside forces, the powerful nonlinear damping lessens the amplitude of massive roll motions and absorbs a significant amount of energy at higher roll velocities.

### Phase plane arrangement

**Fig. 21:**
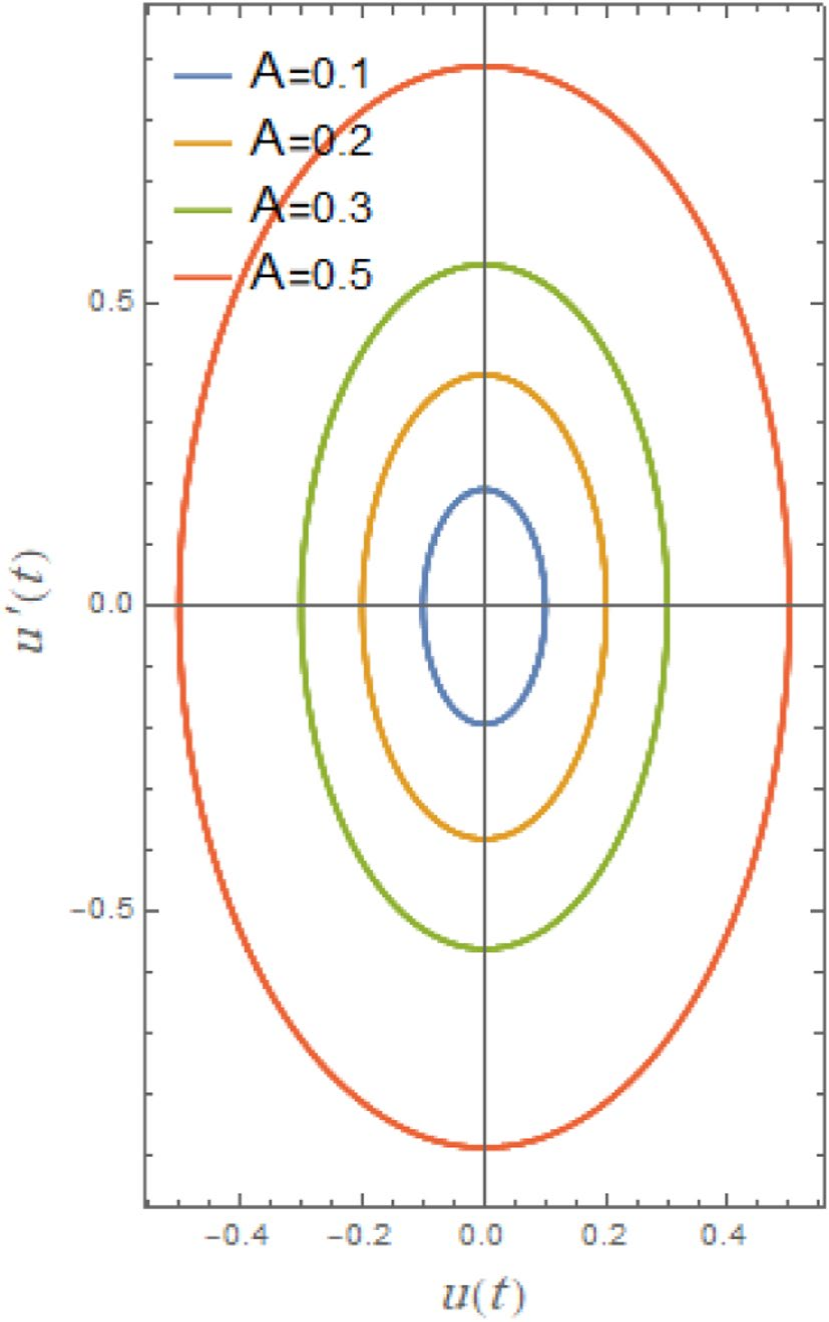
Indicates the influence of $A$ on the phase plane $uu^{\prime}$ of $u(t)$.

**Fig. 22:**
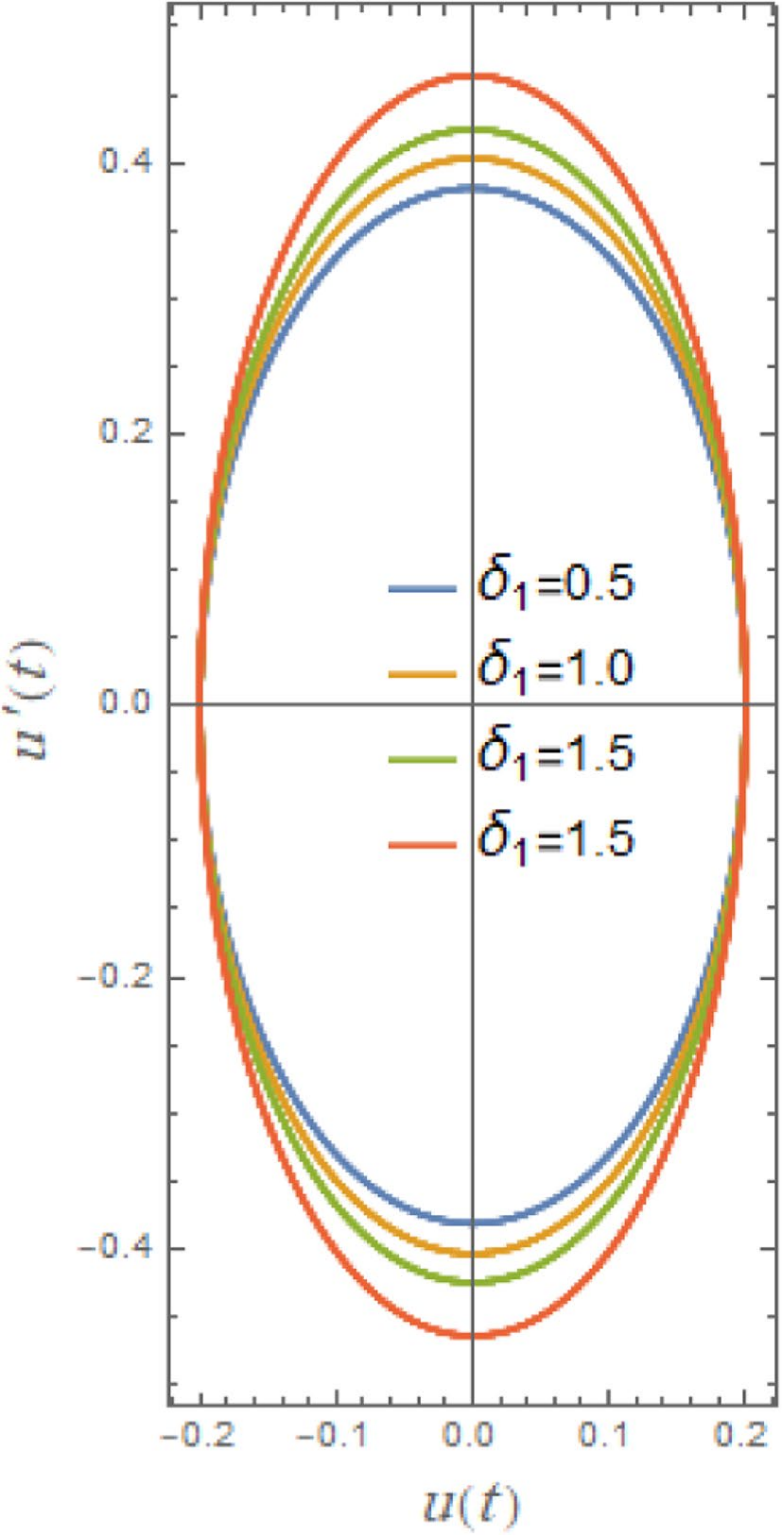
Indicates the influence of $\delta_{1}$ on the phase plane $uu^{\prime}$ of $u(t)$.

**Fig. 23:**
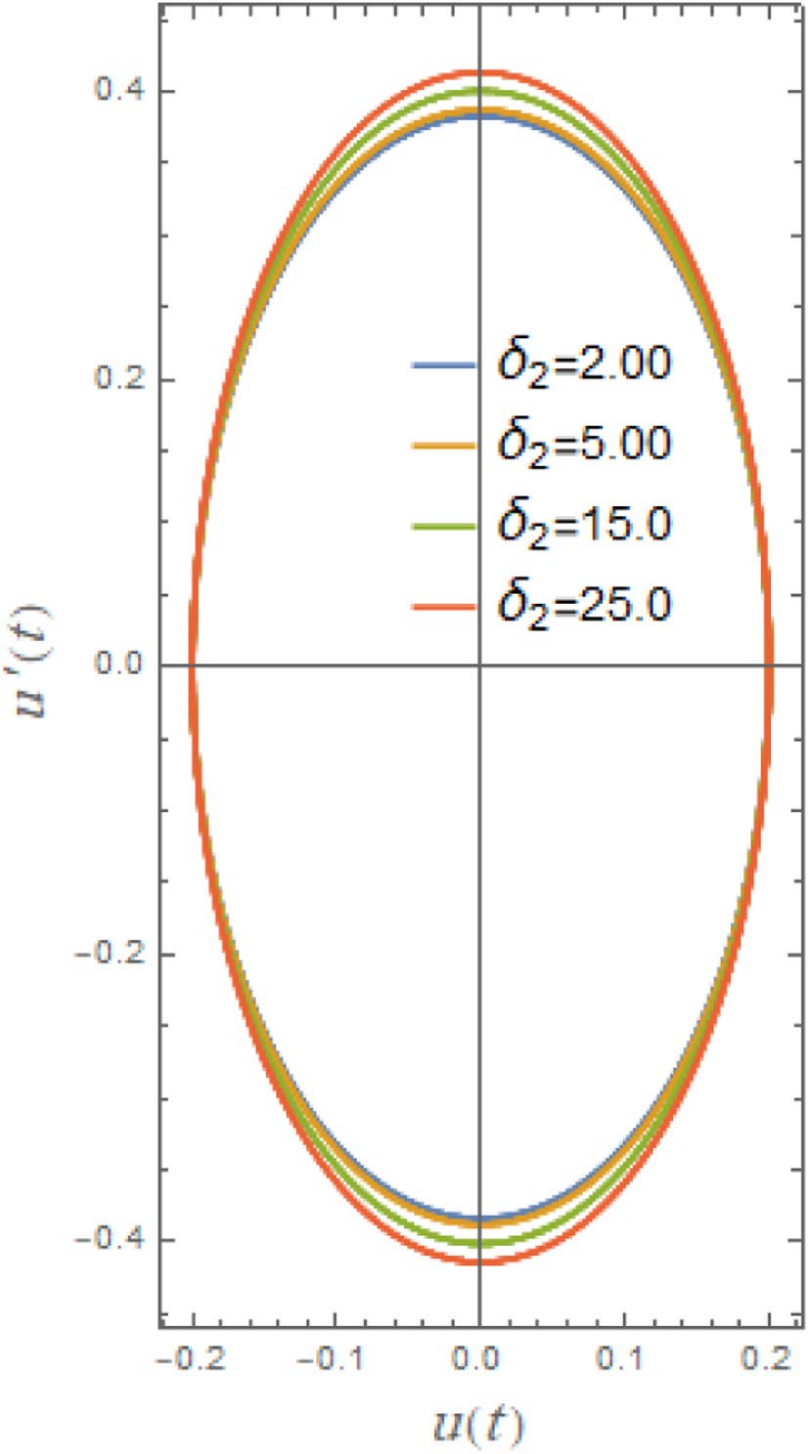
Indicates the influence $\mu$ on the phase plane $uu^{\prime}$ of $u(t)$.

**Fig. 24:**
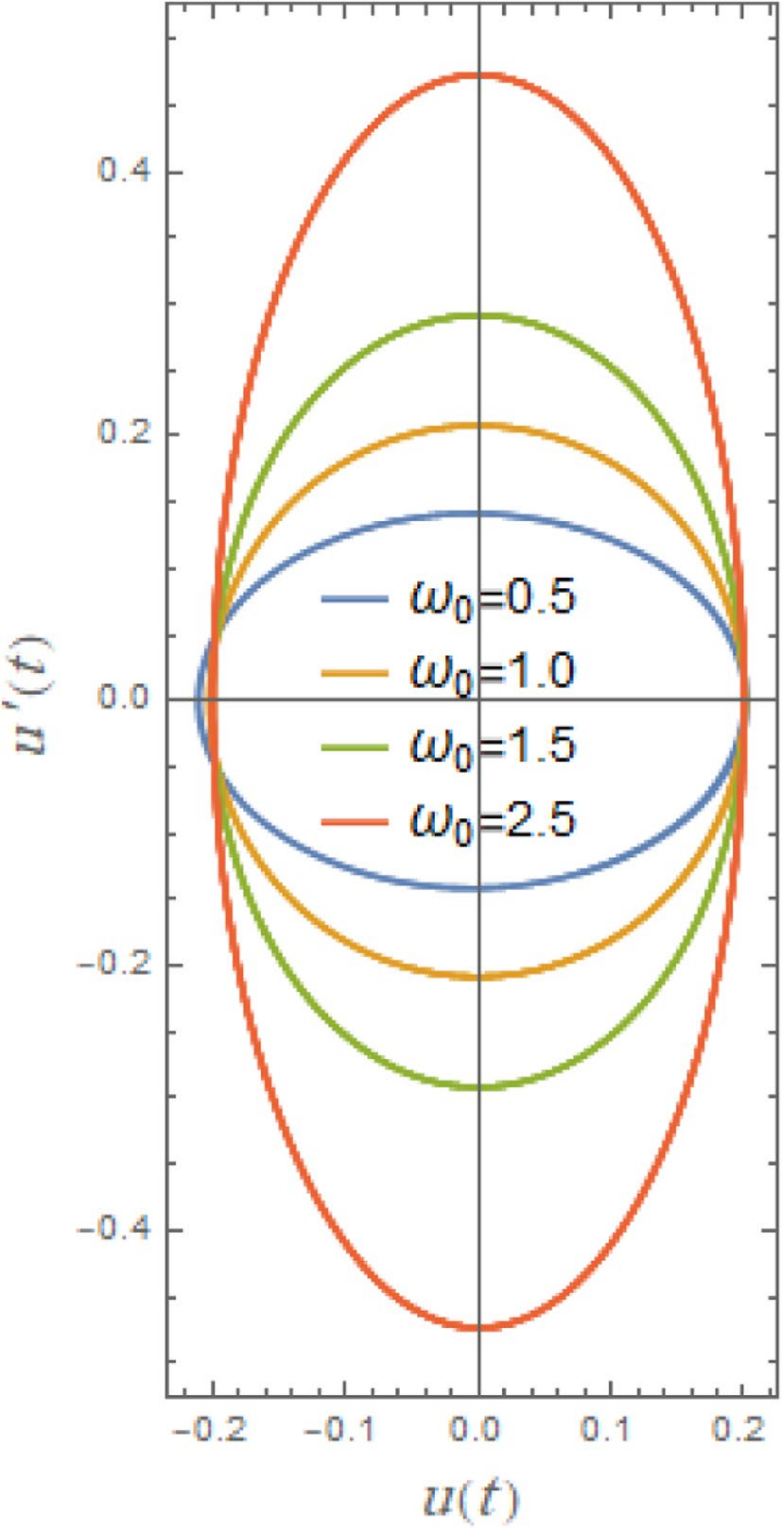
Indicates the influence of $\omega_{0}$ on the phase plane $uu^{\prime}$ of $u(t)$.

**Fig. 25:**
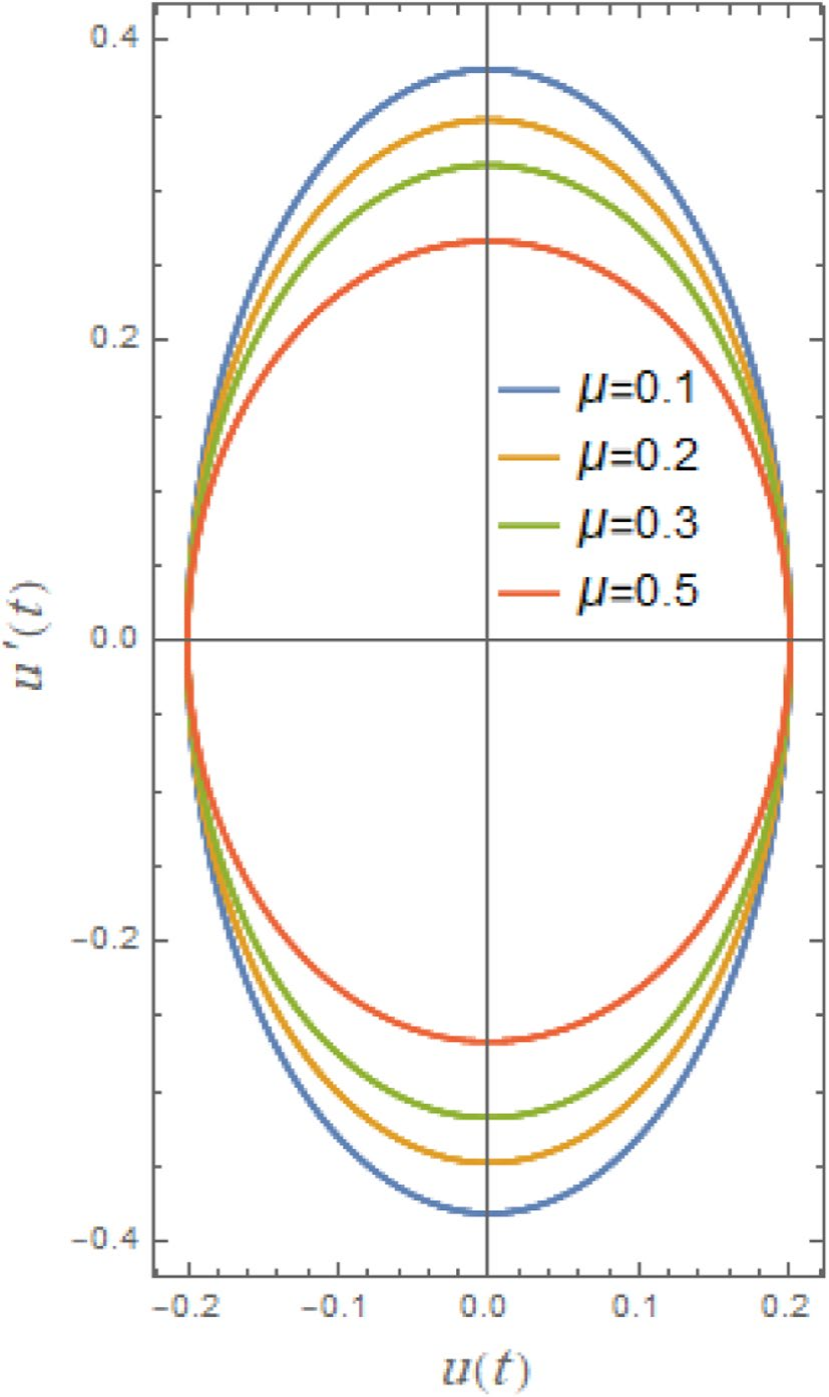
Indicates the influence of $\mu$ on the phase plane $uu^{\prime}$ of $u(t)$.

**Fig. 26:**
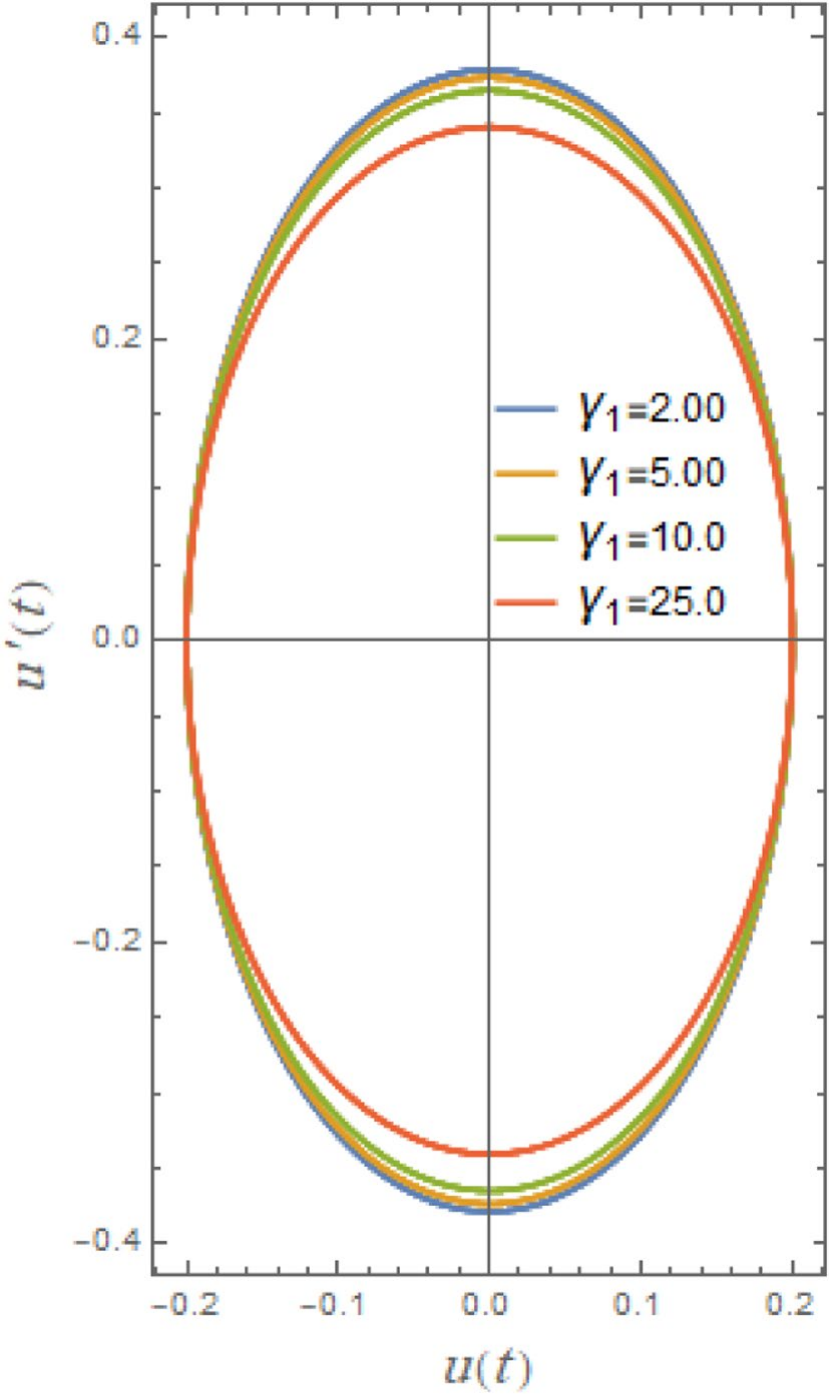
Indicates the influence of $\mu$ on the phase plane $uu^{\prime}$ of $u(t)$.

Phase plane diagrams showing the parametric connections between the equivalent function and its time derivative in a damped situation are shown in Figs. 21, 22, 23, 24, 25, 26. These phase plane diagrams’ key components are supplied by:

In a phase-plane diagram, displacement is shown on the horizontal axis and velocity on the vertical axis, with each trajectory illustrating a potential system state progressing over time from its beginning condition in accordance with the system’s dynamics. Fixed equilibrium points dictate the flow: outward-spiraling trajectories signify an unstable node or spiral, inward spirals denote a stable node, and bifurcating routes represent a saddle point. Periodic solutions manifest as closed loops indicative of prolonged oscillations, such as limit cycles in nonlinear systems, whereas damping induces trajectories to spiral inward towards equilibrium. In nonlinear systems, trajectories may exhibit complexity and non-recurrence, with cubic or quintic nonlinearities resulting in phenomena such as chaos, multi-stability, or asymmetry that alter the trajectories.

Figures 21, 22, 23, 24, 25, 26 show how stability or steady-state characteristics of solutions vary as different parameters are increased by symmetric circular loops around vertical and horizontal axes. The phase plane diagrams of $u(t)$ versus $u^{\prime}(t)$ are presented in Fig. 21 together with initial amplitude $A$ increase. With the growth of $A$, it is evident from this picture that the confined loops are substantially growing near the trajectories’ center. In nonlinear systems, including those described by nonlinear restoring moment and nonlinear damping, initial roll motion amplitude greatly affects how a ship’s rolling dynamics behave. For small initial amplitudes, as seen in Fig. 21, the trajectory often exhibits regular, stable patterns and remains around the origin. As observed in Fig. 21, trajectories typically exhibit regular, stable patterns for modest initial amplitudes and remain around origin. With large initial amplitude, these trajectories become unstable and may deviate or act untidily, creating enormous circles that are bounded by a limit phase.

Figure 22 indicates the impact of the additive frequency $\delta_{1}$, which is important even at small roll angles. It is found that the increase of $\delta_{1}$ enlarges restoring moment in rolling equation. The destabilizing effect of additional frequency coefficient, as previously noted, is a crucial element of ship rolling dynamics, especially when external wave stimulation occurs close to the system’s frequency. Strongly associated with resonance, this phenomenon may lead to excessive roll motions, thus jeopardizing the stability of the ship. The rise of additive frequency parameter $\delta_{1}$ deviates from natural frequency of system, causing nonlinear resonance activities and destabilization of phase plane trajectories.

Figure 23 illustrates the impact of cubic parameter $\delta_{2}$, which increases rolling equation’s restoring moment and at bigger roll angles it becomes more important. By adding this term, the system can account of nonlinearities in restoring force caused by hull curvature of ship and hydrostatic effects. In order to obtain the impact, it is evident from this figure that the routes of the phase plane grow as $\delta_{2}$ rises throughout a wide range of 2.0 to 25.0. The cubic parameter shifts the natural frequency of system, especially at big roll angles, causing nonlinear resonance occurrences and destabilizing phase plane trajectories.

Furthermore, Fig. 24 indicates the phase plane diagram under influence of natural frequency $\omega_{0}$. It is noted that loops are growing about the center here with a rise of $\omega_{0}$ from 0.5 to 2.5. The phase plane trajectories are powerfully affected by natural frequency of a vessel’s roll motion, particularly when nonlinear stability analysis is included. The system oscillates faster as the natural frequency rises. Accordingly, phase plane trajectories near equilibrium points seem more compressed and have a larger curvature. Furthermore, when roll-damping is nonlinear, raising natural frequency may cause limits of stability to change, increasing likelihood that the system would experience chaotic or transitory instability before stabilizing into oscillations.

Figure 25 makes clear the influence of the linear damping factor $\mu$, which is the rolling motion’s primary damping parameter and improves system stability significantly, on phase plane trajectories. It is shown that phase plane’s pathways decrease as $\mu$ grows within a narrow range of 0.1 to 0.5. As previously seen in Figs. 6 and 18, damping element is readily thought of as a stability factor of the system, so the growth of it produces uniform elliptical trajectories that approach the center with the rise of $\mu$. Physically, damping reduces oscillations by removing energy from the system. Smaller damping prolongs oscillations, while larger damping accelerates energy loss. As shown in Fig. 26, the impure damping coefficient has the same impact; the trajectories show minimal decay, which is consistent with earlier results in the stability and time history diagrams.

According to the above consequences the physical implications and practical relevance of our theoretical findings could be summarized as follows:

The physical importance of nonlinear roll damping is underscored by correlating actual hydrodynamic forces on a vessel with linear, quadratic, and cubic damping terms, which account for energy dissipation due to wave resistance, flow separation, and viscous effects, elements that are especially critical at substantial roll amplitudes in turbulent waters. The work elucidates how variations in natural frequency, damping coefficients, and both linear and nonlinear restoring stiffness affect stability, indicating that while an elevated natural frequency may seem stabilizing at first, it can incite resonant amplification in nonlinear contexts and result in perilous roll excursions. This discussion links concepts to real phenomena like parametric rolling and broaching, illustrating how dynamic fluctuations in restoring forces and wave excitation can lead to sudden stability loss, with the nonlinear model elucidating the mechanisms underlying these occurrences. The practical significance is underscored by detailing how the model aids in initial ship design, delineates safe operating parameters, evaluates anti-roll measures, and forecasts susceptibility to capsizing under particular sea conditions. Furthermore, phase-plane plots, time-history diagrams, and energy profiles have been integrated to visually connect the theoretical framework with observable rolling behavior.

## MTSM

This section addresses the solution of the advanced linear ODE as given in Eq. (26) by employing the MTSM^50^ as a pioneer perturbation method, aiming to derive a solution up to the second approximation.

We start with the assumption that the solution to the equation of the mentioned model will depend on the small parameter $\varepsilon$ as follows.

31 $$U(t) = \varepsilon \,z(\tau ;\varepsilon )$$

The function $z$ expressed in powers of $\varepsilon$ can be developed as follows using the MTSM as

32 $$z(\tau ) = \sum\limits_{j = 1}^{2} {\varepsilon^{j} z_{j} (\tau_{0} ,\,\tau_{1} )} + O(\varepsilon^{3} )$$

In this context, $\tau_{j} = \varepsilon^{j} t,\,\,j = 0,1$, where $\tau_{0}$ corresponding to the fast time scale and $\tau_{1}$ to the slow time scale. Therefore, all derivatives with respect to $\tau$ are expressed in the following form^51^:

33 $$\left. \begin{gathered} \frac{d}{d\tau } \equiv \frac{\partial }{{\partial \tau_{0} }} + \varepsilon \frac{\partial }{{\partial \tau_{1} }} \hfill \\ \frac{{d^{2} }}{{d\tau^{2} }} \equiv \frac{{\partial^{2} }}{{\partial \tau_{0}^{2} }} + 2\varepsilon \frac{{\partial^{2} }}{{\partial \tau_{0} \partial \tau_{1} }} + O(\varepsilon^{3} ) \hfill \\ \end{gathered} \right\}$$

Furthermore, all parameters are modified in the power of $\varepsilon$ as follows

34 $$\hat{\mu } = \varepsilon \mu_{eqv} ,\,\,\hat{F} = \varepsilon^{2} F$$

The following three PDEs are derived by substituting Eqs. (31)–(34) into Eq. (26) and matching the coefficients of like powers of $\varepsilon$ on both sides.

35 $$O(\zeta ):\,\frac{{\partial ^{2} z_{1} }}{{\partial \tau _{0}^{2} }} + \omega _{{eqv}}^{2} {\mkern 1mu} z_{1} = 0$$

36 $$O(\zeta ^{2} ):\,\frac{{\partial ^{2} z_{2} }}{{\partial \tau _{0}^{2} }} + \omega _{{eqv}}^{2} z_{2} = \frac{{F{\mkern 1mu} }}{2}e^{{i{\kern 1pt} \sigma {\kern 1pt} \tau _{0} }} - 2\mu _{{eqv}} \frac{{\partial z_{1} }}{{\partial \tau _{0} }} - 2\frac{{\partial ^{2} z_{1} }}{{\partial \tau _{0} \partial \tau _{1} }}$$

The earlier PDEs can be solved consecutively, enabling us to derive the solutions for the first and second approximations as

37 $$z_{1} = B\,e^{{i\,\omega_{eqv} \tau_{0} }} + \overline{B}\,e^{{ - i\,\omega_{eqv} \tau_{0} }}$$

38 $$z_{2} = \frac{{F\,e^{{i\,\,\sigma \,\tau_{0} }} }}{{2(\omega_{eqv}^{2} - \sigma^{2} )}} + \frac{{F\,e^{{ - i\,\,\sigma \,\tau_{0} }} }}{{2(\omega_{eqv}^{2} - \sigma^{2} )}}$$

where $B\,(\tau_{1} )$ is an unknown function, and its complex conjugate is denoted by $\overline{B}\,(\tau_{1} )$. Moreover, the following secular terms are obtained from the second-order approximation Eq. (36) as:

39 $$2i\,\omega_{eqv} \,\mu_{eqv} B + 2i\,\omega_{eqv} \frac{\partial B}{{\partial \tau_{1} }} = 0$$

It is worth noting that the MTSM is a powerful analytical technique for studying the dynamics of nonlinear systems, particularly when the system exhibits behavior on different time scales, as is the case with our studied model. By introducing distinct time variables, MTSM effectively separates fast and slow dynamics, allowing for a more accurate approximation of the system’s response over extended periods. One of its key advantages is the elimination of secular terms, which can otherwise cause the solution to grow unbounded and become invalid over time. This ensures the uniform validity of the approximate solution.

### Investigation of primary external resonance

External resonance occurs when an external force is applied to a system at a frequency that matches the system’s natural frequency. Under this condition, the denominators in the perturbation solution may tend toward zero, leading to a substantial amplification of the system’s response, a phenomenon known as resonance^52^. In the context of our model, this resonance condition is observed when $\sigma \approx \omega_{eqv}$, potentially resulting in large amplitude oscillations due to energy accumulation over time.

We introduce the detuning parameter $\varpi$ as follow

40 $$\sigma = \omega_{eqv} + \varpi ,\,\,\,\varpi = \varepsilon \hat{\varpi }$$

We will find the solvability condition by substituting Eq. (40) into Eq. (36), resulting in the following:

41 $$\frac{1}{2}Fe^{{\tau_{1} \hat{\varpi }}} + 2i\omega_{eqv} \mu_{eqv} B + 2i\omega_{eqv} \frac{\partial B}{{\partial \iota_{1} }} = 0$$

We will examine the subsequent polar form as outlined below

42 $$B = \frac{1}{2}\tilde{b}e^{{i\tilde{\psi }}} ,\,\,b = \varepsilon \tilde{b}$$

here, the amplitude and phase of the solution $z$ are represented by $\tilde{b}$ and $\tilde{\psi }$, respectively. Meanwhile, the modified phase can be expressed as

43 $$\theta = \tau \,\varpi - \psi$$

The modulation equations are derived by substituting Eqs. (42) and (43) into Eq. (41) and separating the imaginary and real components as shown:

44 $$\left. \begin{gathered} b\frac{d\theta }{{d\tau }} = b\,\varpi - \frac{{\tilde{F}}}{{2\omega_{eqv} }}\cos \theta \hfill \\ \frac{d\,b}{{d\tau }} = - \frac{1}{2}\mu_{eqv} \,b - \frac{{\tilde{F}}}{{2\omega_{eq} }}\sin \theta \hfill \\ \end{gathered} \right\}$$

### Stability analysis

This section seeks to evaluate the stability of the model being examined. To perform this evaluation, we must first analyze the steady-state condition of the system. This involves setting all derivatives in Eq. (44) to zero. Additionally, we reformulated the original set of Eqs. (44) under steady-state conditions into a single algebraic equation that is independent of $\theta$, as demonstrated below:

45 $$\tilde{F}^{2} = 4b^{2} \omega_{eqv}^{2} (\,\tilde{\mu }_{eqv}^{2} \, + \varpi^{2} )\,\,$$

To perform the stability analysis, a small perturbation is introduced to $b$ and $\theta$, enables the investigation of the system’s behavior in the vicinity of the fixed point $b$ and its response to minor deviations from equilibrium^53^. Accordingly, the following assumption is made: $b_{0} \,\,$ and $\theta_{0}$ represent the steady-state solutions, while the corresponding perturbed quantities are denoted $b_{1}$ and $\theta_{1}$.

46 $$b = b_{0} + b_{1} ,\,\theta = \theta_{0} + \theta_{1}$$

After substituting Eq. (46) into Eq. (44), we obtained the following linearized system.

47 $$\left. \begin{gathered} \omega_{eqv} \,b_{0} \frac{{d\theta_{1} }}{d\tau } = b_{1} \,\varpi \,\omega_{eqv} + \frac{{\tilde{F}}}{2}\sin \theta_{0} \theta_{1} \hfill \\ \omega_{eqv} \frac{{db_{1} }}{d\tau } = - \omega_{eqv} \,\tilde{\mu }_{eqv} \,b_{1} - \frac{{\tilde{F}}}{2}\theta_{1} \cos \theta_{0} \hfill \\ \end{gathered} \right\}$$

The Routh-Hurwitz criterion^54^ serves as a robust analytical method for evaluating system stability by examining the characteristic equation derived from the transfer function.

48 $$\zeta^{2} + \Gamma_{1} \zeta + \Gamma_{2} = 0$$

Given the linear nature of the perturbed functions $b_{1}$ and $\,\theta_{1}$ in the system as presented in Eq. (47), their solutions are assumed to take the form $K_{j} \,e^{\zeta \,\tau }$, where $\zeta$ is the eigenvalue associated with the perturbation, and $K_{j} ,\,\,(j = 1,2)$ represents constant coefficients. Crucially, the fixed points of the system in Eq. (47) are considered asymptotically stable if the real parts of the roots of the corresponding characteristic equation are negative. According to the Routh-Hurwitz criterion, the following conditions are both necessary and sufficient to ensure the stability of these fixed points:

49 $$\left. \begin{gathered} \Gamma_{{1}} > 0 \hfill \\ \Gamma_{{1}} \Gamma_{{2}} > 0 \hfill \\ \end{gathered} \right\}$$

where, $$\Gamma_{{1}} = \tilde{\mu }_{eqv} - \frac{{\tilde{F}\,\sin \theta_{0} }}{{2\,b_{0} \,\omega_{eqv} }}$$ and $$\Gamma_{{2}} = \frac{{\tilde{F}\,(\varpi \cos \theta_{0} - \tilde{\mu }_{eqv} \sin \theta_{0} )}}{{2\,b_{0} \,\omega_{eqv} }}$$.

**Fig. 27:**
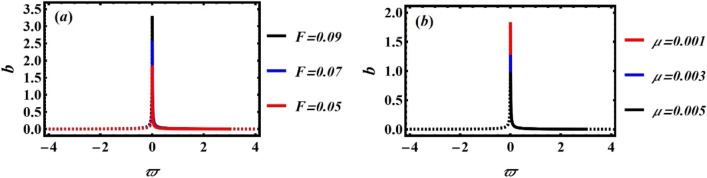
Resonance curves under the influence of variation of (*a*) $F$ and (*b*) $\mu$.

The nonlinear stability of the proposed model has been investigated, with particular attention to the significant effects of key parameters such as the forcing amplitude $F$ and the damping coefficient $\mu$. Figure 27 illustrates simulated resonance curves that showcase the system’s nonlinear stability behavior. In these charts, solid lines represent stable regions, whereas dotted lines mark the areas of instability.

In subplot (*a*), the impact of varying the forcing amplitude is illustrated for three different values of $F$ (0.05, 0.07, and 0.09). It is evident that increasing $F$ leads to a higher peak in the response amplitude $b$, reflecting the system’s intensified reaction to stronger external excitation. The resonance peak becomes sharper and more pronounced within the stable region $\varpi \in [0,3]$, while the instability persists in the regions $\varpi \in [ - 4,0]$ and $\varpi \in [3,4]$, as indicated by the dotted segments of the curves. This behavior demonstrates that while the size of the unstable region remains relatively constant, higher forcing levels exacerbate the system’s susceptibility to large oscillations in the stable region due to increased energy input.

In subplot (*b*), the damping parameter $\mu$ is varied (0.001, 0.003, and 0.005) to assess its effect on the system’s dynamic response. As expected, increasing the damping coefficient significantly reduces the amplitude of the resonance peak, thereby mitigating the system’s oscillatory behavior. While the boundaries of the unstable regions remain fixed, the amplitude of the unstable response is noticeably diminished with higher damping, indicating an enhanced degree of overall stability. These results suggest that damping acts as a stabilizing factor, effectively controlling the system’s response near resonance and reducing the likelihood of dynamic instability. In summary, the forcing amplitude $F$ amplifies the system’s response and increases sensitivity to resonance without changing the instability range, whereas the damping parameter $\mu$ suppresses large oscillations and promotes dynamic stability across both stable and unstable regimes.

## Bifurcation analysis of the general case

In this section, we demonstrate that analyzing the chaotic behavior^54–56^ of a dynamical model is essential for understanding the system’s sensitivity to initial conditions and parameter variations. The bifurcation diagram illustrates how qualitative changes in the system’s dynamics emerge as a control parameter varies. To this end, we construct the bifurcation diagram for the nonlinear Eq. (5) to identify the different types of motion and to highlight the parameter ranges in which the system exhibits periodic or chaotic behavior. It should be noted that the bifurcation analysis is performed numerically (using the complete nonlinear model) with the same set of parameters presented earlier in the paper.

**Fig. 28:**
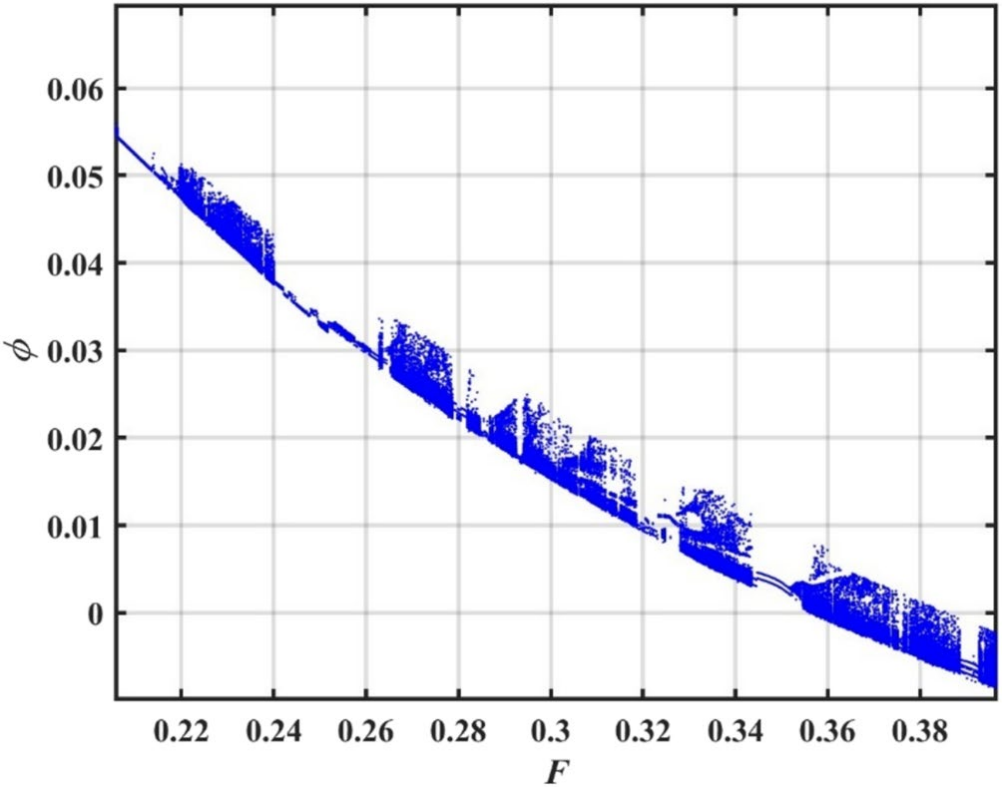
Bifurcation diagram of the studied model.

In this section, we consider the excitation amplitude $F$ as the bifurcation parameter, as shown in Fig. 28. The bifurcation diagram reveals that the system undergoes transitions between different types of motion as $F$ varies. Specifically, as $F < 0.22$, the system exhibits periodic behavior, which is evident from the presence of a single line in the bifurcation diagram, indicating a stable periodic orbit. However, as $F$ increases beyond 0.22, the system transitions into a chaotic regime, reflected by the scattered and irregular points in the diagram. This transition suggests a loss of stability of the periodic orbit and the onset of sensitive dependence on initial conditions, which is a hallmark of chaos.

**Fig. 29:**
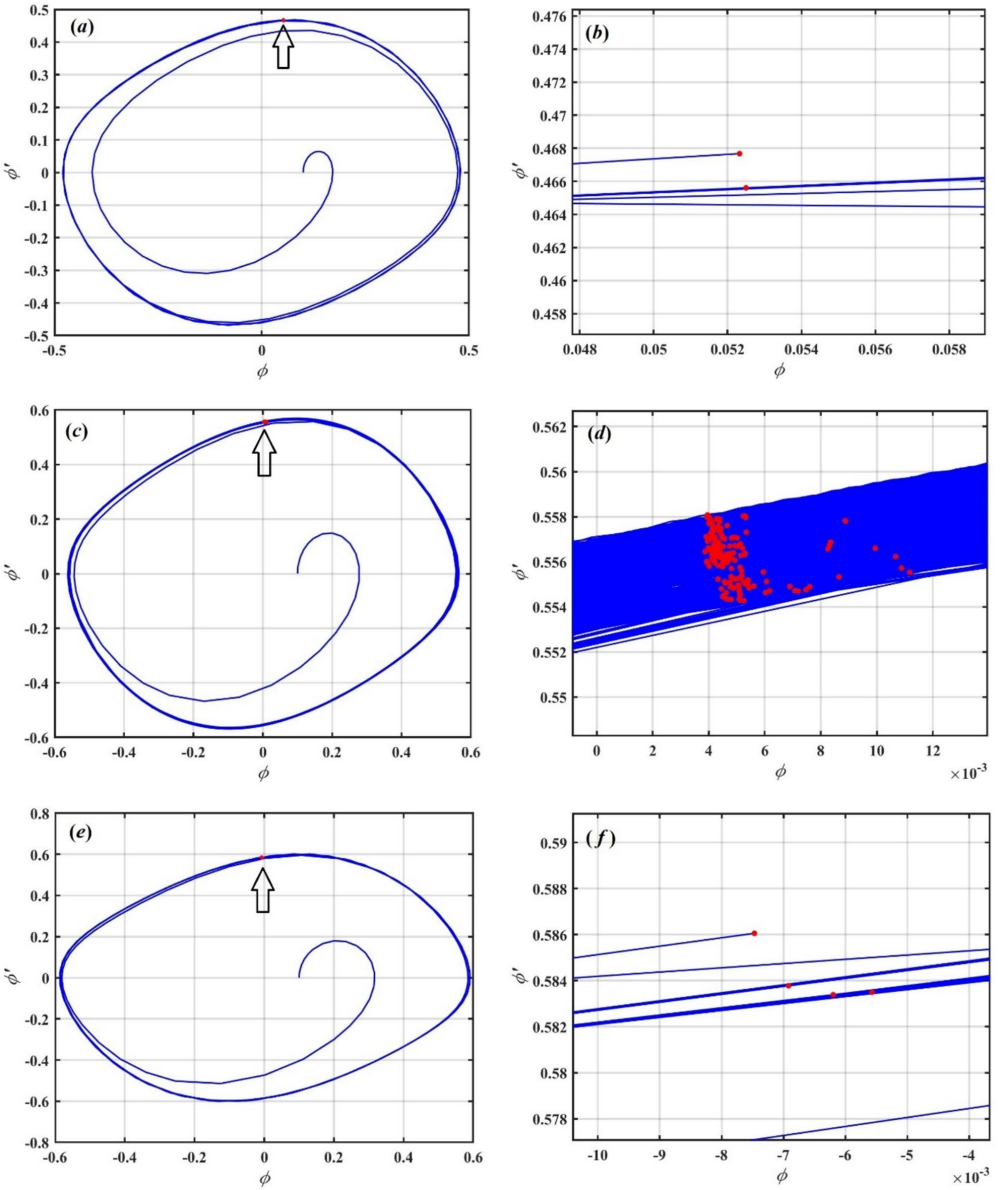
Phase portraits and Poincaré maps illustrating the system’s behavior. Panels (**a**) and its zoomed-in view (**b**) depict periodic motion; (**c**) and its zoomed-in view (**d**) show chaotic behavior, while (**e**) and its zoomed-in view (**f**) illustrate period-4 motion.

To further validate and interpret these findings, we present phase portraits and Poincaré maps in Fig. 29. Phase portraits (blue curves) help visualize the evolution of the system’s state over time, revealing the nature and geometry of attractors, while Poincaré maps (red dots) act as a stroboscopic view of the system’s dynamics, significantly simplifying the detection of periodicity and chaos. These tools together provide a clearer understanding of the underlying nonlinear behavior of the system.

Figure 29a and b show the phase portrait and its zoomed-in Poincaré map for a case where the system exhibits periodic motion at $F = 0.21$. The Poincaré section shows two distinct red points, indicating a period-2 orbit, where the system repeats its behavior every two cycles. This regularity confirms the periodic nature of the motion predicted by the bifurcation diagram.

In contrast, Fig. 29c and d display the system’s response in the chaotic regime when $F = 0.34$. Here, the red dots in the Poincaré map are distributed irregularly, forming a fractal-like structure. This reflects the unpredictability of the system’s evolution and its sensitive dependence on initial conditions-key characteristics of chaotic dynamics. The corresponding phase portrait appears disordered, lacking the regular structure seen in the periodic case, further confirming the chaotic state.

Additionally, we observe period-doubling bifurcations in certain regions of the bifurcation diagram, particularly near $F = 0.35$ and $F = 0.39$. This scenario is typical in nonlinear systems undergoing a transition to chaos through a cascade of period-doubling bifurcations. At $F = 0.39$, the system clearly exhibits period-4 motion, as seen in Fig. 29e and f, where the Poincaré map shows four discrete red points. This confirms that the system is undergoing a structured route to chaos, passing through intermediate periodic states before reaching fully chaotic behavior.

The detailed analysis using bifurcation diagrams, phase portraits, and Poincaré maps not only helps identify the dynamic regimes but also provides deeper insight into the system’s complex behavior. Understanding these transitions is essential, especially for engineering applications where chaotic responses may lead to unpredictable or undesirable performance. The identification of stable and unstable regions can aid in designing control strategies to either avoid chaos or exploit it for beneficial purposes such as energy harvesting or system optimization.

Simulating the bifurcation diagram, phase portraits, and Poincaré maps of a vessel with general roll-damping provides valuable insights into the system’s nonlinear dynamic behavior, especially under various wave and control conditions. The bifurcation diagram helps identify critical parameter values where the system transitions between qualitatively different behaviors such as from periodic to chaotic roll motion highlighting the onset of instability or loss of control. Phase portraits visually represent the system’s state-space trajectories, offering a clear understanding of attractors, limit cycles, and the nature of the roll response (e.g., damped, sustained, or divergent motion). Meanwhile, Poincaré maps simplify complex dynamics into discrete snapshots, making it easier to detect periodicity or chaos. Together, these tools enable engineers to design safer, more efficient damping strategies, optimize control parameters, and prevent dangerous resonant or chaotic roll motions during real maritime operations.

## Conclusions

The evaluation of a vessel, focusing on general roll-damping, seeks to enhance performance, safety, and stability through an extensive analysis and modelling of roll-damping mechanisms. It develops an extensive model that integrates operational conditions, nonlinearities, and hydrodynamic variables. The model included 1DOF nonlinear ship dynamics. The model encompassed inertia, damping, restoring forces, and external forces. The objective was to examine the dependable NPA in determining the periodic response of a damped and conservative coupled system. HFF was the principal factor influencing the NPA. In contrast to all other traditional perturbation methods, the NPA aimed to convert a nonlinear ODE into a linear format without utilizing Taylor expansion. The MS was utilized to validate the resulting parametric linear ODE, exhibiting significant agreement with the original NS. NPA was defined as direct, engaging, positive, strong, and persuasive. It was applicable to fluid mechanics and several interrelated dynamical systems. The nonlinear forced-damped oscillator can be linearized using a similar method, yielding a linear ODE; however, this was insufficient for resonance response analysis. Quantitative analyses validated that the ultimate response corresponded with a sophisticated solution. The resonance area, located within the stability zone, demonstrated complex interactions among forces in the system and was affected by all physical parameters. The influence of several parameters on steady-state stability is investigated. A second-order approximate solution was derived using MTSM, which evaluates the system’s stability configuration and distinguishes between stable and unstable properties of regions. Alterations in bifurcation parameters affect the curvature of the bifurcation curve. Phase portraits, Poincaré maps, and bifurcation diagrams were utilized to perform a bifurcation analysis of the designated models. This method enabled us to recognize and differentiate the unique motion patterns of each machine. The following is a summary of the primary results:

1. The damped nonlinear oscillator that regulated the relative angle of the ship-rolling phenomenon and its associated linear form was demonstrated using NPA.
2. The efficiency of the current approach was demonstrated by confirmation of the equivalence between NPA and NS for both regular and advanced approaches.
3. The advanced trial solution has more matching than the standard one.
4. In the advanced state, the stability configuration was computed considering the effects of different parameters.
5. To increase viability, different parameter variations were used to depict time history and phase plane configurations.
6. It was discovered that as the natural frequency, linear and nonlinear restoring force coefficients increased, the stability zones decreased. Furthermore, it was discovered that these regions grew as linear and nonlinear damping factors increased.
7. The MTSM allows us to get approximate solutions up to second order. Additionally, its application has facilitated a systematic stability analysis, enabling a clear distinction between stable and unstable regions through resonance curves in the system’s parameter space.
8. The forcing amplitude amplified the system’s response and increased its sensitivity to resonance without affecting the instability range; meanwhile, the damping parameter suppresses large oscillations and promotes dynamic stability in both stable and unstable regimes.
9. As the bifurcation parameter rises, the system exhibits a shift from periodic to chaotic behavior, according to the bifurcation analysis of the dynamical model’s initial equation. Period-doubling bifurcations up to period-4 are occasionally observed in the system. Phase portraits, Poincaré maps, and bifurcation diagrams were used to validate these dynamic transitions.
10. The current study’s findings showed that the approach defined here was simple, straightforward, motivating, and successful. A wide range of nonlinear dynamical systems may benefit from its application.

Examining the nonlinear stability of a vessel with generalized roll-damping over several degrees of freedom is crucial, as actual boats undergo coupled motions, such as the interaction of roll with pitch, yaw, or sway, rather than experiencing roll in isolation. This coupling can enhance or diminish roll reactions in ways that linear or 1DOF models cannot anticipate, particularly in the presence of irregular waves, forward velocity, or intricate damping mechanisms. Comprehending these nonlinear, multi-DOF interactions enables designers and operators to foresee unforeseen instabilities such as parametric roll, broaching, or combined resonance, resulting in safer hull designs, more efficient stabilization systems, and operational protocols that accurately represent ship’s dynamic behavior in realistic maritime conditions.

## Supplementary Information

Supplementary Information.

## Data Availability

All data generated or analyzed during this study are included in this manuscript.
