# Supplementary material for: Inspection of stability of a general roll-damping of a ship via non-perturbative approach
Source: Sci Rep. 2026 Feb 23;16:7471. doi: 10.1038/s41598-026-38505-8 (PMC12929613; doi:10.1038/s41598-026-38505-8)
Supplement: Supplementary file 1 — Supplementary Information. [file 41598_2026_38505_MOESM1_ESM.pdf]

$$\tilde{\omega}_{eqv}^{2} = \Big(\pi \Big(512\, A^2 \pi\, \omega_0{}^2 - 1024\, A \pi \gamma\, \omega_0{}^2 +$$

$$1024\, \pi \gamma^2\, \omega_0{}^2 + \frac{1024\, A \gamma\, \omega_0{}^2\, \mathrm{Sin}[\pi^2 \alpha]}{-2 + \pi \alpha} + \frac{1024\, A \gamma\, \omega_0{}^2\, \mathrm{Sin}[\pi^2 \alpha]}{2 + \pi \alpha} -$$

$$\frac{1024\, \gamma^2\, \omega_0{}^2\, \mathrm{Sin}[\pi^2 \alpha]}{-2 + \pi \alpha} - \frac{1024\, \gamma^2\, \omega_0{}^2\, \mathrm{Sin}[\pi^2 \alpha]}{2 + \pi \alpha} + \frac{256\, \gamma^2\, \omega_0{}^2\, \mathrm{Sin}[2 \pi^2 \alpha]}{\pi \alpha} +$$

$$\frac{256 \left(2 \pi^2 \alpha \left(A^2 - 2 A \gamma + 2 \gamma^2\right) + \frac{8 \pi^2 \alpha^2\, (A-\gamma)\, \gamma\, \mathrm{Sin}[\pi^2 \alpha]}{-4+\pi^2 \alpha^2} + \gamma^2\, \mathrm{Sin}[2 \pi^2 \alpha]\right) \delta_1}{\pi \alpha} +$$

$$\Big(32 \Big(-32 \pi^2 \alpha^2 \left(-4 + 9 \pi^2 \alpha^2\right) \gamma\, (-A + \gamma) \Big(4 A^2 \left(-28 + \pi^2 \alpha^2\right) - 8 A \left(-28 + \pi^2 \alpha^2\right) \gamma +$$

$$\left(-220 + 7 \pi^2 \alpha^2\right) \gamma^2\Big)\, \mathrm{Sin}[\pi^2 \alpha] + \left(-36 + \pi^2 \alpha^2\right) \Big(8 \left(-4 + 9 \pi^2 \alpha^2\right) \gamma^2 \Big(6 A^2$$

$$\left(-2 + \pi^2 \alpha^2\right) - 12 A \left(-2 + \pi^2 \alpha^2\right) \gamma + \left(-16 + 7 \pi^2 \alpha^2\right) \gamma^2\Big)\, \mathrm{Sin}[2 \pi^2 \alpha] +$$

$$\left(-4 + \pi^2 \alpha^2\right) \Big(96 \pi^2 \alpha^2\, (A - \gamma)\, \gamma^3\, \mathrm{Sin}[3 \pi^2 \alpha] + \left(-4 + 9 \pi^2 \alpha^2\right) \Big(12 \pi^2 \alpha$$

$$\left(A^4 - 4 A^3 \gamma + 10 A^2 \gamma^2 - 12 A \gamma^3 + 6 \gamma^4\right) + \gamma^4\, \mathrm{Sin}[4 \pi^2 \alpha]\Big)\Big)\Big)\Big)\, \delta_2\Big) \Big/$$

$$(\pi \alpha\, (-6 + \pi \alpha)\, (-2 + \pi \alpha)\, (2 + \pi \alpha)\, (6 + \pi \alpha)\, (-2 + 3 \pi \alpha)\, (2 + 3 \pi \alpha)) +$$

$$320\, A^6 \pi\, \delta_3 - 1920\, A^5 \pi \gamma\, \delta_3 + 7680\, A^4 \pi \gamma^2\, \delta_3 -$$

$$17\,920\, A^3 \pi \gamma^3\, \delta_3 + 24\,960\, A^2 \pi \gamma^4\, \delta_3 -$$

$$19\,200\, A \pi \gamma^5\, \delta_3 + 6400\, \pi \gamma^6\, \delta_3 +$$

$$\frac{192\, A^5 \gamma\, \mathrm{Sin}[\pi^2 \alpha]\, \delta_3}{-10 + \pi \alpha} + \frac{960\, A^5 \gamma\, \mathrm{Sin}[\pi^2 \alpha]\, \delta_3}{-6 + \pi \alpha} +$$

$$\frac{1920\, A^5 \gamma\, \mathrm{Sin}[\pi^2 \alpha]\, \delta_3}{-2 + \pi \alpha} + \frac{1920\, A^5 \gamma\, \mathrm{Sin}[\pi^2 \alpha]\, \delta_3}{2 + \pi \alpha} +$$

$$\frac{960\, A^5 \gamma\, \mathrm{Sin}[\pi^2 \alpha]\, \delta_3}{6 + \pi \alpha} + \frac{192\, A^5 \gamma\, \mathrm{Sin}[\pi^2 \alpha]\, \delta_3}{10 + \pi \alpha} -$$

$$\frac{960\, A^4 \gamma^2\, \mathrm{Sin}[\pi^2 \alpha]\, \delta_3}{-10 + \pi \alpha} - \frac{4800\, A^4 \gamma^2\, \mathrm{Sin}[\pi^2 \alpha]\, \delta_3}{-6 + \pi \alpha} -$$

$$\frac{9600\, A^4 \gamma^2\, \mathrm{Sin}[\pi^2 \alpha]\, \delta_3}{-2 + \pi \alpha} - \frac{9600\, A^4 \gamma^2\, \mathrm{Sin}[\pi^2 \alpha]\, \delta_3}{2 + \pi \alpha} -$$

$$\frac{4800\, A^4 \gamma^2\, \mathrm{Sin}[\pi^2 \alpha]\, \delta_3}{6 + \pi \alpha} - \frac{960\, A^4 \gamma^2\, \mathrm{Sin}[\pi^2 \alpha]\, \delta_3}{10 + \pi \alpha} +$$

$$\frac{1920\, A^3 \gamma^3\, \mathrm{Sin}[\pi^2 \alpha]\, \delta_3}{-10 + \pi \alpha} + \frac{11\,520\, A^3 \gamma^3\, \mathrm{Sin}[\pi^2 \alpha]\, \delta_3}{-6 + \pi \alpha} +$$

$$\frac{24\,960\, A^3 \gamma^3\, \mathrm{Sin}[\pi^2 \alpha]\, \delta_3}{-2 + \pi \alpha} + \frac{24\,960\, A^3 \gamma^3\, \mathrm{Sin}[\pi^2 \alpha]\, \delta_3}{2 + \pi \alpha} +$$

$$\frac{11520\,A^3\,\gamma^3\,\mathrm{Sin}[\pi^2\,\alpha]\,\delta_3}{6+\pi\,\alpha}+\frac{1920\,A^3\,\gamma^3\,\mathrm{Sin}[\pi^2\,\alpha]\,\delta_3}{10+\pi\,\alpha}-$$
$$\frac{1920\,A^2\,\gamma^4\,\mathrm{Sin}[\pi^2\,\alpha]\,\delta_3}{-10+\pi\,\alpha}-\frac{15360\,A^2\,\gamma^4\,\mathrm{Sin}[\pi^2\,\alpha]\,\delta_3}{-6+\pi\,\alpha}-$$
$$\frac{36480\,A^2\,\gamma^4\,\mathrm{Sin}[\pi^2\,\alpha]\,\delta_3}{-2+\pi\,\alpha}-\frac{36480\,A^2\,\gamma^4\,\mathrm{Sin}[\pi^2\,\alpha]\,\delta_3}{2+\pi\,\alpha}-$$
$$\frac{15360\,A^2\,\gamma^4\,\mathrm{Sin}[\pi^2\,\alpha]\,\delta_3}{6+\pi\,\alpha}-\frac{1920\,A^2\,\gamma^4\,\mathrm{Sin}[\pi^2\,\alpha]\,\delta_3}{10+\pi\,\alpha}+$$
$$\frac{960\,A\,\gamma^5\,\mathrm{Sin}[\pi^2\,\alpha]\,\delta_3}{-10+\pi\,\alpha}+\frac{10560\,A\,\gamma^5\,\mathrm{Sin}[\pi^2\,\alpha]\,\delta_3}{-6+\pi\,\alpha}+$$
$$\frac{28800\,A\,\gamma^5\,\mathrm{Sin}[\pi^2\,\alpha]\,\delta_3}{-2+\pi\,\alpha}+\frac{28800\,A\,\gamma^5\,\mathrm{Sin}[\pi^2\,\alpha]\,\delta_3}{2+\pi\,\alpha}+$$
$$\frac{10560\,A\,\gamma^5\,\mathrm{Sin}[\pi^2\,\alpha]\,\delta_3}{6+\pi\,\alpha}+\frac{960\,A\,\gamma^5\,\mathrm{Sin}[\pi^2\,\alpha]\,\delta_3}{10+\pi\,\alpha}-$$
$$\frac{192\,\gamma^6\,\mathrm{Sin}[\pi^2\,\alpha]\,\delta_3}{-10+\pi\,\alpha}-\frac{2880\,\gamma^6\,\mathrm{Sin}[\pi^2\,\alpha]\,\delta_3}{-6+\pi\,\alpha}-\frac{9600\,\gamma^6\,\mathrm{Sin}[\pi^2\,\alpha]\,\delta_3}{-2+\pi\,\alpha}-$$
$$\frac{9600\,\gamma^6\,\mathrm{Sin}[\pi^2\,\alpha]\,\delta_3}{2+\pi\,\alpha}-\frac{2880\,\gamma^6\,\mathrm{Sin}[\pi^2\,\alpha]\,\delta_3}{6+\pi\,\alpha}-\frac{192\,\gamma^6\,\mathrm{Sin}[\pi^2\,\alpha]\,\delta_3}{10+\pi\,\alpha}+$$
$$\frac{1440\,A^4\,\gamma^2\,\mathrm{Sin}[2\,\pi^2\,\alpha]\,\delta_3}{\pi\,\alpha}+\frac{240\,A^4\,\gamma^2\,\mathrm{Sin}[2\,\pi^2\,\alpha]\,\delta_3}{-4+\pi\,\alpha}+$$
$$\frac{960\,A^4\,\gamma^2\,\mathrm{Sin}[2\,\pi^2\,\alpha]\,\delta_3}{-2+\pi\,\alpha}+\frac{960\,A^4\,\gamma^2\,\mathrm{Sin}[2\,\pi^2\,\alpha]\,\delta_3}{2+\pi\,\alpha}+$$
$$\frac{240\,A^4\,\gamma^2\,\mathrm{Sin}[2\,\pi^2\,\alpha]\,\delta_3}{4+\pi\,\alpha}-\frac{5760\,A^3\,\gamma^3\,\mathrm{Sin}[2\,\pi^2\,\alpha]\,\delta_3}{\pi\,\alpha}-$$
$$\frac{960\,A^3\,\gamma^3\,\mathrm{Sin}[2\,\pi^2\,\alpha]\,\delta_3}{-4+\pi\,\alpha}-\frac{3840\,A^3\,\gamma^3\,\mathrm{Sin}[2\,\pi^2\,\alpha]\,\delta_3}{-2+\pi\,\alpha}-$$
$$\frac{3840\,A^3\,\gamma^3\,\mathrm{Sin}[2\,\pi^2\,\alpha]\,\delta_3}{2+\pi\,\alpha}-\frac{960\,A^3\,\gamma^3\,\mathrm{Sin}[2\,\pi^2\,\alpha]\,\delta_3}{4+\pi\,\alpha}+$$
$$\frac{10560\,A^2\,\gamma^4\,\mathrm{Sin}[2\,\pi^2\,\alpha]\,\delta_3}{\pi\,\alpha}+\frac{1440\,A^2\,\gamma^4\,\mathrm{Sin}[2\,\pi^2\,\alpha]\,\delta_3}{-4+\pi\,\alpha}+$$
$$\frac{6720\,A^2\,\gamma^4\,\mathrm{Sin}[2\,\pi^2\,\alpha]\,\delta_3}{-2+\pi\,\alpha}+\frac{6720\,A^2\,\gamma^4\,\mathrm{Sin}[2\,\pi^2\,\alpha]\,\delta_3}{2+\pi\,\alpha}+$$
$$\frac{1440\,A^2\,\gamma^4\,\mathrm{Sin}[2\,\pi^2\,\alpha]\,\delta_3}{4+\pi\,\alpha}-\frac{9600\,A\,\gamma^5\,\mathrm{Sin}[2\,\pi^2\,\alpha]\,\delta_3}{\pi\,\alpha}-$$
$$\frac{960\,A\,\gamma^5\,\mathrm{Sin}[2\,\pi^2\,\alpha]\,\delta_3}{-4+\pi\,\alpha}-\frac{5760\,A\,\gamma^5\,\mathrm{Sin}[2\,\pi^2\,\alpha]\,\delta_3}{-2+\pi\,\alpha}-\frac{5760\,A\,\gamma^5\,\mathrm{Sin}[2\,\pi^2\,\alpha]\,\delta_3}{2+\pi\,\alpha}-$$

$$\frac{960\,A\,\gamma^5\,\text{Sin}\left[2\,\pi^2\,\alpha\right]\,\delta_3}{4+\pi\,\alpha}+\frac{3600\,\gamma^6\,\text{Sin}\left[2\,\pi^2\,\alpha\right]\,\delta_3}{\pi\,\alpha}+\frac{240\,\gamma^6\,\text{Sin}\left[2\,\pi^2\,\alpha\right]\,\delta_3}{-4+\pi\,\alpha}+$$

$$\frac{1920\,\gamma^6\,\text{Sin}\left[2\,\pi^2\,\alpha\right]\,\delta_3}{-2+\pi\,\alpha}+\frac{1920\,\gamma^6\,\text{Sin}\left[2\,\pi^2\,\alpha\right]\,\delta_3}{2+\pi\,\alpha}+\frac{240\,\gamma^6\,\text{Sin}\left[2\,\pi^2\,\alpha\right]\,\delta_3}{4+\pi\,\alpha}+$$

$$\frac{640\,A^3\,\gamma^3\,\text{Sin}\left[3\,\pi^2\,\alpha\right]\,\delta_3}{-6+3\,\pi\,\alpha}+\frac{1920\,A^3\,\gamma^3\,\text{Sin}\left[3\,\pi^2\,\alpha\right]\,\delta_3}{-2+3\,\pi\,\alpha}+$$

$$\frac{1920\,A^3\,\gamma^3\,\text{Sin}\left[3\,\pi^2\,\alpha\right]\,\delta_3}{2+3\,\pi\,\alpha}+\frac{640\,A^3\,\gamma^3\,\text{Sin}\left[3\,\pi^2\,\alpha\right]\,\delta_3}{6+3\,\pi\,\alpha}-$$

$$\frac{640\,A^2\,\gamma^4\,\text{Sin}\left[3\,\pi^2\,\alpha\right]\,\delta_3}{-2+\pi\,\alpha}-\frac{640\,A^2\,\gamma^4\,\text{Sin}\left[3\,\pi^2\,\alpha\right]\,\delta_3}{2+\pi\,\alpha}-\frac{5760\,A^2\,\gamma^4\,\text{Sin}\left[3\,\pi^2\,\alpha\right]\,\delta_3}{-2+3\,\pi\,\alpha}-$$

$$\frac{5760\,A^2\,\gamma^4\,\text{Sin}\left[3\,\pi^2\,\alpha\right]\,\delta_3}{2+3\,\pi\,\alpha}+\frac{640\,A\,\gamma^5\,\text{Sin}\left[3\,\pi^2\,\alpha\right]\,\delta_3}{-2+\pi\,\alpha}+\frac{640\,A\,\gamma^5\,\text{Sin}\left[3\,\pi^2\,\alpha\right]\,\delta_3}{2+\pi\,\alpha}+$$

$$\frac{6720\,A\,\gamma^5\,\text{Sin}\left[3\,\pi^2\,\alpha\right]\,\delta_3}{-2+3\,\pi\,\alpha}+\frac{6720\,A\,\gamma^5\,\text{Sin}\left[3\,\pi^2\,\alpha\right]\,\delta_3}{2+3\,\pi\,\alpha}-\frac{640\,\gamma^6\,\text{Sin}\left[3\,\pi^2\,\alpha\right]\,\delta_3}{-6+3\,\pi\,\alpha}-$$

$$\frac{2880\,\gamma^6\,\text{Sin}\left[3\,\pi^2\,\alpha\right]\,\delta_3}{-2+3\,\pi\,\alpha}-\frac{2880\,\gamma^6\,\text{Sin}\left[3\,\pi^2\,\alpha\right]\,\delta_3}{2+3\,\pi\,\alpha}-\frac{640\,\gamma^6\,\text{Sin}\left[3\,\pi^2\,\alpha\right]\,\delta_3}{6+3\,\pi\,\alpha}+$$

$$\frac{240\,A^2\,\gamma^4\,\text{Sin}\left[4\,\pi^2\,\alpha\right]\,\delta_3}{\pi\,\alpha}+\frac{120\,A^2\,\gamma^4\,\text{Sin}\left[4\,\pi^2\,\alpha\right]\,\delta_3}{-1+\pi\,\alpha}+\frac{120\,A^2\,\gamma^4\,\text{Sin}\left[4\,\pi^2\,\alpha\right]\,\delta_3}{1+\pi\,\alpha}-$$

$$\frac{480\,A\,\gamma^5\,\text{Sin}\left[4\,\pi^2\,\alpha\right]\,\delta_3}{\pi\,\alpha}-\frac{240\,A\,\gamma^5\,\text{Sin}\left[4\,\pi^2\,\alpha\right]\,\delta_3}{-1+\pi\,\alpha}-\frac{240\,A\,\gamma^5\,\text{Sin}\left[4\,\pi^2\,\alpha\right]\,\delta_3}{1+\pi\,\alpha}+$$

$$\frac{288\,\gamma^6\,\text{Sin}\left[4\,\pi^2\,\alpha\right]\,\delta_3}{\pi\,\alpha}+\frac{120\,\gamma^6\,\text{Sin}\left[4\,\pi^2\,\alpha\right]\,\delta_3}{-1+\pi\,\alpha}+\frac{120\,\gamma^6\,\text{Sin}\left[4\,\pi^2\,\alpha\right]\,\delta_3}{1+\pi\,\alpha}+$$

$$\frac{192\,A\,\gamma^5\,\text{Sin}\left[5\,\pi^2\,\alpha\right]\,\delta_3}{-2+5\,\pi\,\alpha}+\frac{192\,A\,\gamma^5\,\text{Sin}\left[5\,\pi^2\,\alpha\right]\,\delta_3}{2+5\,\pi\,\alpha}-\frac{192\,\gamma^6\,\text{Sin}\left[5\,\pi^2\,\alpha\right]\,\delta_3}{-2+5\,\pi\,\alpha}-$$

$$\frac{192\,\gamma^6\,\text{Sin}\left[5\,\pi^2\,\alpha\right]\,\delta_3}{2+5\,\pi\,\alpha}+\frac{16\,\gamma^6\,\text{Sin}\left[6\,\pi^2\,\alpha\right]\,\delta_3}{3\,\pi\,\alpha}+280\,A^8\,\pi\,\delta_4-$$

$$2240\,A^7\,\pi\,\gamma\,\delta_4+12\,320\,A^6\,\pi\,\gamma^2\,\delta_4-42\,560\,A^5\,\pi\,\gamma^3\,\delta_4+96\,880\,A^4\,\pi\,\gamma^4\,\delta_4-$$

$$145\,600\,A^3\,\pi\,\gamma^5\,\delta_4+140\,000\,A^2\,\pi\,\gamma^6\,\delta_4-78\,400\,A\,\pi\,\gamma^7\,\delta_4+19\,600\,\pi\,\gamma^8\,\delta_4+$$

$$\frac{64\,A^7\,\gamma\,\text{Sin}\left[\pi^2\,\alpha\right]\,\delta_4}{-14+\pi\,\alpha}+\frac{448\,A^7\,\gamma\,\text{Sin}\left[\pi^2\,\alpha\right]\,\delta_4}{-10+\pi\,\alpha}+\frac{1344\,A^7\,\gamma\,\text{Sin}\left[\pi^2\,\alpha\right]\,\delta_4}{-6+\pi\,\alpha}+$$

$$\frac{2240\,A^7\,\gamma\,\text{Sin}\left[\pi^2\,\alpha\right]\,\delta_4}{-2+\pi\,\alpha}+\frac{2240\,A^7\,\gamma\,\text{Sin}\left[\pi^2\,\alpha\right]\,\delta_4}{2+\pi\,\alpha}+\frac{1344\,A^7\,\gamma\,\text{Sin}\left[\pi^2\,\alpha\right]\,\delta_4}{6+\pi\,\alpha}+$$

$$\frac{448\,A^7\,\gamma\,\text{Sin}\left[\pi^2\,\alpha\right]\,\delta_4}{10+\pi\,\alpha}+\frac{64\,A^7\,\gamma\,\text{Sin}\left[\pi^2\,\alpha\right]\,\delta_4}{14+\pi\,\alpha}-\frac{448\,A^6\,\gamma^2\,\text{Sin}\left[\pi^2\,\alpha\right]\,\delta_4}{-14+\pi\,\alpha}-$$

$$\frac{3136\,A^6\,\gamma^2\,\text{Sin}\left[\pi^2\,\alpha\right]\,\delta_4}{-10+\pi\,\alpha}-\frac{9408\,A^6\,\gamma^2\,\text{Sin}\left[\pi^2\,\alpha\right]\,\delta_4}{-6+\pi\,\alpha}-\frac{15\,680\,A^6\,\gamma^2\,\text{Sin}\left[\pi^2\,\alpha\right]\,\delta_4}{-2+\pi\,\alpha}-$$

$$\frac{15680\,A^6\,\gamma^2\,\text{Sin}\left[\pi^2\,\alpha\right]\,\delta_4}{2+\pi\,\alpha}-\frac{9408\,A^6\,\gamma^2\,\text{Sin}\left[\pi^2\,\alpha\right]\,\delta_4}{6+\pi\,\alpha}-\frac{3136\,A^6\,\gamma^2\,\text{Sin}\left[\pi^2\,\alpha\right]\,\delta_4}{10+\pi\,\alpha}-$$

$$\frac{448\,A^6\,\gamma^2\,\text{Sin}\left[\pi^2\,\alpha\right]\,\delta_4}{14+\pi\,\alpha}+\frac{1344\,A^5\,\gamma^3\,\text{Sin}\left[\pi^2\,\alpha\right]\,\delta_4}{-14+\pi\,\alpha}+\frac{10752\,A^5\,\gamma^3\,\text{Sin}\left[\pi^2\,\alpha\right]\,\delta_4}{-10+\pi\,\alpha}+$$

$$\frac{34944\,A^5\,\gamma^3\,\text{Sin}\left[\pi^2\,\alpha\right]\,\delta_4}{-6+\pi\,\alpha}+\frac{60480\,A^5\,\gamma^3\,\text{Sin}\left[\pi^2\,\alpha\right]\,\delta_4}{-2+\pi\,\alpha}+\frac{60480\,A^5\,\gamma^3\,\text{Sin}\left[\pi^2\,\alpha\right]\,\delta_4}{2+\pi\,\alpha}+$$

$$\frac{34944\,A^5\,\gamma^3\,\text{Sin}\left[\pi^2\,\alpha\right]\,\delta_4}{6+\pi\,\alpha}+\frac{10752\,A^5\,\gamma^3\,\text{Sin}\left[\pi^2\,\alpha\right]\,\delta_4}{10+\pi\,\alpha}+\frac{1344\,A^5\,\gamma^3\,\text{Sin}\left[\pi^2\,\alpha\right]\,\delta_4}{14+\pi\,\alpha}-$$

$$\frac{2240\,A^4\,\gamma^4\,\text{Sin}\left[\pi^2\,\alpha\right]\,\delta_4}{-14+\pi\,\alpha}-\frac{22400\,A^4\,\gamma^4\,\text{Sin}\left[\pi^2\,\alpha\right]\,\delta_4}{-10+\pi\,\alpha}-\frac{80640\,A^4\,\gamma^4\,\text{Sin}\left[\pi^2\,\alpha\right]\,\delta_4}{-6+\pi\,\alpha}-$$

$$\frac{145600\,A^4\,\gamma^4\,\text{Sin}\left[\pi^2\,\alpha\right]\,\delta_4}{-2+\pi\,\alpha}-\frac{145600\,A^4\,\gamma^4\,\text{Sin}\left[\pi^2\,\alpha\right]\,\delta_4}{2+\pi\,\alpha}-$$

$$\frac{80640\,A^4\,\gamma^4\,\text{Sin}\left[\pi^2\,\alpha\right]\,\delta_4}{6+\pi\,\alpha}-\frac{22400\,A^4\,\gamma^4\,\text{Sin}\left[\pi^2\,\alpha\right]\,\delta_4}{10+\pi\,\alpha}-\frac{2240\,A^4\,\gamma^4\,\text{Sin}\left[\pi^2\,\alpha\right]\,\delta_4}{14+\pi\,\alpha}+$$

$$\frac{2240\,A^3\,\gamma^5\,\text{Sin}\left[\pi^2\,\alpha\right]\,\delta_4}{-14+\pi\,\alpha}+\frac{29120\,A^3\,\gamma^5\,\text{Sin}\left[\pi^2\,\alpha\right]\,\delta_4}{-10+\pi\,\alpha}+\frac{118720\,A^3\,\gamma^5\,\text{Sin}\left[\pi^2\,\alpha\right]\,\delta_4}{-6+\pi\,\alpha}+$$

$$\frac{226240\,A^3\,\gamma^5\,\text{Sin}\left[\pi^2\,\alpha\right]\,\delta_4}{-2+\pi\,\alpha}+\frac{226240\,A^3\,\gamma^5\,\text{Sin}\left[\pi^2\,\alpha\right]\,\delta_4}{2+\pi\,\alpha}+$$

$$\frac{118720\,A^3\,\gamma^5\,\text{Sin}\left[\pi^2\,\alpha\right]\,\delta_4}{6+\pi\,\alpha}+\frac{29120\,A^3\,\gamma^5\,\text{Sin}\left[\pi^2\,\alpha\right]\,\delta_4}{10+\pi\,\alpha}+\frac{2240\,A^3\,\gamma^5\,\text{Sin}\left[\pi^2\,\alpha\right]\,\delta_4}{14+\pi\,\alpha}-$$

$$\frac{1344\,A^2\,\gamma^6\,\text{Sin}\left[\pi^2\,\alpha\right]\,\delta_4}{-14+\pi\,\alpha}-\frac{22848\,A^2\,\gamma^6\,\text{Sin}\left[\pi^2\,\alpha\right]\,\delta_4}{-10+\pi\,\alpha}-\frac{108864\,A^2\,\gamma^6\,\text{Sin}\left[\pi^2\,\alpha\right]\,\delta_4}{-6+\pi\,\alpha}-$$

$$\frac{221760\,A^2\,\gamma^6\,\text{Sin}\left[\pi^2\,\alpha\right]\,\delta_4}{-2+\pi\,\alpha}-\frac{221760\,A^2\,\gamma^6\,\text{Sin}\left[\pi^2\,\alpha\right]\,\delta_4}{2+\pi\,\alpha}-$$

$$\frac{108864\,A^2\,\gamma^6\,\text{Sin}\left[\pi^2\,\alpha\right]\,\delta_4}{6+\pi\,\alpha}-\frac{22848\,A^2\,\gamma^6\,\text{Sin}\left[\pi^2\,\alpha\right]\,\delta_4}{10+\pi\,\alpha}-$$

$$\frac{1344\,A^2\,\gamma^6\,\text{Sin}\left[\pi^2\,\alpha\right]\,\delta_4}{14+\pi\,\alpha}+\frac{448\,A\,\gamma^7\,\text{Sin}\left[\pi^2\,\alpha\right]\,\delta_4}{-14+\pi\,\alpha}+\frac{9856\,A\,\gamma^7\,\text{Sin}\left[\pi^2\,\alpha\right]\,\delta_4}{-10+\pi\,\alpha}+$$

$$\frac{56448\,A\,\gamma^7\,\text{Sin}\left[\pi^2\,\alpha\right]\,\delta_4}{-6+\pi\,\alpha}+\frac{125440\,A\,\gamma^7\,\text{Sin}\left[\pi^2\,\alpha\right]\,\delta_4}{-2+\pi\,\alpha}+\frac{125440\,A\,\gamma^7\,\text{Sin}\left[\pi^2\,\alpha\right]\,\delta_4}{2+\pi\,\alpha}+$$

$$\frac{56448\,A\,\gamma^7\,\text{Sin}\left[\pi^2\,\alpha\right]\,\delta_4}{6+\pi\,\alpha}+\frac{9856\,A\,\gamma^7\,\text{Sin}\left[\pi^2\,\alpha\right]\,\delta_4}{10+\pi\,\alpha}+\frac{448\,A\,\gamma^7\,\text{Sin}\left[\pi^2\,\alpha\right]\,\delta_4}{14+\pi\,\alpha}-$$

$$\frac{64\,\gamma^8\,\text{Sin}\left[\pi^2\,\alpha\right]\,\delta_4}{-14+\pi\,\alpha}-\frac{1792\,\gamma^8\,\text{Sin}\left[\pi^2\,\alpha\right]\,\delta_4}{-10+\pi\,\alpha}-\frac{12544\,\gamma^8\,\text{Sin}\left[\pi^2\,\alpha\right]\,\delta_4}{-6+\pi\,\alpha}-$$

$$\frac{31360\,\gamma^8\,\text{Sin}\left[\pi^2\,\alpha\right]\,\delta_4}{-2+\pi\,\alpha}-\frac{31360\,\gamma^8\,\text{Sin}\left[\pi^2\,\alpha\right]\,\delta_4}{2+\pi\,\alpha}-\frac{12544\,\gamma^8\,\text{Sin}\left[\pi^2\,\alpha\right]\,\delta_4}{6+\pi\,\alpha}-$$

$$\frac{1792\,\gamma^8\,\mathrm{Sin}\left[\pi^2\,\alpha\right]\,\delta_4}{10+\pi\,\alpha}-\frac{64\,\gamma^8\,\mathrm{Sin}\left[\pi^2\,\alpha\right]\,\delta_4}{14+\pi\,\alpha}+\frac{2240\,A^6\,\gamma^2\,\mathrm{Sin}\left[2\,\pi^2\,\alpha\right]\,\delta_4}{\pi\,\alpha}+$$

$$\frac{112\,A^6\,\gamma^2\,\mathrm{Sin}\left[2\,\pi^2\,\alpha\right]\,\delta_4}{-6+\pi\,\alpha}+\frac{672\,A^6\,\gamma^2\,\mathrm{Sin}\left[2\,\pi^2\,\alpha\right]\,\delta_4}{-4+\pi\,\alpha}+\frac{1680\,A^6\,\gamma^2\,\mathrm{Sin}\left[2\,\pi^2\,\alpha\right]\,\delta_4}{-2+\pi\,\alpha}+$$

$$\frac{1680\,A^6\,\gamma^2\,\mathrm{Sin}\left[2\,\pi^2\,\alpha\right]\,\delta_4}{2+\pi\,\alpha}+\frac{672\,A^6\,\gamma^2\,\mathrm{Sin}\left[2\,\pi^2\,\alpha\right]\,\delta_4}{4+\pi\,\alpha}+\frac{112\,A^6\,\gamma^2\,\mathrm{Sin}\left[2\,\pi^2\,\alpha\right]\,\delta_4}{6+\pi\,\alpha}-$$

$$\frac{13\,440\,A^5\,\gamma^3\,\mathrm{Sin}\left[2\,\pi^2\,\alpha\right]\,\delta_4}{\pi\,\alpha}-\frac{672\,A^5\,\gamma^3\,\mathrm{Sin}\left[2\,\pi^2\,\alpha\right]\,\delta_4}{-6+\pi\,\alpha}-\frac{4032\,A^5\,\gamma^3\,\mathrm{Sin}\left[2\,\pi^2\,\alpha\right]\,\delta_4}{-4+\pi\,\alpha}-$$

$$\frac{10\,080\,A^5\,\gamma^3\,\mathrm{Sin}\left[2\,\pi^2\,\alpha\right]\,\delta_4}{-2+\pi\,\alpha}-\frac{10\,080\,A^5\,\gamma^3\,\mathrm{Sin}\left[2\,\pi^2\,\alpha\right]\,\delta_4}{2+\pi\,\alpha}-$$

$$\frac{4032\,A^5\,\gamma^3\,\mathrm{Sin}\left[2\,\pi^2\,\alpha\right]\,\delta_4}{4+\pi\,\alpha}-\frac{672\,A^5\,\gamma^3\,\mathrm{Sin}\left[2\,\pi^2\,\alpha\right]\,\delta_4}{6+\pi\,\alpha}+\frac{40\,320\,A^4\,\gamma^4\,\mathrm{Sin}\left[2\,\pi^2\,\alpha\right]\,\delta_4}{\pi\,\alpha}+$$

$$\frac{1680\,A^4\,\gamma^4\,\mathrm{Sin}\left[2\,\pi^2\,\alpha\right]\,\delta_4}{-6+\pi\,\alpha}+\frac{11\,200\,A^4\,\gamma^4\,\mathrm{Sin}\left[2\,\pi^2\,\alpha\right]\,\delta_4}{-4+\pi\,\alpha}+$$

$$\frac{29\,680\,A^4\,\gamma^4\,\mathrm{Sin}\left[2\,\pi^2\,\alpha\right]\,\delta_4}{-2+\pi\,\alpha}+\frac{29\,680\,A^4\,\gamma^4\,\mathrm{Sin}\left[2\,\pi^2\,\alpha\right]\,\delta_4}{2+\pi\,\alpha}+$$

$$\frac{11\,200\,A^4\,\gamma^4\,\mathrm{Sin}\left[2\,\pi^2\,\alpha\right]\,\delta_4}{4+\pi\,\alpha}+\frac{1680\,A^4\,\gamma^4\,\mathrm{Sin}\left[2\,\pi^2\,\alpha\right]\,\delta_4}{6+\pi\,\alpha}-$$

$$\frac{71\,680\,A^3\,\gamma^5\,\mathrm{Sin}\left[2\,\pi^2\,\alpha\right]\,\delta_4}{\pi\,\alpha}-\frac{2240\,A^3\,\gamma^5\,\mathrm{Sin}\left[2\,\pi^2\,\alpha\right]\,\delta_4}{-6+\pi\,\alpha}-$$

$$\frac{17\,920\,A^3\,\gamma^5\,\mathrm{Sin}\left[2\,\pi^2\,\alpha\right]\,\delta_4}{-4+\pi\,\alpha}-\frac{51\,520\,A^3\,\gamma^5\,\mathrm{Sin}\left[2\,\pi^2\,\alpha\right]\,\delta_4}{-2+\pi\,\alpha}-$$

$$\frac{51\,520\,A^3\,\gamma^5\,\mathrm{Sin}\left[2\,\pi^2\,\alpha\right]\,\delta_4}{2+\pi\,\alpha}-\frac{17\,920\,A^3\,\gamma^5\,\mathrm{Sin}\left[2\,\pi^2\,\alpha\right]\,\delta_4}{4+\pi\,\alpha}-$$

$$\frac{2240\,A^3\,\gamma^5\,\mathrm{Sin}\left[2\,\pi^2\,\alpha\right]\,\delta_4}{6+\pi\,\alpha}+\frac{77\,280\,A^2\,\gamma^6\,\mathrm{Sin}\left[2\,\pi^2\,\alpha\right]\,\delta_4}{\pi\,\alpha}+$$

$$\frac{1680\,A^2\,\gamma^6\,\mathrm{Sin}\left[2\,\pi^2\,\alpha\right]\,\delta_4}{-6+\pi\,\alpha}+\frac{16\,800\,A^2\,\gamma^6\,\mathrm{Sin}\left[2\,\pi^2\,\alpha\right]\,\delta_4}{-4+\pi\,\alpha}+$$

$$\frac{53\,760\,A^2\,\gamma^6\,\mathrm{Sin}\left[2\,\pi^2\,\alpha\right]\,\delta_4}{-2+\pi\,\alpha}+\frac{53\,760\,A^2\,\gamma^6\,\mathrm{Sin}\left[2\,\pi^2\,\alpha\right]\,\delta_4}{2+\pi\,\alpha}+$$

$$\frac{16\,800\,A^2\,\gamma^6\,\mathrm{Sin}\left[2\,\pi^2\,\alpha\right]\,\delta_4}{4+\pi\,\alpha}+\frac{1680\,A^2\,\gamma^6\,\mathrm{Sin}\left[2\,\pi^2\,\alpha\right]\,\delta_4}{6+\pi\,\alpha}-$$

$$\frac{47\,040\,A\,\gamma^7\,\mathrm{Sin}\left[2\,\pi^2\,\alpha\right]\,\delta_4}{\pi\,\alpha}-\frac{672\,A\,\gamma^7\,\mathrm{Sin}\left[2\,\pi^2\,\alpha\right]\,\delta_4}{-6+\pi\,\alpha}-\frac{8512\,A\,\gamma^7\,\mathrm{Sin}\left[2\,\pi^2\,\alpha\right]\,\delta_4}{-4+\pi\,\alpha}-$$

$$\frac{31\,360\,A\,\gamma^7\,\mathrm{Sin}\left[2\,\pi^2\,\alpha\right]\,\delta_4}{-2+\pi\,\alpha}-\frac{31\,360\,A\,\gamma^7\,\mathrm{Sin}\left[2\,\pi^2\,\alpha\right]\,\delta_4}{2+\pi\,\alpha}-\frac{8512\,A\,\gamma^7\,\mathrm{Sin}\left[2\,\pi^2\,\alpha\right]\,\delta_4}{4+\pi\,\alpha}-$$

$$\frac{672\,A\,\gamma^7\,\text{Sin}\left[2\,\pi^2\,\alpha\right]\,\delta_4}{6+\pi\,\alpha}+\frac{12\,544\,\gamma^8\,\text{Sin}\left[2\,\pi^2\,\alpha\right]\,\delta_4}{\pi\,\alpha}+\frac{112\,\gamma^8\,\text{Sin}\left[2\,\pi^2\,\alpha\right]\,\delta_4}{-6+\pi\,\alpha}+$$

$$\frac{1792\,\gamma^8\,\text{Sin}\left[2\,\pi^2\,\alpha\right]\,\delta_4}{-4+\pi\,\alpha}+\frac{7840\,\gamma^8\,\text{Sin}\left[2\,\pi^2\,\alpha\right]\,\delta_4}{-2+\pi\,\alpha}+\frac{7840\,\gamma^8\,\text{Sin}\left[2\,\pi^2\,\alpha\right]\,\delta_4}{2+\pi\,\alpha}+$$

$$\frac{1792\,\gamma^8\,\text{Sin}\left[2\,\pi^2\,\alpha\right]\,\delta_4}{4+\pi\,\alpha}+\frac{112\,\gamma^8\,\text{Sin}\left[2\,\pi^2\,\alpha\right]\,\delta_4}{6+\pi\,\alpha}+\frac{448\,A^5\,\gamma^3\,\text{Sin}\left[3\,\pi^2\,\alpha\right]\,\delta_4}{-10+3\,\pi\,\alpha}+$$

$$\frac{2240\,A^5\,\gamma^3\,\text{Sin}\left[3\,\pi^2\,\alpha\right]\,\delta_4}{-6+3\,\pi\,\alpha}+\frac{4480\,A^5\,\gamma^3\,\text{Sin}\left[3\,\pi^2\,\alpha\right]\,\delta_4}{-2+3\,\pi\,\alpha}+\frac{4480\,A^5\,\gamma^3\,\text{Sin}\left[3\,\pi^2\,\alpha\right]\,\delta_4}{2+3\,\pi\,\alpha}+$$

$$\frac{2240\,A^5\,\gamma^3\,\text{Sin}\left[3\,\pi^2\,\alpha\right]\,\delta_4}{6+3\,\pi\,\alpha}+\frac{448\,A^5\,\gamma^3\,\text{Sin}\left[3\,\pi^2\,\alpha\right]\,\delta_4}{10+3\,\pi\,\alpha}-\frac{2240\,A^4\,\gamma^4\,\text{Sin}\left[3\,\pi^2\,\alpha\right]\,\delta_4}{-10+3\,\pi\,\alpha}-$$

$$\frac{11\,200\,A^4\,\gamma^4\,\text{Sin}\left[3\,\pi^2\,\alpha\right]\,\delta_4}{-6+3\,\pi\,\alpha}-\frac{22\,400\,A^4\,\gamma^4\,\text{Sin}\left[3\,\pi^2\,\alpha\right]\,\delta_4}{-2+3\,\pi\,\alpha}-$$

$$\frac{22\,400\,A^4\,\gamma^4\,\text{Sin}\left[3\,\pi^2\,\alpha\right]\,\delta_4}{2+3\,\pi\,\alpha}-\frac{11\,200\,A^4\,\gamma^4\,\text{Sin}\left[3\,\pi^2\,\alpha\right]\,\delta_4}{6+3\,\pi\,\alpha}-$$

$$\frac{2240\,A^4\,\gamma^4\,\text{Sin}\left[3\,\pi^2\,\alpha\right]\,\delta_4}{10+3\,\pi\,\alpha}+\frac{4480\,A^3\,\gamma^5\,\text{Sin}\left[3\,\pi^2\,\alpha\right]\,\delta_4}{-10+3\,\pi\,\alpha}+$$

$$\frac{24\,640\,A^3\,\gamma^5\,\text{Sin}\left[3\,\pi^2\,\alpha\right]\,\delta_4}{-6+3\,\pi\,\alpha}+\frac{51\,520\,A^3\,\gamma^5\,\text{Sin}\left[3\,\pi^2\,\alpha\right]\,\delta_4}{-2+3\,\pi\,\alpha}+$$

$$\frac{51\,520\,A^3\,\gamma^5\,\text{Sin}\left[3\,\pi^2\,\alpha\right]\,\delta_4}{2+3\,\pi\,\alpha}+\frac{24\,640\,A^3\,\gamma^5\,\text{Sin}\left[3\,\pi^2\,\alpha\right]\,\delta_4}{6+3\,\pi\,\alpha}+$$

$$\frac{4480\,A^3\,\gamma^5\,\text{Sin}\left[3\,\pi^2\,\alpha\right]\,\delta_4}{10+3\,\pi\,\alpha}-\frac{4480\,A^2\,\gamma^6\,\text{Sin}\left[3\,\pi^2\,\alpha\right]\,\delta_4}{-10+3\,\pi\,\alpha}-$$

$$\frac{29\,120\,A^2\,\gamma^6\,\text{Sin}\left[3\,\pi^2\,\alpha\right]\,\delta_4}{-6+3\,\pi\,\alpha}-\frac{64\,960\,A^2\,\gamma^6\,\text{Sin}\left[3\,\pi^2\,\alpha\right]\,\delta_4}{-2+3\,\pi\,\alpha}-$$

$$\frac{64\,960\,A^2\,\gamma^6\,\text{Sin}\left[3\,\pi^2\,\alpha\right]\,\delta_4}{2+3\,\pi\,\alpha}-\frac{29\,120\,A^2\,\gamma^6\,\text{Sin}\left[3\,\pi^2\,\alpha\right]\,\delta_4}{6+3\,\pi\,\alpha}-$$

$$\frac{4480\,A^2\,\gamma^6\,\text{Sin}\left[3\,\pi^2\,\alpha\right]\,\delta_4}{10+3\,\pi\,\alpha}+\frac{2240\,A\,\gamma^7\,\text{Sin}\left[3\,\pi^2\,\alpha\right]\,\delta_4}{-10+3\,\pi\,\alpha}+\frac{17\,920\,A\,\gamma^7\,\text{Sin}\left[3\,\pi^2\,\alpha\right]\,\delta_4}{-6+3\,\pi\,\alpha}+$$

$$\frac{43\,904\,A\,\gamma^7\,\text{Sin}\left[3\,\pi^2\,\alpha\right]\,\delta_4}{-2+3\,\pi\,\alpha}+\frac{43\,904\,A\,\gamma^7\,\text{Sin}\left[3\,\pi^2\,\alpha\right]\,\delta_4}{2+3\,\pi\,\alpha}+$$

$$\frac{17\,920\,A\,\gamma^7\,\text{Sin}\left[3\,\pi^2\,\alpha\right]\,\delta_4}{6+3\,\pi\,\alpha}+\frac{2240\,A\,\gamma^7\,\text{Sin}\left[3\,\pi^2\,\alpha\right]\,\delta_4}{10+3\,\pi\,\alpha}-\frac{448\,\gamma^8\,\text{Sin}\left[3\,\pi^2\,\alpha\right]\,\delta_4}{-10+3\,\pi\,\alpha}-$$

$$\frac{4480\,\gamma^8\,\text{Sin}\left[3\,\pi^2\,\alpha\right]\,\delta_4}{-6+3\,\pi\,\alpha}-\frac{12\,544\,\gamma^8\,\text{Sin}\left[3\,\pi^2\,\alpha\right]\,\delta_4}{-2+3\,\pi\,\alpha}-\frac{12\,544\,\gamma^8\,\text{Sin}\left[3\,\pi^2\,\alpha\right]\,\delta_4}{2+3\,\pi\,\alpha}-$$

$$\frac{4480\,\gamma^8\,\text{Sin}\left[3\,\pi^2\,\alpha\right]\,\delta_4}{6+3\,\pi\,\alpha}-\frac{448\,\gamma^8\,\text{Sin}\left[3\,\pi^2\,\alpha\right]\,\delta_4}{10+3\,\pi\,\alpha}+\frac{840\,A^4\,\gamma^4\,\text{Sin}\left[4\,\pi^2\,\alpha\right]\,\delta_4}{\pi\,\alpha}+$$

$$\frac{140\,A^4\,\gamma^4\,\mathrm{Sin}[4\,\pi^2\,\alpha]\,\delta_4}{-2+\pi\,\alpha}+\frac{560\,A^4\,\gamma^4\,\mathrm{Sin}[4\,\pi^2\,\alpha]\,\delta_4}{-1+\pi\,\alpha}+\frac{560\,A^4\,\gamma^4\,\mathrm{Sin}[4\,\pi^2\,\alpha]\,\delta_4}{1+\pi\,\alpha}+$$

$$\frac{140\,A^4\,\gamma^4\,\mathrm{Sin}[4\,\pi^2\,\alpha]\,\delta_4}{2+\pi\,\alpha}-\frac{3360\,A^3\,\gamma^5\,\mathrm{Sin}[4\,\pi^2\,\alpha]\,\delta_4}{\pi\,\alpha}-\frac{560\,A^3\,\gamma^5\,\mathrm{Sin}[4\,\pi^2\,\alpha]\,\delta_4}{-2+\pi\,\alpha}-$$

$$\frac{2240\,A^3\,\gamma^5\,\mathrm{Sin}[4\,\pi^2\,\alpha]\,\delta_4}{-1+\pi\,\alpha}-\frac{2240\,A^3\,\gamma^5\,\mathrm{Sin}[4\,\pi^2\,\alpha]\,\delta_4}{1+\pi\,\alpha}-\frac{560\,A^3\,\gamma^5\,\mathrm{Sin}[4\,\pi^2\,\alpha]\,\delta_4}{2+\pi\,\alpha}+$$

$$\frac{5712\,A^2\,\gamma^6\,\mathrm{Sin}[4\,\pi^2\,\alpha]\,\delta_4}{\pi\,\alpha}+\frac{840\,A^2\,\gamma^6\,\mathrm{Sin}[4\,\pi^2\,\alpha]\,\delta_4}{-2+\pi\,\alpha}+\frac{3696\,A^2\,\gamma^6\,\mathrm{Sin}[4\,\pi^2\,\alpha]\,\delta_4}{-1+\pi\,\alpha}+$$

$$\frac{3696\,A^2\,\gamma^6\,\mathrm{Sin}[4\,\pi^2\,\alpha]\,\delta_4}{1+\pi\,\alpha}+\frac{840\,A^2\,\gamma^6\,\mathrm{Sin}[4\,\pi^2\,\alpha]\,\delta_4}{2+\pi\,\alpha}-\frac{4704\,A\,\gamma^7\,\mathrm{Sin}[4\,\pi^2\,\alpha]\,\delta_4}{\pi\,\alpha}-$$

$$\frac{560\,A\,\gamma^7\,\mathrm{Sin}[4\,\pi^2\,\alpha]\,\delta_4}{-2+\pi\,\alpha}-\frac{2912\,A\,\gamma^7\,\mathrm{Sin}[4\,\pi^2\,\alpha]\,\delta_4}{-1+\pi\,\alpha}-\frac{2912\,A\,\gamma^7\,\mathrm{Sin}[4\,\pi^2\,\alpha]\,\delta_4}{1+\pi\,\alpha}-$$

$$\frac{560\,A\,\gamma^7\,\mathrm{Sin}[4\,\pi^2\,\alpha]\,\delta_4}{2+\pi\,\alpha}+\frac{1568\,\gamma^8\,\mathrm{Sin}[4\,\pi^2\,\alpha]\,\delta_4}{\pi\,\alpha}+\frac{140\,\gamma^8\,\mathrm{Sin}[4\,\pi^2\,\alpha]\,\delta_4}{-2+\pi\,\alpha}+$$

$$\frac{896\,\gamma^8\,\mathrm{Sin}[4\,\pi^2\,\alpha]\,\delta_4}{-1+\pi\,\alpha}+\frac{896\,\gamma^8\,\mathrm{Sin}[4\,\pi^2\,\alpha]\,\delta_4}{1+\pi\,\alpha}+\frac{140\,\gamma^8\,\mathrm{Sin}[4\,\pi^2\,\alpha]\,\delta_4}{2+\pi\,\alpha}+$$

$$\frac{448\,A^3\,\gamma^5\,\mathrm{Sin}[5\,\pi^2\,\alpha]\,\delta_4}{-6+5\,\pi\,\alpha}+\frac{1344\,A^3\,\gamma^5\,\mathrm{Sin}[5\,\pi^2\,\alpha]\,\delta_4}{-2+5\,\pi\,\alpha}+\frac{1344\,A^3\,\gamma^5\,\mathrm{Sin}[5\,\pi^2\,\alpha]\,\delta_4}{2+5\,\pi\,\alpha}+$$

$$\frac{448\,A^3\,\gamma^5\,\mathrm{Sin}[5\,\pi^2\,\alpha]\,\delta_4}{6+5\,\pi\,\alpha}-\frac{1344\,A^2\,\gamma^6\,\mathrm{Sin}[5\,\pi^2\,\alpha]\,\delta_4}{-6+5\,\pi\,\alpha}-\frac{4032\,A^2\,\gamma^6\,\mathrm{Sin}[5\,\pi^2\,\alpha]\,\delta_4}{-2+5\,\pi\,\alpha}-$$

$$\frac{4032\,A^2\,\gamma^6\,\mathrm{Sin}[5\,\pi^2\,\alpha]\,\delta_4}{2+5\,\pi\,\alpha}-\frac{1344\,A^2\,\gamma^6\,\mathrm{Sin}[5\,\pi^2\,\alpha]\,\delta_4}{6+5\,\pi\,\alpha}+\frac{1344\,A\,\gamma^7\,\mathrm{Sin}[5\,\pi^2\,\alpha]\,\delta_4}{-6+5\,\pi\,\alpha}+$$

$$\frac{4480\,A\,\gamma^7\,\mathrm{Sin}[5\,\pi^2\,\alpha]\,\delta_4}{-2+5\,\pi\,\alpha}+\frac{4480\,A\,\gamma^7\,\mathrm{Sin}[5\,\pi^2\,\alpha]\,\delta_4}{2+5\,\pi\,\alpha}+\frac{1344\,A\,\gamma^7\,\mathrm{Sin}[5\,\pi^2\,\alpha]\,\delta_4}{6+5\,\pi\,\alpha}-$$

$$\frac{448\,\gamma^8\,\mathrm{Sin}[5\,\pi^2\,\alpha]\,\delta_4}{-6+5\,\pi\,\alpha}-\frac{1792\,\gamma^8\,\mathrm{Sin}[5\,\pi^2\,\alpha]\,\delta_4}{-2+5\,\pi\,\alpha}-\frac{1792\,\gamma^8\,\mathrm{Sin}[5\,\pi^2\,\alpha]\,\delta_4}{2+5\,\pi\,\alpha}-$$

$$\frac{448\,\gamma^8\,\mathrm{Sin}[5\,\pi^2\,\alpha]\,\delta_4}{6+5\,\pi\,\alpha}+\frac{224\,A^2\,\gamma^6\,\mathrm{Sin}[6\,\pi^2\,\alpha]\,\delta_4}{3\,\pi\,\alpha}+\frac{112\,A^2\,\gamma^6\,\mathrm{Sin}[6\,\pi^2\,\alpha]\,\delta_4}{-2+3\,\pi\,\alpha}+$$

$$\frac{112\,A^2\,\gamma^6\,\mathrm{Sin}[6\,\pi^2\,\alpha]\,\delta_4}{2+3\,\pi\,\alpha}-\frac{448\,A\,\gamma^7\,\mathrm{Sin}[6\,\pi^2\,\alpha]\,\delta_4}{3\,\pi\,\alpha}-\frac{224\,A\,\gamma^7\,\mathrm{Sin}[6\,\pi^2\,\alpha]\,\delta_4}{-2+3\,\pi\,\alpha}-$$

$$\frac{224\,A\,\gamma^7\,\mathrm{Sin}[6\,\pi^2\,\alpha]\,\delta_4}{2+3\,\pi\,\alpha}+\frac{256\,\gamma^8\,\mathrm{Sin}[6\,\pi^2\,\alpha]\,\delta_4}{3\,\pi\,\alpha}+\frac{112\,\gamma^8\,\mathrm{Sin}[6\,\pi^2\,\alpha]\,\delta_4}{-2+3\,\pi\,\alpha}+$$

$$\frac{112\,\gamma^8\,\mathrm{Sin}[6\,\pi^2\,\alpha]\,\delta_4}{2+3\,\pi\,\alpha}+\frac{64\,A\,\gamma^7\,\mathrm{Sin}[7\,\pi^2\,\alpha]\,\delta_4}{-2+7\,\pi\,\alpha}+\frac{64\,A\,\gamma^7\,\mathrm{Sin}[7\,\pi^2\,\alpha]\,\delta_4}{2+7\,\pi\,\alpha}-$$

$$\left.\left.\frac{64\,\gamma^8\,\mathrm{Sin}[7\,\pi^2\,\alpha]\,\delta_4}{-2+7\,\pi\,\alpha}-\frac{64\,\gamma^8\,\mathrm{Sin}[7\,\pi^2\,\alpha]\,\delta_4}{2+7\,\pi\,\alpha}+\frac{\gamma^8\,\mathrm{Sin}[8\,\pi^2\,\alpha]\,\delta_4}{\pi\,\alpha}\right)\right)\Big/$$

$$\left(256\left(2\pi^2\left(A^2-2A\gamma+2\gamma^2\right)+\frac{8\pi^2\alpha\,(A-\gamma)\,\gamma\,\mathrm{Sin}\left[\pi^2\alpha\right]}{-4+\pi^2\alpha^2}+\frac{\gamma^2\,\mathrm{Sin}\left[2\pi^2\alpha\right]}{\alpha}\right)\right)$$

$$\begin{aligned}
\tilde{\mu}_{eqv} = {} & \left(64\pi^2\alpha\left(144-328\pi^2\alpha^2+9\pi^4\alpha^4\right)\mu\left(32\alpha\,(A-\gamma)\,\gamma\,\mathrm{Sin}\left[\pi^2\alpha\right]+\right.\right.\\
& \quad \left.\left(-4+\pi^2\alpha^2\right)\left(8A^2-16A\gamma+2\left(4+\pi^2\alpha^2\right)\gamma^2-\alpha\gamma^2\,\mathrm{Sin}\left[2\pi^2\alpha\right]\right)\right)+\\
& \pi^2\alpha^2\left(-576+1456\pi^2\alpha^2-364\pi^4\alpha^4+9\pi^6\alpha^6\right)\gamma^4\,\mathrm{Sin}\left[4\pi^2\alpha\right]\left(-4\gamma 1+\pi^2\alpha^2\psi^2\gamma 2\right)-\\
& 64\pi^2\alpha^2\left(144-40\pi^2\alpha^2+\pi^4\alpha^4\right)(A-\gamma)\,\gamma^3\,\mathrm{Sin}\left[3\pi^2\alpha\right]\\
& \quad\left(\left(-4+3\pi^2\alpha^2\right)\gamma 1+2\pi^2\alpha^2\psi^2\gamma 2\right)+64\pi^2\alpha^2\left(-4+9\pi^2\alpha^2\right)(A-\gamma)\,\gamma\,\mathrm{Sin}\left[\pi^2\alpha\right]\\
& \quad\left(\left(16A^2\left(-20+\pi^2\alpha^2\right)-32A\left(-20+\pi^2\alpha^2\right)\gamma+\left(-464-16\pi^2\alpha^2+\pi^4\alpha^4\right)\gamma^2\right)\gamma 1+\right.\\
& \quad\quad\left.6\left(-128A^2+256A\gamma+\left(-128-36\pi^2\alpha^2+\pi^4\alpha^4\right)\gamma^2\right)\psi^2\gamma 2\right)-\\
& 8\left(144-328\pi^2\alpha^2+9\pi^4\alpha^4\right)\gamma^2\,\mathrm{Sin}\left[2\pi^2\alpha\right]\left(4\left(8-10\pi^2\alpha^2+\pi^4\alpha^4\right)(A-\gamma)^2\gamma 1+\right.\\
& \quad\quad\left.\pi^2\alpha^2\left(-48A^2+96A\gamma+\left(-48-4\pi^2\alpha^2+\pi^4\alpha^4\right)\gamma^2\right)\psi^2\gamma 2\right)+\\
& 4\pi^2\alpha\left(-576+1456\pi^2\alpha^2-364\pi^4\alpha^4+9\pi^6\alpha^6\right)\\
& \quad\left(4\left(4A^4-16A^3\gamma+2A^2\left(16+\pi^2\alpha^2\right)\gamma^2-4A\left(8+\pi^2\alpha^2\right)\gamma^3+3\left(4+\pi^2\alpha^2\right)\gamma^4\right)\gamma 1+\right.\\
& \quad\quad 3\left(16A^4-64A^3\gamma+16A^2\left(6+\pi^2\alpha^2\right)\gamma^2-\right.\\
& \quad\quad\quad\left.\left.\left.32A\left(2+\pi^2\alpha^2\right)\gamma^3+\left(16+16\pi^2\alpha^2+\pi^4\alpha^4\right)\gamma^4\right)\psi^2\gamma 2\right)\right)\Big/\\
& \left(32\pi^2\alpha\,(-6+\pi\alpha)\,(6+\pi\alpha)\,(-2+3\pi\alpha)\,(2+3\pi\alpha)\left(32\alpha\,(A-\gamma)\,\gamma\,\mathrm{Sin}\left[\pi^2\alpha\right]+\right.\right.\\
& \quad\left.\left.\left(-4+\pi^2\alpha^2\right)\left(8A^2-16A\gamma+2\left(4+\pi^2\alpha^2\right)\gamma^2-\alpha\gamma^2\,\mathrm{Sin}\left[2\pi^2\alpha\right]\right)\right)\right)
\end{aligned}$$
